# Identification of an E3 ligase that targets the catalytic subunit of RNA Polymerase I upon transcription stress

**DOI:** 10.1016/j.jbc.2022.102690

**Published:** 2022-11-11

**Authors:** Stephanie Pitts, Hester Liu, Adel Ibrahim, Amit Garg, Catarina Mendes Felgueira, Asma Begum, Wenjun Fan, Selina Teh, Jin-Yih Low, Brittany Ford, David A. Schneider, Ronald Hay, Marikki Laiho

**Affiliations:** 1 Department of Radiation Oncology and Molecular Radiation Sciences, Johns Hopkins University School of Medicine, Baltimore, Maryland, USA; 2 Centre for Gene Regulation and Expression, University of Dundee, Dundee, Scotland, United Kingdom; 3 Department of Biochemistry and Molecular Genetics, University of Alabama at Birmingham, Birmingham, Alabama, USA; 4 Drug Research Program, Faculty of Pharmacy, University of Helsinki, Helsinki, Finland

**Keywords:** transcription, ubiquitin, proteasome, small molecule, cancer, ChIP, chromatin immunoprecipitation, co-IP, coimmunoprecipitation, CRL4, cullin4A–RING E3 ubiquitin ligase, DMSO, dimethyl sulfoxide, DUB, deubiquitinase, FBS, fetal bovine serum, HA, hemagglutinin, IgG, immunoglobulin G, NIH, National Institutes of Health, Pol I, RNA Polymerase I, PVDF, polyvinylidene difluoride, qPCR, quantitative PCR, rDNA, ribosomal DNA, RIPA, radioimmunoprecipitation assay, SCF, Skp–Cullin–F-box protein

## Abstract

RNA Polymerase I (Pol I) synthesizes rRNA, which is the first and rate-limiting step in ribosome biogenesis. Factors governing the stability of the polymerase complex are not known. Previous studies characterizing Pol I inhibitor BMH-21 revealed a transcriptional stress-dependent pathway for degradation of the largest subunit of Pol I, RPA194. To identify the E3 ligase(s) involved, we conducted a cell-based RNAi screen for ubiquitin pathway genes. We establish Skp–Cullin–F-box protein complex F-box protein FBXL14 as an E3 ligase for RPA194. We show that FBXL14 binds to RPA194 and mediates RPA194 ubiquitination and degradation in cancer cells treated with BMH-21. Mutation analysis in yeast identified lysines 1150, 1153, and 1156 on Rpa190 relevant for the protein degradation. These results reveal the regulated turnover of Pol I, showing that the stability of the catalytic subunit is controlled by the F-box protein FBXL14 in response to transcription stress.

RNA Polymerase I (Pol I) is an essential enzyme that transcribes ribosomal DNA (rDNA) into a 47S rRNA precursor. This marks the first rate-limiting step of ribosome biogenesis (1, 2). The precursor rRNA is cotranscriptionally processed into mature 5.8S, 18S, and 28S rRNAs (3). The mature rRNAs are modified by rRNA biogenesis factors and small nucleolar RNAs and assembled, along with the 5S rRNA and ribosomal proteins, into the 40S and 60S eukaryotic ribosomal subunits (4). Ribosome biosynthesis is energetically costly, proportional to cell growth and proliferation, and a fundamental process for cell life. Pol I transcription of rRNA accounts for approximately 60% of all transcriptions. Actively proliferating cells consume an estimated 90% of their metabolic activity on ribosome biogenesis and protein translation (5). In addition, rRNA synthesis is tightly regulated in response to metabolic and environmental changes (2, 6, 7). Pol I activity is required for the maintenance of pluripotency, while its downregulation is required for the differentiation of embryonic stem cells and lineage specification (8, 9, 10, 11). Pathways deregulating Pol I transcription in cancer have been defined and are ubiquitous. Numerous oncogenic drivers, growth factors, and nutrient signaling pathways upregulate Pol I transcription rates by promoting transcription initiation, rRNA processing, and ribosome biogenesis. Conversely, multiple tumor suppressors restrain Pol I activity by negatively regulating the respective steps (6, 12, 13).

Pol I is a multisubunit enzyme that shares structural and functional conservation with Pol II and Pol III (14, 15). As compared with Pol II, the only other polymerase that can transcribe long transcripts, Pol I has a higher elongation rate, a unique 3′ RNA cleavage activity, and is composed of several subunits that are structurally and functionally analogous to Pol II transcription factors (16, 17, 18, 19, 20). Crystal and cryo-EM structures of yeast and human Pol I complexes have been resolved and provide detailed structural characterization of Pol I and the conformational changes that take place during Pol I initiation and elongation (21, 22, 23, 24, 25, 26, 27). The enzyme catalytic core is formed by the two largest subunits, which in mammals are RPA194 and its binding partner, RPA135. These form the DNA-binding cleft that serves as the active site of Pol I. Pol I processivity is facilitated by the enzyme stability. In yeast, the interaction between Rpa190 and Rpa135 is stable (28), and in mammals, RPA194 and RPA135 have a long half-life (>20 h). We previously showed that RPA135 impacts the stability of RPA194, as knockdown (KD) of RPA135 reduces RPA194 protein abundance (29). External factors, such as zinc availability and temperature, mediate Pol I stability in yeast. Zinc depletion induces vacuolar proteolysis of Pol I (30), whereas cold shock affects Rpa190 stability *via* Ubp10-mediated deubiquitination (31).

The large subunit of Pol II, RPB1, is ubiquitinated and degraded in response to stalled transcription complexes. The ubiquitination is mediated by multiple E3 ligases, such as Rsp5 (NEDD4L), the elongin ABC–Rbx1–Cul5 complex, BRCA1/BARD1, WWP2, the cullin4A–RING E3 ubiquitin ligase (CRL4) (CUL4, RBX1, and DDB1) complex, and CUL3 (32, 33, 34, 35, 36, 37, 38, 39, 40, 41, 42, 43, 44). Recent studies have shown that RPB1 ubiquitination at a single lysine, K1268, is a critical mediator of the UV damage–induced degradation of RPB1 and transcription-coupled repair (45, 46). Proteasome-mediated degradation of the largest subunit of yeast Pol III, C160, also occurs upon transcription stalling and is mediated by the Slx5–Slx8 SUMO-targeted E3 ligase complex (47, 48). These data implicate ubiquitination as a regulatory process to control transcription and to resolve transcription elongation blocks.

Given the essential role of ribosome biogenesis in cancer cell growth and the upregulation of Pol I activity in many cancers, Pol I has emerged as a promising target for cancer therapeutics. We recently discovered a first-in-class small molecule, BMH-21, that specifically and selectively blocks Pol I transcription (29, 49, 50, 51). We further described several other small molecules with similar mechanisms of action against Pol I (52, 53). Other chemotherapeutic drugs, such as actinomycin D, oxaliplatin, and topoisomerase II poisons (CX-5461, anthracyclines, and etoposide), also inhibit rRNA synthesis (54, 55, 56, 57, 58, 59, 60). However, BMH-21 is unique in a number of ways. BMH-21 is a DNA intercalator that binds to rDNA noncovalently and does not induce DNA damage (49, 55, 58, 61). It directly blocks both Pol I initiation and elongation and ultimately induces the degradation of RPA194 by the ubiquitin proteasome system. We hypothesize that this degradation pathway is a cellular response to rDNA transcriptional stress; however, the exact mechanisms that govern the stability and inducible degradation of RPA194 remain unknown. Since the stress-induced degradation of the largest subunit is conserved across Pol I, Pol II, and Pol III, this represents an evolutionarily significant means of regulating RNA polymerase activity.

Here, we elucidated the mechanisms of the regulated RPA194 degradation. We undertook a rational, targeted, cell-based RNAi screen to identify E3 ligases involved in the regulation of basal RPA194 turnover and the inducible degradation by BMH-21. The screen and consequent validation experiments identified Skp–Cullin–F-box protein (SCF)^FBXL14^ as an E3 ligase of RPA194. FBXL14 expression sensitized select cancer cells to the therapeutic activity of BMH-21 and led to the degradation of RPA194 in degradation-refractory cancer cell lines. Furthermore, FBXL14 expression sensitized Pol I complex to transcription stress and RPA194 degradation by treatments that do not induce the degradation in parent cells. These findings provide a model of regulated turnover of Pol I that depicts its innate sensitivity to Pol I transcription stress and nominate an essential E3 ligase for the process.

## Results

### An RNAi screen identifies SCF^FBXL14^ as a potential E3 ligase of RPA194

**Figure 1:**
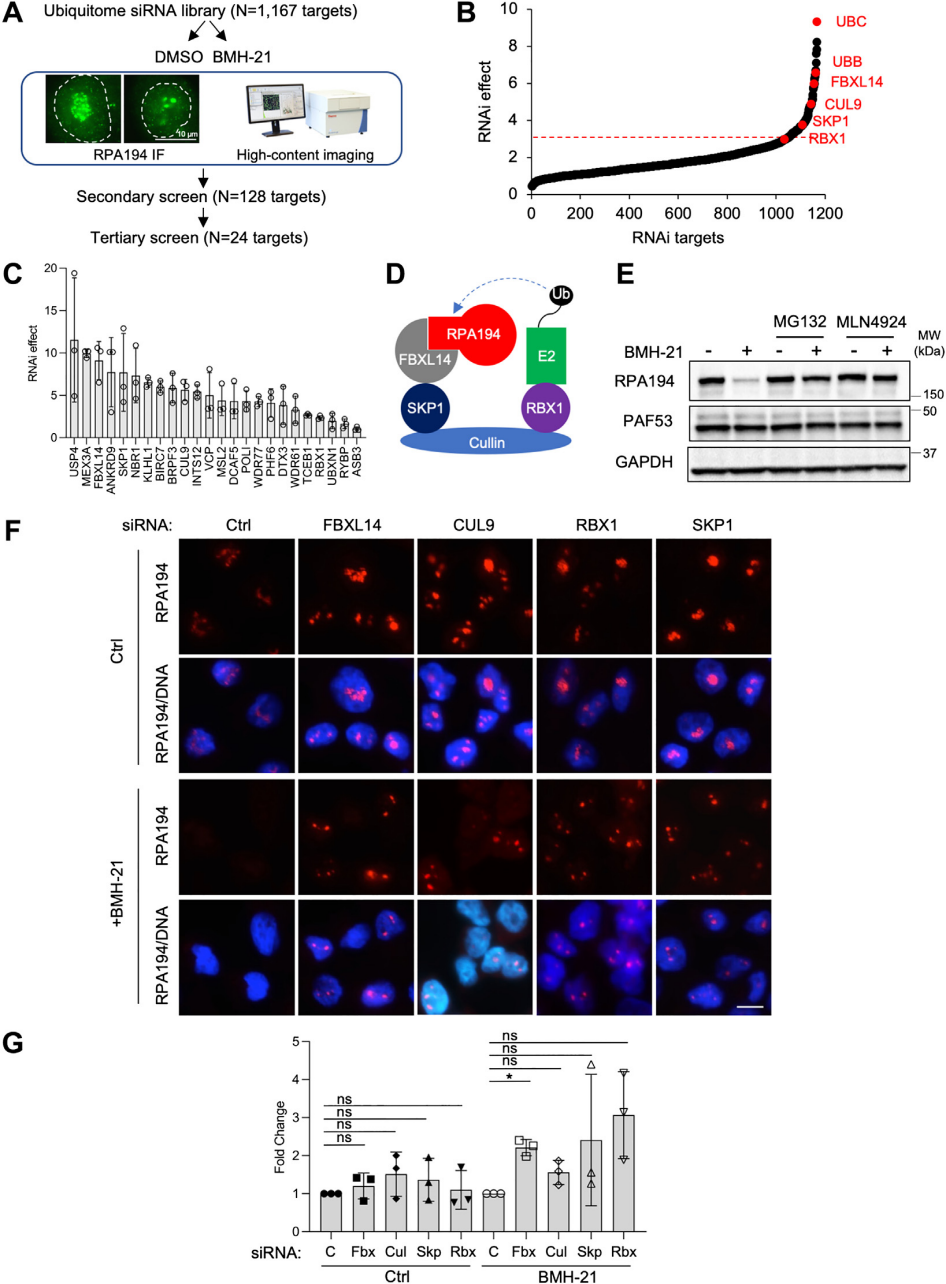
**A cell-based RNAi screen for RPA194 stability identifies SCF**^**FBXL14**^**as a candidate E3 ligase for RPA194.***A*, schematic of RNAi primary and validation screens. *B*, the primary screen results as analyzed for the rescue of BMH-21-mediated degradation of RPA194 (RNAi effect). Select candidates are shown in *red*. The *dashed red line* denotes the threshold for inclusion of hits into the secondary screen. *C*, tertiary screen candidate genes and their RNAi scores. Data are represented as mean ± SD of the tertiary screen replicates (n = 3). *D*, SCF^FBXL14^ complex members identified in the screen. *E*, A375 melanoma cells were treated with BMH-21 (1 μM), MG132 (10 μM), and MLN4924 (1 μM) as indicated for 4 h, and cell lysates were analyzed by immunoblotting. Representative experiment of n = 3 is shown. *F*–*H*, cells were transfected with siRNAs against the SCF complex genes, incubated for 72 h and treated with a vehicle control (DMSO) or BMH-21 (1 μM) for 3 h. *F*, immunofluorescence staining. Representative biological replicate of n = 3 is shown. The scale bar represents 10 μm. *G*, quantification of Western blot analysis for RPA194 protein in (S1*G*). n = 3 biological replicates. The samples were normalized to the Ctrl siRNA-treated cells with or without BMH-21 treatment set as 1 and are represented as mean ± SD. SiRNA control (C), FBXL14 (Fbx), CUL9 (Cul), SKP1 (Skp), and RBX1 (Rbx). *P*, one-sample *t* test. DMSO, dimethyl sulfoxide; SCF, Skp–Cullin–F-box protein.

We previously showed that BMH-21-mediated RPA194 degradation occurs through the ubiquitin–proteasome system (49). To identify the E3 ligase(s) involved in the regulation of RPA194 turnover, we conducted a targeted RNAi screen for ubiquitin pathway genes and used high-content imaging to assess RPA194 protein stability (Figs. 1*A* and S1*A*). U2OS osteosarcoma cells were transfected with a library of siRNAs targeting 1167 known and predicted genes in the ubiquitin pathway (62). The primary screen was conducted using pools of four siRNAs against each target in two biological replicates and three technical replicates. The cells were then treated for 4 h with a vehicle control (dimethyl sulfoxide [DMSO]) or BMH-21 (1 μM) in order to detect changes in the basal expression of RPA194 as well as in the BMH-21-activated turnover. RPA194 protein expression was detected and quantified using immunofluorescence and high-content imaging, with excellent assay performance based on the Z-prime and strictly standardized mean difference analyses of the primary screen. This yielded a robust ranking of candidates that affected both basal RPA194 expression (referred to as “basal regulation”) and RPA194 turnover by the drug (referred to as “RNAi effect”) (Figs. 1*B* and S1, *B*–*D*). Interestingly, ubiquitin molecules were the most highly ranked hits, attesting to the conceptual validity of the approach (Fig. 1*B*). A confirmatory secondary screen was conducted on 128 targets that impacted basal RPA194 abundance by either an increase or a decrease of RPA194 or rescued BMH-21-mediated RPA194 degradation. A tertiary deconvolution screen, where each of the four siRNAs were tested individually, was conducted on 24 candidate genes. Based on this strategy, we compiled rank lists of candidates. KD of several potential E3 ligases led to an increased abundance of RPA194, suggesting that they govern steady-state levels of RPA194 (Fig. S1*E*). Importantly, we identified genes whose KD mitigated the BMH-21-mediated degradation of RPA194 (Fig. 1*C*).

The candidate genes included several known E3 ligases. We focused on genes that affected the drug-inducible turnover. Given that three members of an SCF complex, namely SKP1, FBXL14, and RBX1, were all identified in the screen, we selected this complex for further study (Fig. 1, *C* and *D*). The SCF complexes are a subgroup of the CRLs, the largest family of E3 ubiquitin ligases. The stereotypical SCF complex contains a scaffold protein, a cullin, whose carboxyl terminus binds to an RBX protein and whose amino terminus binds to SKP1 or SKP2. RBX1 is a RING protein that recruits a ubiquitin-charged E2 to the E3 ligase. SKP1 is an adaptor protein that binds to a variable F-box protein. Importantly, the F-box protein identifies and binds to the target/substrate protein (63). Given these findings, we tested the dependency of RPA194 degradation on neddylation, a post-translational modification required for the activation of CRLs (63). Similar to treatment with proteasome inhibitor MG132, treatment of cells with neddylation inhibitor MLN4924 rescued the BMH-21-mediated degradation of RPA194 (Fig. 1*E*). None of these treatments affected RPA194 transcript expression as measured by quantitative PCR (qPCR) (Fig. S1*F*). This supported the finding that a CRL, such as an SCF complex, is involved in RPA194 degradation and substantiated the focus on the identified SCF complex.

We next sought to validate the results of the RNAi screen by individually knocking down each of the identified SCF complex members—SKP1, FBXL14, and RBX1—using two confirmed and independent siRNAs per gene that were different from those used in the screen. Since CUL9 was also identified in the RNAi screen, we tested its involvement in RPA194 degradation as well. We validated the KD efficiency of each gene by qPCR (Fig. S1*G*). We found that knocking down each of these four genes rescued the BMH-21-induced degradation of RPA194, as shown by immunofluorescence analysis (Fig. 1*F*). Western blotting analyses showed prominent and significant attenuation of degradation by silencing FBXL14, whereas silencing of SKP1 and RBX1 had a variable effect and that of CUL9 did not rescue the degradation (Figs. 1*G* and S1*H*). It is therefore unlikely that CUL9 is part of the SCF complex, consistent with previous observations that CUL9 neither interacts with SKP1 nor forms SCF-like complexes (63, 64). Instead, another cullin protein may act as the scaffold of this SCF complex. We therefore chose to pursue mechanistic studies on the relevance of SCF^FBXL14^ in the BMH-21-induced degradation of RPA194.

### Degradation of RPA194 depends on FBXL14

**Figure 2:**
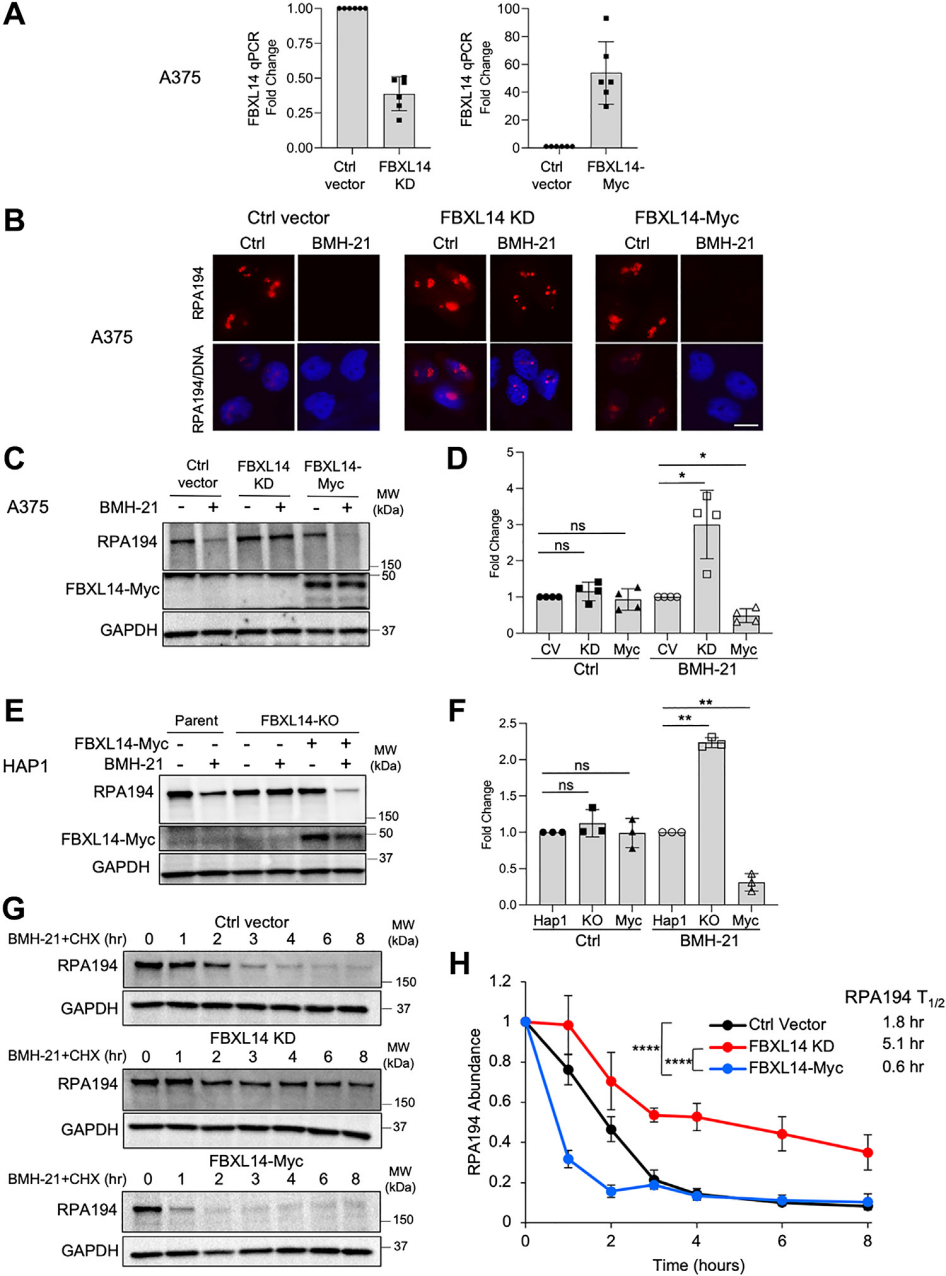
**Inducible degradation of RPA194 depends on FBXL14.***A*, quantitative PCR (qPCR) analysis of FBXL14 mRNA transcript in A375 melanoma cells with stable FBXL14 knockdown (KD) (*left*) or FBXL14-Myc overexpression (Myc) (*right*). Fold change of n = 6 biological replicates. Data are represented as mean ± SD. *B*–*D*, A375 cells were treated with or without BMH-21 (1 μM) for 4 h. *B*, immunofluorescence analysis of RPA194. Representative images of n = 3 biological replicates are shown. The scale bar represents 10 μm. *C*, Western blot analysis. *D*, quantification of n = 4 biological replicates in (*C*). The samples were normalized to the Ctrl vector (CV) expressing cells with or without BMH-21 treatment set as 1 and are represented as mean ± SD. *P*, one-sample *t* test. *E* and *F*, HAP1 parent, FBXL14 KO or KO cells reconstituted with FBXL14-Myc (Myc) leukemia cells were treated with BMH-21 (1 μM) for 16 h. *E*, Western blot analysis. *F*, quantification of n = 3 biological replicates in (*E*). The samples were normalized to the parent HAP1 cells with or without BMH-21 treatment set as 1 and are represented as mean ± SD. *P*, one-sample *t* test. *G* and *H*, half-life analysis of RPA194. A375 cell lines modified for FBXL14 expression were treated with BMH-21 (1 μM) and cycloheximide (CHX) (10 μg/ml), and cell lysates were collected at the times indicated. *G*, Western blot analysis. *H*, quantification of the Western blots of n = 2 to 3 biological replicates in (*G*) and half-life analyses. N = 2 for A375 Ctrl vector and n = 3 for A375 FBXL14 KD and A375 FBXL14-Myc cells. Data are represented as mean ± SD. Statistical significance was determined using two-way ANOVA and Tukey’s post hoc test.

Since the F-box protein is the substrate-specific member of the SCF complex (63), we focused on further validating the role of the identified F-box protein, FBXL14, in RPA194 degradation. To do this, we used an FBXL14 shRNA lentiviral vector to generate stable A375 melanoma and U2OS osteosarcoma cells with effective KD of FBXL14. We also used a lentiviral vector expressing Myc-tagged FBXL14 to generate stable cell lines overexpressing FBXL14 and measured the change in FBXL14 transcript by qPCR (Figs. 2*A* and S2*A*). As shown by immunofluorescence, knocking down FBXL14 strikingly rescued BMH-21-mediated RPA194 degradation, whereas FBXL14 overexpression led to robust degradation (Figs. 2*B* and S2*B*). These findings were confirmed by analyzing RPA194 abundance by Western blotting (Figs. 2, *C*, *D*, S2, *C*, and *D*). To further investigate this, we next undertook analyses in HAP1 chronic myelogenous leukemia cells with a complete CRISPR–Cas9 KO of FBXL14. These cells contain a 1-base pair insertion in exon 1 of the *FBXL14* gene, which yields a frameshift that leads to a premature stop codon in the leucine-rich repeat region of the gene. The HAP1 FBXL14-KO cells showed a substantial rescue of BMH-21-induced RPA194 degradation when compared with the HAP1 parent cells (Fig. 2, *E* and *F*). We then used the FBXL14-Myc tag lentiviral vector to reconstitute FBXL14 expression in the FBXL14-KO cells. As shown in Figure 2, *E* and *F*, reconstitution of FBXL14-Myc induced RPA194 degradation by BMH-21. FBXL14 KD, KO, or overexpression did not affect the basal abundance of RPA194 protein or the expression of the RPA194 transcript (Figs. 2, *D*, *F* and S2*E*). These results demonstrate that the degradation of RPA194 upon induction of transcriptional stress depends on FBXL14.

We previously showed that BMH-21 reduces the half-life of RPA194 from over 20 h to around 1 h (49). To determine whether FBXL14 affects RPA194 expression at the post-translational level, we performed cycloheximide chase experiments. We treated A375 control, FBXL14-KD, and FBXL14-overexpressing cells with cycloheximide and BMH-21 to determine the effect of FBXL14 on the BMH-21-mediated turnover of RPA194. Knocking down FBXL14 significantly increased the half-life of RPA194 from 1.8 to 5.1 h, and overexpression of FBXL14 significantly reduced RPA194 half-life to 0.6 h (Fig. 2, *G* and *H*). These findings show that FBXL14 affects RPA194 protein turnover in response to transcriptional stress.

### FBXL14 interacts with RPA194 and affects its ubiquitination

**Figure 3:**
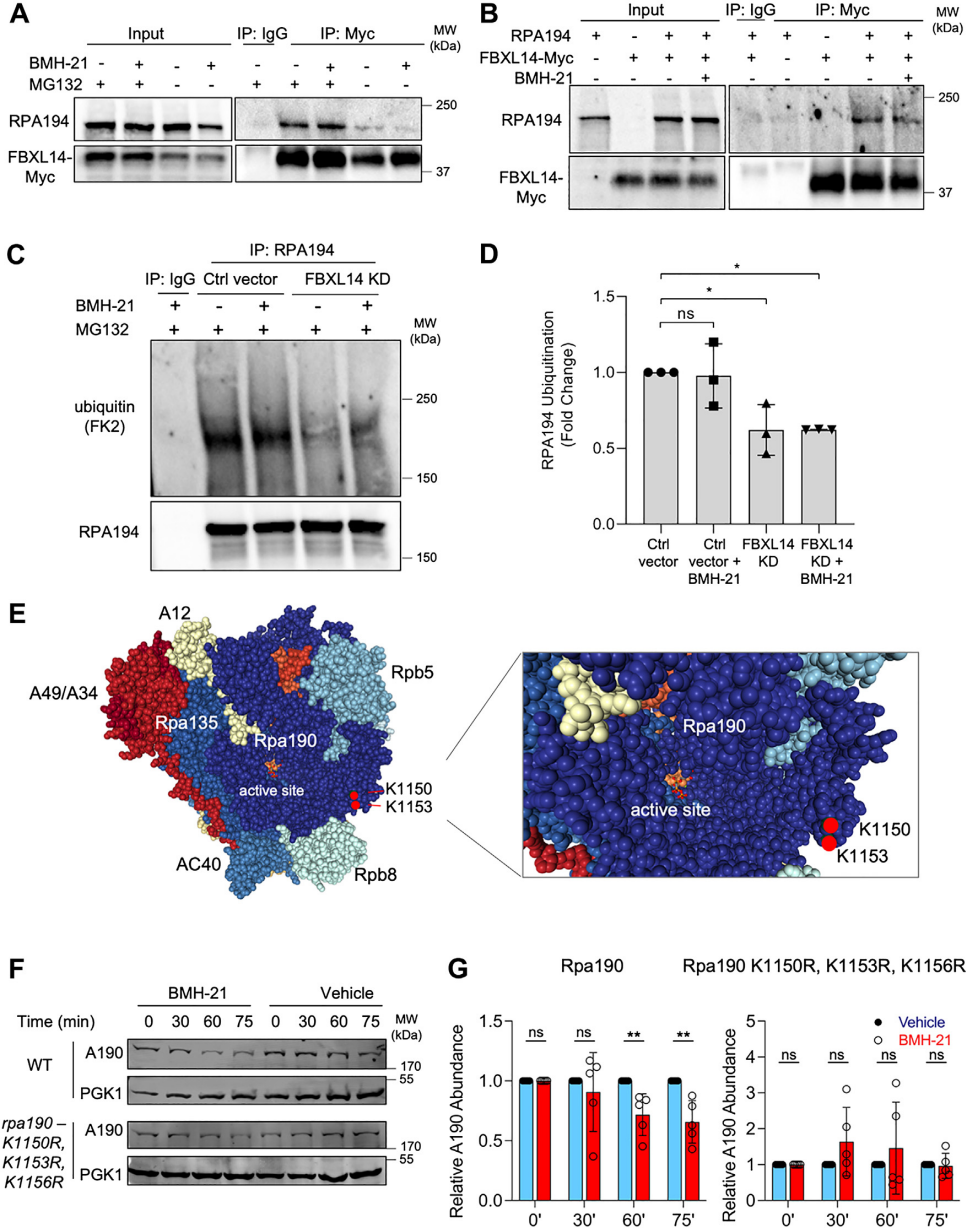
**FBXL14 interacts with RPA194 and mediates RPA194 ubiquitination.***A*, A375 cells stably expressing RPA194-HA and FBXL14-Myc were treated as indicated (BMH-21, 1 μM and MG132, 10 μM) for 4 h. The lysates were precipitated with a Myc-tag antibody and blotted for RPA194 and FBXL14-Myc. A representative experiment of n = 3 biological replicates is shown. *B*, *in vitro* interaction analysis. RPA194 and FBXL14-Myc proteins were *in vitro* translated and incubated for 30 min at 30 °C, followed by precipitation with a Myc-tag antibody and blotting for RPA194 and FBXL14-Myc. A representative experiment of n = 3 biological replicates is shown. *C*, A375 control (Ctrl vector) and FBXL14-knockdown (FBXL14 KD) cells were treated with BMH-21 (1 μM) and MG132 (10 μM) for 4 h as indicated. Cell lysates were precipitated with an RPA194 antibody and blotted for ubiquitin (FK2 antibody) and RPA194. *D*, RPA194 ubiquitination was quantified from n = 3 biological replicates in (*C*). Data are represented as mean ± SD. Statistical significance was determined using one-way ANOVA and Tukey’s post hoc test. *E*, structure of the yeast Pol I complex (RNA-binding domain: 6HKO) highlighting putative K1150 and K1153 sites of ubiquitination. K1156 is not visible in the structure. *F*, WT and Rpa190 lysine-mutant yeast strains were treated with a vehicle control (0.1 M NaH_2_PO_4_) or BMH-21 (50 μM) for the indicated times. Western blot analyses were conducted. *G*, quantification of the Western blots of n = 5 biological replicates in (*F*). Data are represented as mean ± SD. Statistical significance was determined using nonparametric Mann–Whitney in RStudio. HA, hemagglutinin.

An E3 ligase typically binds to its substrate, catalyzes its ubiquitination, and induces its degradation (65). Since we confirmed that FBXL14 induces the degradation of RPA194, we next analyzed whether the proteins interact. To study the interaction between the two proteins, we used A375 cells stably expressing hemagglutinin (HA)-tagged RPA194 and Myc-tagged FBXL14. We performed coimmunoprecipitation (co-IP) experiments in which we precipitated ectopic FBXL14-Myc and blotted for RPA194. As shown in Figure 3*A*, RPA194 coprecipitated with FBXL14-Myc, both in the presence and absence of BMH-21. The amount of FBXL14 and coprecipitated protein was enhanced by treatment with the proteasome inhibitor MG132, whereas the absence of MG132 treatment led to a sharp decrease in coprecipitated RPA194. We infer that the proteasome-mediated degradation of RPA194 led to a reduction of RPA194 protein with which FBXL14 could interact.

We also noticed that FBXL14 expression was lower in the absence of MG132, suggesting that FBXL14 expression is regulated by the proteasome as well. Furthermore, treatment with neddylation inhibitor MLN4924 stabilized FBXL14 (Fig. S3*A*), suggesting that a CRL is also involved in FBXL14 degradation. To assess whether FBXL14 turnover is affected by BMH-21, we conducted cycloheximide chase experiments to assess FBXL14 stability. As all tested FBXL14 antibodies had inadequate performance in recognizing the endogenous protein, we used the stable A375 cell lines overexpressing FBXL14-Myc for this approach. The half-life of FBXL14-Myc was relatively short, 43 min, and it was unaffected by BMH-21 treatment (Fig. S3, *B* and *C*). Thus, FBXL14 turnover is independent of transcriptional stress.

To examine the protein–protein interaction of RPA194 and FBXL14 *in vitro*, we *in vitro* translated HA-tagged RPA194 and Myc-tagged FBXL14. Equal amounts of the proteins were incubated together for 30 min at 30 °C, and the resulting mixture was precipitated with an antibody against the Myc tag and blotted for RPA194. We observed that RPA194 was coprecipitated with FBXL14-Myc (Fig. 3*B*). These experiments demonstrate that RPA194 and FBXL14 interact both in cells and *in vitro*.

To determine whether FBXL14 affects RPA194 ubiquitination, we performed co-IP experiments in which we precipitated RPA194 and detected ubiquitination using antibodies against ubiquitin. We conducted these experiments in the presence of proteasome inhibitor MG132 to avoid RPA194 turnover by BMH-21. We found that RPA194 ubiquitination was significantly higher in control cells than in FBXL14-KD cells (Fig. 3, *C* and *D*). This effect was independent of transcriptional stress. This indicates that RPA194 ubiquitination is dependent on FBXL14.

We next questioned the site(s) of ubiquitination on RPA194. Prior large-scale proteomics analyses of human cancer cells indicated two sites of ubiquitination, K1180 and K1184, on RPA194 (66). We performed site-directed mutagenesis of these sites and generated stable cells expressing the HA-tagged K1180 and K1184 RPA194 double mutant. However, the expression of the mutant was too low to render a co-IP feasible, possibly given the presence of and competition by endogenous RPA194 (not shown). To facilitate the assessment of potential ubiquitination sites, we chose to use the *Saccharomyces cerevisiae* model system, in which we have previously shown that the turnover of the large catalytic subunit after treatment with BMH-21 is conserved (29). Furthermore, detailed structural analyses of the elongating Pol I complex are available in yeast (14, 15, 24). The homologous putative sites of ubiquitination in yeast Rpa190 (the yeast homolog of RPA194) are K1153 and K1156. These sites are especially interesting given that they reside in the vicinity of the mobile trigger loop in the polymerase active center. In addition, they are optimally positioned on an exposed surface of Rpa190 in the enzyme foot, rendering these sites accessible for ubiquitination (Fig. 3*E*). We mutated yeast Rpa190 residues K1153 and K1156 and adjacent K1150 to arginine and expressed this allele as the sole source of Rpa190 to determine the effect on BMH-21-induced Rpa190 degradation. While wildtype Rpa190 was degraded upon BMH-21 treatment, the *rpa190-K1150R-K1153R-K1156R* variant was not (Fig. 3, *F* and *G*). These sites are thus involved in the stress-induced Rpa190 degradation.

### FBXL14 overexpression is associated with increased response to BMH-21-mediated inhibition of growth in A375 melanoma cells

**Figure 4:**
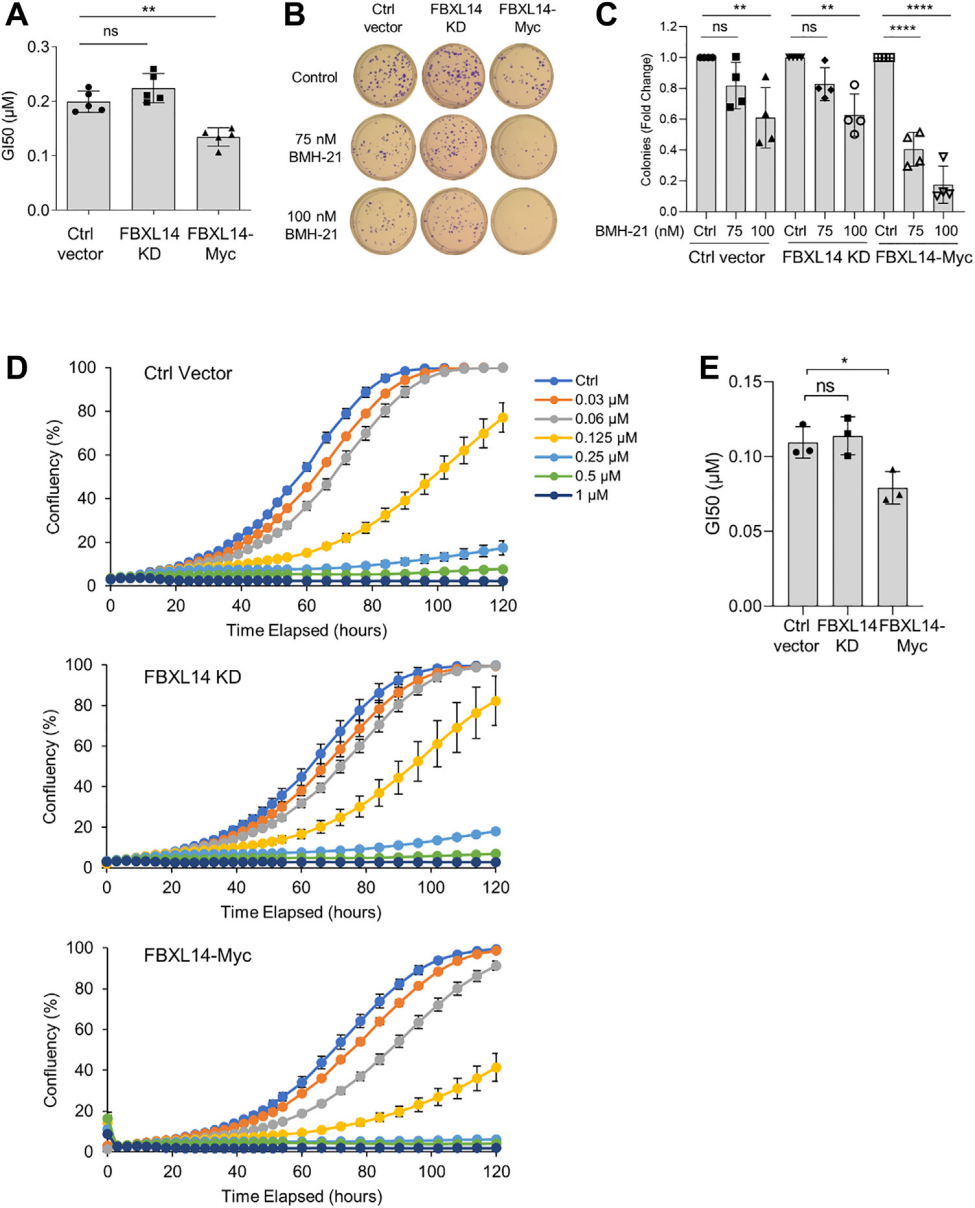
**FBXL14 overexpression is associated with increased sensitivity of A375 cells to BMH-21.***A*, A375 control (Ctrl vector), FBXL14-knockdown (FBXL14 KD), and FBXL14-overexpressing (FBXL14-Myc) cells were treated with half-log concentrations of BMH-21 for 3 days, and cell viability was determined. GI_50_ was determined of n = 5 biological experiments. Data are represented as mean ± SD. Statistical significance was determined using one-way ANOVA and Tukey’s post hoc test. *B*, A375 Ctrl vector, FBXL14 KD, and FBXL14-Myc cells were treated with BMH-21 for 7 days, and the plates were fixed and stained. *C*, the mean number of colonies of n = 4 biological experiments in (*B*) was counted. Data are represented as mean ± SD. Statistical significance was determined using one-way ANOVA and Tukey’s post hoc test. *D*, A375 Ctrl vector, FBXL14 KD, and FBXL14-Myc cells were treated with the indicated concentrations of BMH-21 and live-cell measurements were obtained over 5 days. Representative biological replicates are shown. Mean ± SE are shown. *E*, quantification of BMH-21 GI_50_ of n = 3 biological experiments in (*D*). Data are represented as mean GI_50_ at 84 h ± SD. Statistical significance was determined using one-way ANOVA and Tukey’s post hoc test.

We previously found that BMH-21-mediated RPA194 degradation correlates with BMH-21-mediated cell death (49). We therefore wanted to determine whether FBXL14 affects not only RPA194 degradation but also sensitivity to BMH-21. To measure this sensitivity, we used three orthogonal methods: cell viability assays, clonogenic assays, and live-cell analyses. These experiments were performed in A375 melanoma cells, whereas FBXL14 was stably knocked down or overexpressed. Dose titrations with BMH-21 were performed. As observed using all three approaches, FBXL14 KD did not affect sensitivity to BMH-21 (Fig. 4, *A*–*E*). However, we consistently observed that FBXL14 overexpression, which enhanced RPA194 degradation, sensitized the A375 melanoma cells to loss of viability and growth by BMH-21 as measured by a decrease in the GI_50_ and a reduction in the number of surviving colonies upon BMH-21 treatment (Fig. 4, *A*–*E*). To further determine the effect of FBXL14 depletion on sensitivity to BMH-21, we assessed cell viability in the HAP1 FBXL14-KO cells. Similar to the A375 FBXL14-KD cells, FBXL14 KO did not affect sensitivity to BMH-21. Re-expression of FBXL14-Myc in the HAP1 FBXL14-KO cells also had no effect on sensitivity to BMH-21 (Fig. S4*A*). The genetic modifications of FBXL14 had a negligible effect on the growth of the A375 cells without the drug (Fig. S4, *B* and *C*).

### FBXL14 overexpression induces BMH-21-mediated RPA194 degradation in cancer cell lines refractory to the degradation

**Figure 5:**
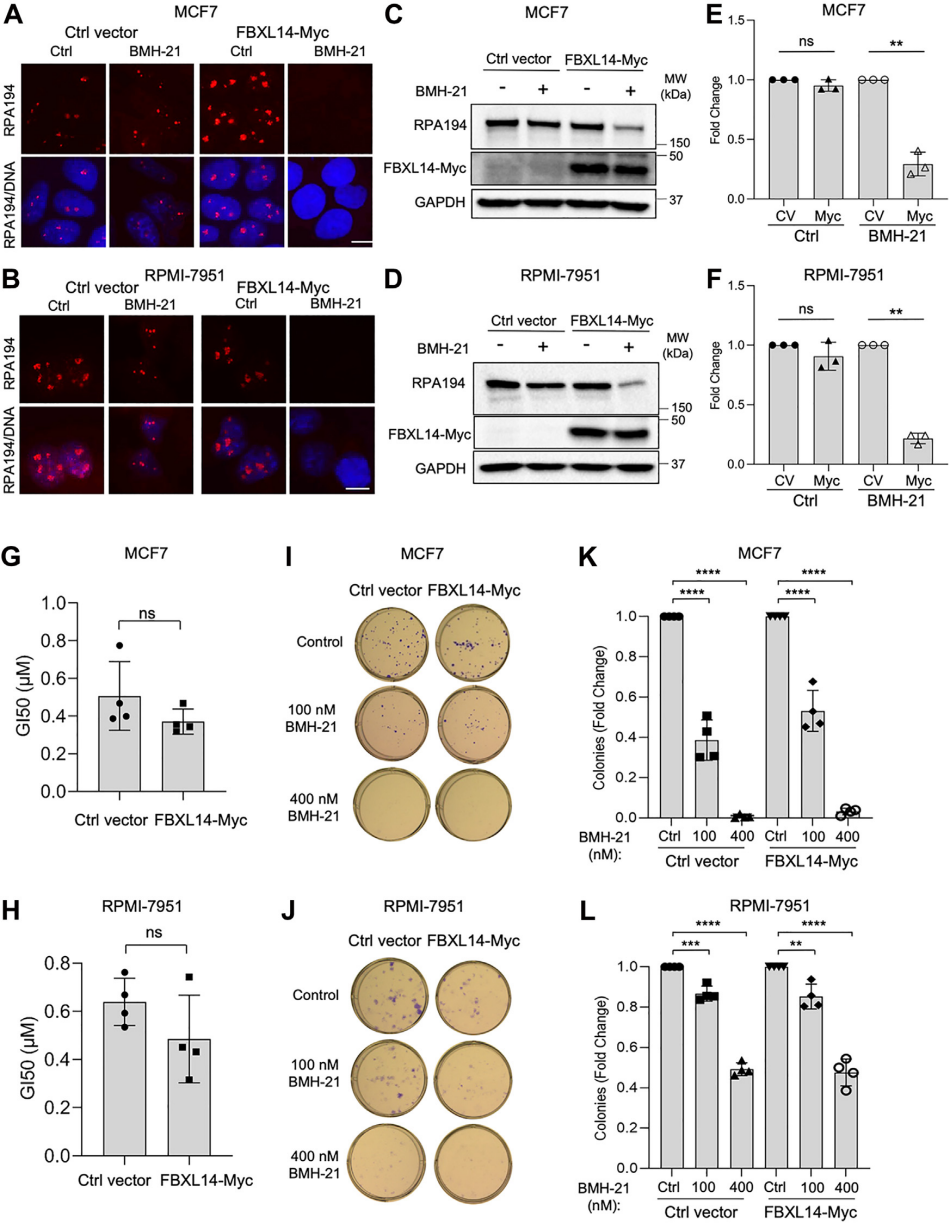
**FBXL14 overexpression induces BMH-21-mediated RPA194 degradation in refractory cancer cell lines.** FBXL14-Myc (Myc) or ctrl vector (CV) was stably expressed in MCF7 breast adenocarcinoma (*A*, *C*, *E*, *G*, *I*, and *K*) and RPMI-7951 melanoma (*B*, *D*, *F*, *H*, *J*, and *L*) cells. Cells were treated with or without BMH-21 (1 μM) for 4 h. *A* and *B*, RPA194 immunofluorescence. Representative biological replicates of n = 3 are shown. The scale bar represents 10 μm. *C* and *D*, Western blotting. *E* and *F*, quantification of n = 3 biological replicates in (*C* and *D*), respectively. The samples were normalized to the Ctrl vector expressing cells with or without BMH-21 treatment set as 1 and are represented as mean ± SD. Vehicle control (C), BMH-21 (+B). *P*, one-sample *t* test. *G* and *H*, cells were incubated with half-log concentrations of BMH-21 for 3 days, cell viability was determined, and GI_50_ was calculated from n = 4 biological replicates. Data are represented as mean ± SD. Statistical significance was determined using an unpaired *t* test. *I* and *J*, cells were treated with BMH-21 for 12 days, fixed, and stained. *K* and *L*, the mean number of colonies of n = 4 biological replicates in (*I* and *J*) was counted. Data are represented as mean ± SD. Statistical significance was determined using one-way ANOVA and Tukey’s post hoc test.

We earlier showed that cancer cell lines differed in their response to BMH-21-induced RPA194 degradation (49). MCF7 breast adenocarcinoma and RPMI-7951 melanoma cells were refractory to this degradation despite effective inhibition of pol I transcription. We hence asked whether the overexpression of FBXL14 affects BMH-21-mediated RPA194 degradation. To investigate this, we generated MCF7 and RPMI-7951 cells stably overexpressing FBXL14 (Fig. S5, *A* and *B*). We then used immunofluorescence and Western blotting analyses to assess the effect of FBXL14 overexpression on RPA194. Overexpression of FBXL14 distinctly and significantly activated BMH-21-mediated RPA194 degradation in both cell lines (Fig. 5, *A*–*F*). Again, there was no effect of FBXL14 on basal RPA194 abundance (Fig. 5, *E* and *F*).

MCF7 and RPMI-7951 cells are less sensitive to the BMH-21-mediated cell killing than A375 cells (49). To measure whether FBXL14 affected this response, we performed cell viability and clonogenic assays. FBXL14 overexpression did not significantly change the ability of BMH-21 to reduce cell growth (Fig. 5, *G*–*L*). This result was in contrast to the A375 melanoma cells, in which FBXL14 overexpression both enhanced RPA194 degradation and further sensitized cells to BMH-21. It further suggests that the underlying mechanisms of resistance to cell killing in these cell lines are independent of RPA194 turnover.

### Inhibition of Pol I transcription and dissociation of the complex from rDNA by BMH-21 are independent of RPA194 degradation

**Figure 6:**
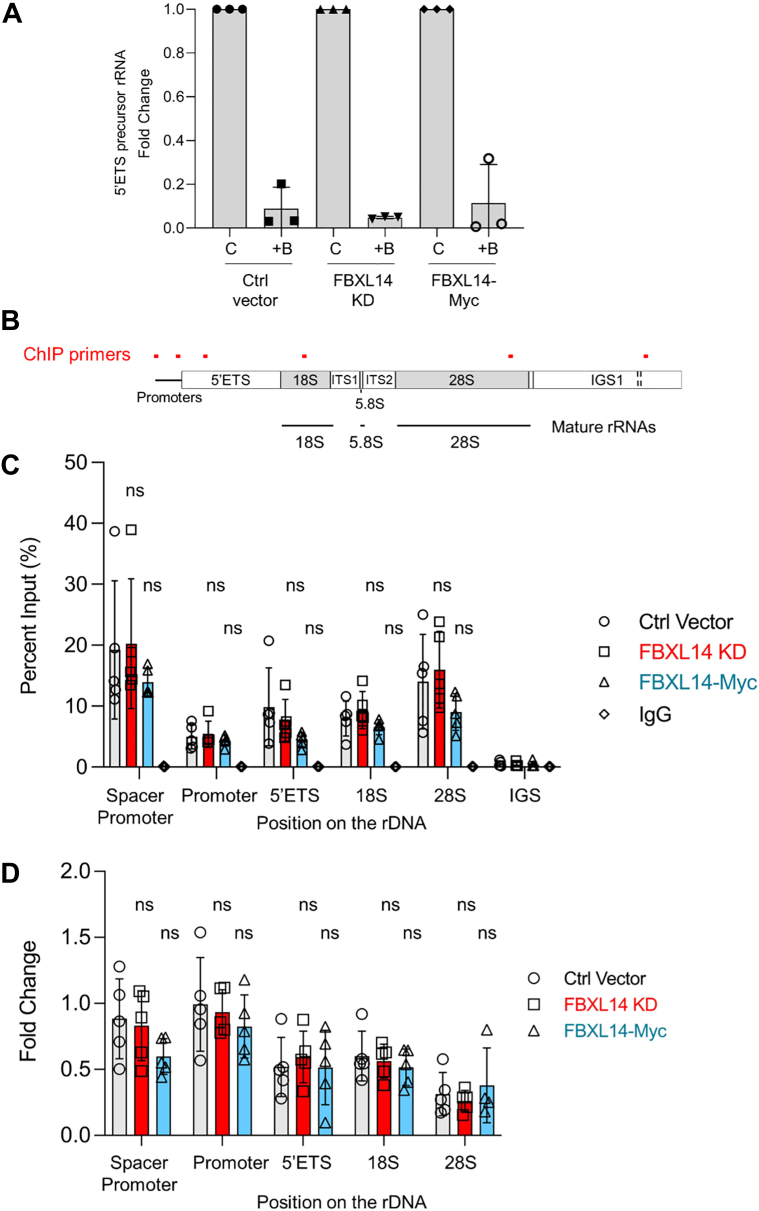
**BMH-21-induced inhibition ofPol I and dissociation of the complex from rDNA are independent of RPA194 degradation.***A*, qPCR analysis of 45S rRNA precursor (5′ETS). A375 cells modified for FBXL14 expression were treated with BMH-21 (1 μM) for 4 h (n = 3 biological replicates). Data are represented as mean ± SD. Vehicle control (*C*), BMH-21 (+B). *B*, diagram of the human rRNA coding locus, indicating the location of the ChIP primers. *C* and *D*, ChIP was conducted using pulldown of RPA194 or IgG on A375 ctrl vector, FBXL14 KD, and FBXL14-Myc cells. Primers for the rDNA promoters and gene body are indicated below. Results represent n = 5 biological replicates. Data are represented as mean ± SD. Statistical significance among the three cell lines was determined using one-way ANOVA and Tukey’s post hoc test. *D*, cells were treated with vehicle (DMSO) or BMH-21 (1 μM) for 30 min. Results represent fold change of the BMH-21-treated compared with the vehicle-treated cells. ChIP, chromatin immunoprecipitation; DMSO, dimethyl sylfoxide; IgG, immunoglobulin G; Pol I, RNA Polymerase I; qPCR, quantitative PCR; rDNA, ribosomal DNA.

We previously demonstrated that the transcription inhibition of Pol I by BMH-21 leads to the disengagement of Pol I complex from the rDNA (29, 49). Pol I transcription was inhibited within just minutes of BMH-21 treatment, whereas the degradation of RPA194 was observed after several hours (29). This observation clearly demonstrates that Pol I transcription is inhibited by the compound independently of RPA194 degradation. Here, we wanted to test this premise using the models generated in this study for the FBXL14-mediated turnover of RPA194. We also considered the possibility that the degradation occurs on the rDNA, prompting the dissociation of Pol I from chromatin. Alternatively, Pol I dissociation could be required for the subsequent degradation of RPA194. To determine whether FBXL14 expression affects Pol I transcription inhibition by BMH-21, we measured Pol I transcription in A375 cells with genetically modified FBXL14 expression. We found that BMH-21 inhibited Pol I transcription to the same extent in all FBXL14-modified cell lines, demonstrating that Pol I is inhibited regardless of the extent of RPA194 degradation (Fig. 6*A*). There was no discernible difference in transcription of the 45S precursor rRNA, mature 18S rRNA, and mature 28S rRNA among the cell lines without the drug treatment (Fig. S6*A*).

We next performed chromatin immunoprecipitation (ChIP) to analyze the enrichment of RPA194 on the rDNA before and after BMH-21 treatment using primers on the rRNA coding region (Fig. 6*B*). We first compared whether the expression of FBXL14 affects basal enrichment of Pol I on chromatin between the cell lines. While there was a trend that the overexpression of FBXL14 diminished the engagement of Pol I on the Spacer promoter and gene body, this finding was not statistically significant (Fig. 6*C*). We then treated the cells with BMH-21 for 30 min to capture temporal events preceding the robust degradation. RPA194 engagement with the rDNA decreased to a similar extent in all three cell lines within 30 min of BMH-21 treatment, indicating that Pol I dissociates from the rDNA regardless of the expression of FBXL14 (Fig. 6*D*). This confirms our previous results (29) and further supports the model that Pol I dissociation from the rDNA occurs prior to RPA194 degradation. Finally, we performed ChIP to query whether FBXL14 could be detected on the rDNA. The level of detectable FBXL14-Myc on the rDNA with or without BMH-21 treatment was negligible (Fig. S6*B*). Taken together, these results demonstrate that RPA194 degradation is not required for BMH-21-mediated repression of rDNA transcription. Rather, treatment with BMH-21 revealed a pathway by which cells modulate RPA194 abundance in response to acute transcriptional stress.

### FBXL14 augments RPA194 turnover by transcription stress

**Figure 7:**
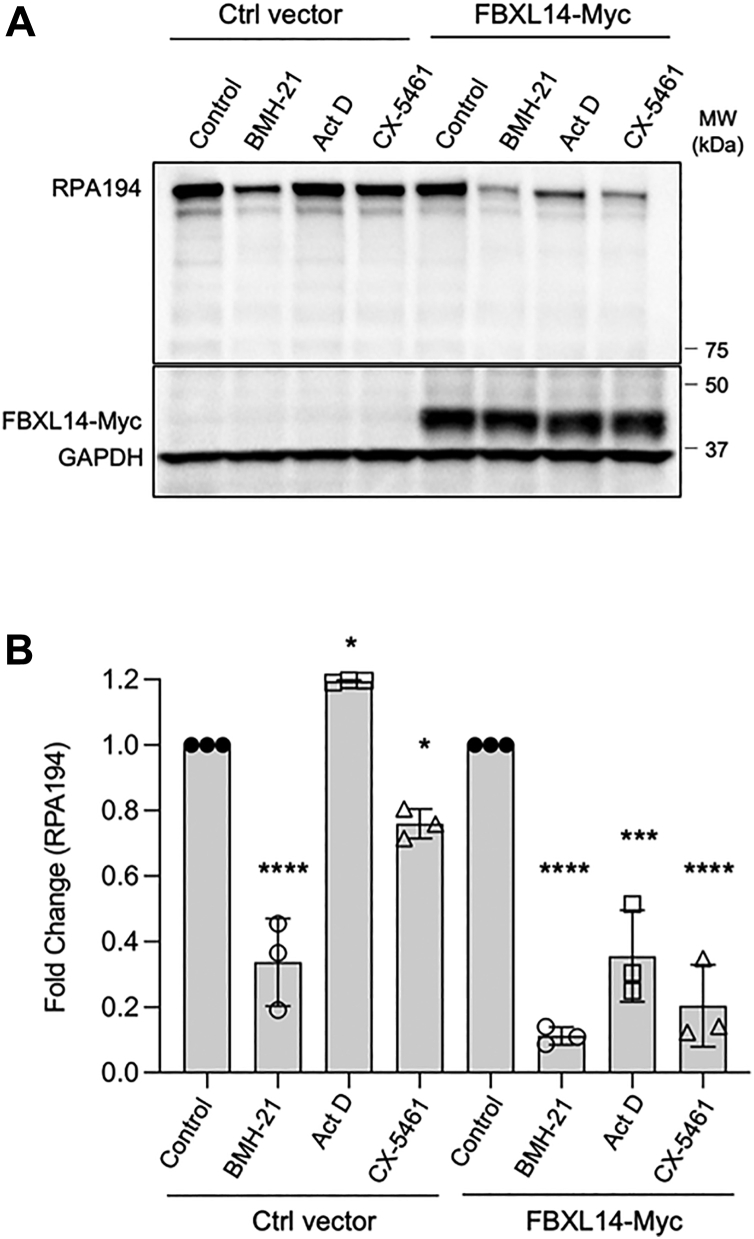
**FBXL14 augments RPA194 turnover by transcription stress.***A*, A375 Ctrl vector and FBXL14-Myc cells were treated with vehicle (DMSO), BMH-21 (1 μM), actinomycin D (ActD) (40 nM), or CX-5461 (1 μM) for 4 h. *B*, quantification of n = 3 biological replicates in (*A*). Data are represented as mean ± SD. Statistical significance was determined using one-way ANOVA and Tukey’s post hoc test. DMSO, dimethyl sulfoxide.

The BMH class of compounds are exceptional in their capacity to activate the regulated turnover of RPA194. Several other molecules that interfere with Pol I transcription by blocking topoisomerases, such as CX-5461, or covalent intercalation with rDNA, such as actinomycin D, do not destabilize Pol I complex. To test the impact of FBXL14 on the turnover by these other drugs, we used the cells with genetically modified FBXL14. Strikingly, as shown in Figure 7, ectopic expression of FBXL14 destabilized RPA194 following treatment by either actinomycin D or CX-5461. This finding suggests that FBXL14 is a key E3 ligase that functions in the governance of RPA194 stability in response to transcriptional insults.

## Discussion

This study was facilitated by the discovery of a unique small molecule, BMH-21, which induces the degradation of RPA194, the catalytic subunit of Pol I complex (49). The use of this molecule allowed us to investigate this novel transcriptional stress–induced pathway for RPA194 turnover. Using a cell-based RNAi screen and further validation studies, we identified SCF^FBXL14^ as an E3 ligase of RPA194. This was demonstrated by the strict dependency of the inducible RPA194 turnover on FBXL14 in cancer cell lines and the ability of FBXL14 expression to affect RPA194 ubiquitination. Expression of FBXL14 also led to the BMH-21-mediated degradation of RPA194 in two cancer cell lines refractory to this effect. Also, it destabilized RPA194 in response to other inhibitors causing Pol I transcription stress. Furthermore, FBXL14 overexpression sensitized A375 melanoma cells to BMH-21-mediated cell death. A detailed understanding of the degradation process provides essential knowledge about the stability of Pol I complex and benefits therapeutic implementation of Pol I inhibitory strategies.

Our screen was designed to identify ubiquitin–proteasome pathway regulators that affect both the basal abundance as well as the inducible turnover of RPA194. Given that three putative members of an SCF complex were identified in the screen and that FBXL14 was the topmost validated candidate in the deconvolution screen rescuing the inducible turnover, SCF^FBXL14^ arose as a top candidate. We were able to confirm the dependency of RPA194 turnover on FBXL14, RBX1, and SKP1, whereas the cullin subunit of the SCF complex remains to be decisively determined. Since CUL9 was identified in our screen, we investigated whether it was involved in RPA194 degradation. KD of CUL9 had little effect on RPA194 degradation, so it is unlikely that CUL9 is part of the SCF complex. This is consistent with previous studies that CUL9 neither interacts with SKP1 nor forms SCF-like complexes (63, 64). Future studies should address the cullin involved in BMH-21-mediated RPA194 degradation. Our screen also identified a number of additional effectors of the proteasomal and proteolytic pathways previously associated with Pol I. For example, PHF6 has been shown to regulate Pol I transcription (67), and VCP (valosin-containing protein) has been implicated in proteolytic degradation of Rpa190 in yeast (30). Also, we ranked the candidates according to their ability to affect basal RPA194 turnover. For example, KD of USP4 deubiquitinase (DUB) decreased basal RPA194 abundance, implicating a protective effect of USP4 on RPA194 degradation. On the other hand, DTX3 and WDR61 KDs were linked with increased RPA194 abundance and are hence nominated as additional E3 ligases for RPA194. Of these, WDR61 is a component of the Ski complex functioning in mRNA quality control and clearance of stalled ribosomes (68).

The identification of these candidates facilitates subsequent studies on their activities as regulators of Pol I complex. Given the ample evidence of multiple E3 ligases regulating the turnover of Pol II by RPB1 monoubiquitination and polyubiquitination (69, 70, 71), we expect that several E3 ligases and DUBs also operate on RPA194. Furthermore, it is reasonable to expect that there are cell type–specific differences in the expression of these proteins that dictate which degradation factors are operational. Such selectivity can especially occur in cancers where many proteasomal turnover enzymes are highly deregulated (72). We find that FBXL14 KD significantly increased the half-life of RPA194 but did not completely eliminate the BMH-21-mediated RPA194 degradation. Similarly, FBXL14 KD significantly reduced but did not eliminate RPA194 ubiquitination. It is therefore plausible that multiple E3 ligases are responsible for the degradation of RPA194, and other candidates should be explored in future studies. Nevertheless, the identification of one E3 ligase, SCF^FBXL14^, sets an important precedent for the regulated turnover of RPA194 in response to acute transcriptional stress.

We have previously shown that the activities of BMH-21 in blocking Pol I transcription and initiating the large subunit degradation are fully conserved in humans and *S. cerevisiae* (29, 50). Large-scale proteomic studies in mammalian cells have suggested RPA194 lysines 1180 and 1184 as potential sites of ubiquitin conjugation (66). These sites are conserved in yeast and located on an exposed surface of the foot next to the trigger loop (24). This location is consequential, as it is not only accessible for modifying enzymes but also could affect the enzyme function. Exploiting these features, we mutated the respective sites in yeast and tested for turnover of Rpa190. The *rpa190-K1150R-K1153R-K1156R* mutant was fully resistant to the BMH-21-induced degradation. These findings identify the ubiquitination sites on Rpa190. Despite the conservation of the inducible turnover of RPA194/Rpa190, we do not know the identity of the yeast E3 ligase, as FBXL14 is not conserved in yeast.

Our earlier studies demonstrated that BMH-21 induces the dissociation of Pol I from the rDNA within 30 min in mammalian cells and significantly reduces the occupancy within 2 min in yeast. RPA194 degradation becomes biochemically measurable within hours of treatment (29, 50). The current study confirmed these findings and further showed that BMH-21-mediated Pol I inhibition and dissociation from the rDNA occur regardless of the extent of RPA194 degradation. These findings support the model that the degradation of RPA194 is a downstream event. It is therefore unlikely that the enzyme is directly targeted for turnover while residing on the rDNA. Rather, the alterations of the enzyme occupancy result from the preceding steps of blocking the loading of the enzyme and the decreased pool of enzyme available. In accordance, we did not detect any specific association of FBXL14 on rDNA or change thereof following treatment by BMH-21. This suggests that the destabilization of RPA194 likely occurs on an enzyme complex that either cannot be assembled correctly or that the drug treatment leads to the dissociation of the complex. As we have earlier shown that the interaction of RPA194 and RPA135 is stable even in the presence of the drug (29), we favor the model where the unsuccessful assembly of pol I sensitizes RPA194 for rapid turnover. From a metabolic perspective, this is a rational outcome, as it conserves building blocks *via* proteasome-mediated recycling when transcription stress is profound. It further resembles the increased proteasome-mediated turnover of newly synthesized ribosomal proteins when rRNA synthesis is halted (73, 74).

We earlier showed differences in cancer cell responses to the BMH-21-activated turnover of RPA194 (49). Here, we show that the expression of FBXL14 activates RPA194 degradation in these refractory cell lines. This reaffirms the impact of FBXL14 as a critical factor mediating the degradation. We further examined whether the increased degradation of RPA194 also increased the sensitivity of cancer cells to the BMH-21-mediated cell killing. If Pol I dissociates regardless of RPA194 degradation, why would the degradation affect the degree of cell death at all? Perhaps the increased degradation significantly reduces the amount of RPA194 recycled to new polymerase complexes for future Pol I transcription cycles. However, the enhanced sensitivity was only observed in A375 cells inherently sensitive to the drug. We speculate that the varied responses result from differences in the intrinsic vulnerability of the cancer cells to not only Pol I transcription stress but also their ability to tolerate the ensuing translational stress and the activity of other survival and antiapoptotic pathways.

We find no evidence that FBXL14 regulates Pol I in the absence of perturbation. RPA194 abundance is unaffected by the genetic modification of FBXL14, as is Pol I transcription. While we did observe a trend of decreased Pol I occupancy on the gene body in cells overexpressing FBXL14, this difference was not statistically significant. Then why is the turnover so prominently activated by BMH-21? First, Pol I transcription has enormous redundancy given the multicopy nature of rDNA loci. In any given human cell, transcription can occur simultaneously on ∼200 copies. Transcription lesions encountered on one copy, even if leading to degradation of a few Pol I complexes, will bear negligible effect on the total RPA194 available. Also, given the ability of the polymerases to backtrack, bypass the lesion, and resolve the lesions by transcription-coupled nucleotide excision repair, a few lesions are likely recoverable. However, when profound inhibition of transcription occurs, especially when multiple transcription cycle steps are perturbed, this leads to activation of the polymerase destruction. This concept is analogous to the degradation of the Pol II RPB1 subunit, where unresolved DNA lesions lead to polyubiquitination and degradation of RPB1 and provide a means for the survival of the cell (70, 71). In yeast, Pol I Rpa190 is degraded upon exposure to low temperatures. It is thought that the cold temperature causes elongation blocks, and the ubiquitin-mediated degradation of Rpa190 serves as a mechanism to resolve this blockage (31). The identified yeast DUB, Upb10, is the ortholog to the mammalian DUB, USP36, that we had identified earlier to block RPA194 degradation (31, 49). Hence, only extreme stressors, simultaneously blocking ongoing transcription on all rRNA genes, will trigger the proteasome-mediated regulation of Pol I. Most strikingly, increased expression of FBXL14 activated the degradation of RPA194 also by other Pol I inhibitors, such as actinomycin D and CX-5461, that lack the capacity to do so otherwise. This finding indicates that FBXL14 acts as a critical and rate-limiting factor that launches/triggers the degradation process when transcription stress is encountered.

Here, we identify the first E3 ligase targeting the Pol I complex. This knowledge provides fundamental insight into the stability of the Pol I complex in response to transcription stress. These aspects are critical for understanding the regulation of the polymerase during physiological and pathophysiological stresses and in the therapeutic implementation of Pol I inhibitory strategies.

## Experimental procedures

### Cell culture and reagents

The following cell lines were obtained from the American Type Culture Collection: A375 melanoma (CRL-1619), U2OS osteosarcoma (HTB-96), MCF7 breast adenocarcinoma (HTB-22), and RPMI-7951 melanoma (HTB-66) and authenticated by short tandem repeat analyses at the Johns Hopkins Genetic Resources Core Facility. The HAP1 chronic myelogenous leukemia parental cell line and the HAP1 FBXL14-KO cell line were obtained from Horizon Discovery. The HAP1 FBXL14-KO cell line was edited with CRISPR–Cas9 to contain a one base pair insertion in exon 1 of the *FBXL14* gene. This insertion yielded a frameshift and a premature stop codon in the leucine-rich repeat region of the gene. All cells were grown at 37 °C in a humidified atmosphere containing 5% CO_2_. A375 and U2OS cells were cultured in Dulbecco's modified Eagle's medium supplemented with 10% fetal bovine serum (FBS), and MCF7 and RPMI-7951 cells were cultured in minimum essential medium supplemented with 10% FBS and nonessential amino acids. HAP1 and HAP1 FBXL14-KO cells were cultured in Iscove’s modified Dulbecco's media supplemented with 10% FBS.

The following reagents were used: MG132 (MilliporeSigma), MLN4924 (Calbiochem), cycloheximide (Calbiochem), DMSO (MilliporeSigma), *N*-ethylmaleimide (Sigma–Aldrich), actinomycin D (MilliporeSigma), and CX-5461 (Selleck Chemicals). 12H-Benzo[g]pyrido[2,1-b]quinazoline-4-carboxamide and *N*-[2(dimethylamino)ethyl]-12-oxo (BMH-21) was synthesized as described (53) and verified for purity using LC/MS and ^1^H NMR spectroscopy.

The following lentiviral KD and expression vectors were used: pLKO.1 empty vector, pLKO.1-shRNA-FBXL14 to 257448 (Sigma; catalog no.: TRCN0000257448), pLenti-FBXL14-C-Myc-DDK-P2A-Puro (Origene; catalog no.: RC207066L3), and pLenti-GIII-CMV-POLR1A (RPA194)-HA (Applied Biological Materials; catalog no.: 371660110000). For *in vitro* translation of RPA194, full-length human RPA194 from the pCR4-TOPO-RPA194 (Invitrogen) plasmid was subcloned into a pCMV6-AC-HA-His (Origene) backbone to generate a pCMV6-AC-RPA194-HA-His plasmid. The resulting plasmid was sequence verified. pCMV6-FBXL14-Myc-DDK was from Origene (catalog no.: RC207066).

### RNAi screen

An RNAi screen was conducted for ubiquitin pathway genes associated with RPA194 degradation. A custom siRNA library targeting 1167 known and predicted genes in the ubiquitin pathway was used (62). The library was composed of Dharmacon ON-TARGET Plus siRNAs, and each target gene was represented by four siRNAs in the primary screen. U2OS cells (2500 cells/well in a 96-well plate) were reverse transfected with 10 nM of siRNA and 0.1 μl of Lipofectamine RNAiMax (Invitrogen), incubated for 72 h, and then treated for 4 h with a vehicle control (DMSO) or BMH-21 (1 μM). Screen plates included the following controls: untransfected wells without siRNA and without primary antibody, nontargeting siRNA controls (siNT), and as positive controls, siRNAs targeting RPA194. Cells were fixed with paraformaldehyde, permeabilized, blocked with PBS/bovine serum albumin/chicken serum and stained with primary antibody (1 μg/ml) against RPA194 (C-1; Santa Cruz; catalog no.: sc-48385) and secondary antibody Alexa488-conjugated antimouse at 1:1500 dilution (Invitrogen), CellMask Deep Red plasma membrane stain (Invitrogen), and DNA (4′,6-diamidino-2-phenylindole).

After immunofluorescence, RPA194 protein expression was quantified by high-content imaging. High-content imaging was conducted on an InCell 2000 system (GE Healthcare) using 20× magnification. Images of four independent fields per well were acquired, yielding on average 600 to 1500 cells per well. The images were analyzed with the InCell analyzer software, and the total area of RPA194 signal within the DNA mask was collected. The RPA194 area was then normalized to the average RPA194 signal in siNT-treated wells in each plate. The RNAi effect was defined as the normalized RPA194 total area (+BMH-21)/normalized RPA194 total area (DMSO).

The primary screen was conducted in two biological replicates (62, 75). The average Z-prime for all plates was 0.560. The primary screen results and Z-prime and strictly standardized mean difference for each screen plate are provided in Table S1. The secondary screen included a total of 128 candidates from the primary screen, including 68 whose RNAi effect was over 3.2, 30 candidates whose siRNA increased basal RPA194 abundance >2, and 30 candidates whose siRNA reduced basal RPA194 abundance <0.5 (Fig. S1). A tertiary screen was conducted on 24 candidate genes of interest by testing each of the four siRNAs individually.

### RNAi

Cells were transfected with 10 nM of siRNA using Lipofectamine RNAiMax and incubated for 48 to 72 h at 37 °C. The following siRNAs (Ambion, Thermo Fisher Scientific) were used: nontargeting control (4390844), FBXL14 (s44684 and s225687), CUL9 (s23061 and s23062), RBX1 (s19386 and s19387), and SKP1 (s12889, s12890, and s12891).

### Generation of stable cell lines

A second-generation lentiviral system (psPAX2 viral packaging vector and pMD2.G viral envelope vector) was used to produce lentiviruses in human embryonic kidney 293FT cells. Fugene6 (Promega) or FugeneHD (Promega) was used as the transfection reagent. Viral media were harvested at 48 and 72 h, pooled together, and used to transduce the cells of interest. After transduction, cells were selected in the presence of puromycin. Pooled cells were maintained under puromycin selection thereafter.

### qPCR

RNA was extracted from cells using Trizol and chloroform and purified with isopropanol. RNA was reverse transcribed using SuperScript II Reverse Transcriptase (Invitrogen). To perform qPCR, the resulting complementary DNA was mixed with PowerUp SYBR Green Master Mix (Applied Biosystems by Thermo Fisher Scientific) and the appropriate primer pairs. Analyses were conducted in triplicate using the BioRad CFX384 Real-Time System—C1000 Touch Thermal Cycler or the Applied Biosystems QuantStudio 12K Flex Real-Time PCR System. All results were normalized to GAPDH, and RNA levels were quantified using the ΔΔCt method.

The following primer pairs were used: FBXL14 (forward: CACCGGCATAGACCTGTA CG, reverse: CCAGGTTGAGTACCTTGAGGC), CUL9 (forward: AGAAGGATGAAGGCCGA ACC, reverse: AATGTGGAGGCCCTTTTCCC), RBX1 (forward: ACGACAGACCGTGTGTTT CC, reverse: GGGGTATCCACATCCATCGC), SKP1 (forward: CCTGAGGAGATTCGCAAGA CC, reverse: CTGTGTGCTACCTACCTGGG), 5′ETS (forward: GAACGGTGGTGTGTCGTT, reverse: GCGTCTCGTCTCGTCTCACT), 18S (forward: CCCGAAGCGTTTACTTTGAA, reverse: CGGTCCAAGAATTTCACCTC), 28S (forward: TGGGTTTTAAGCAGGAGGTG, reverse: AACCTGTCTCACGACGGTCT), RPA194 (forward: GCGTGGTGACTCCGGGCTTG, reverse: CAGGCCGTTTGCCGATGGGT), and GAPDH (forward: GGCCTCCAAGGAGTAAG ACC, reverse: AGGGGTCTACATGGCAACTG).

### Western blotting

Cells were lysed in radioimmunoprecipitation assay (RIPA) lysis buffer (MilliporeSigma) supplemented with protease inhibitor (MilliporeSigma). Protein concentrations were measured using the Pierce BCA Protein Assay Kit (Thermo Fisher Scientific). An equal amount of protein per sample was balanced with RIPA lysis buffer, Laemmli sample buffer (Bio-Rad), and DTT. After boiling, the samples were run on an NuPAGE 3 to 8% Tris-Acetate gel (Invitrogen) and transferred to a polyvinylidene difluoride (PVDF) (0.45 μm) membrane. The membrane was blocked with 5% milk and then blotted with the primary and secondary antibodies. The primary antibodies used were RPA194 (C-1; Santa Cruz; catalog no.: sc-48385), Myc-tag clone 4A6 (Millipore; catalog no.: 05-724), PAF53 (ProteinTech; catalog no.: 16145-1-AP), and GAPDH (abcam; catalog no.: ab8245). Horseradish peroxidase–conjugated secondary antibodies were purchased from Dako. Secondary antibodies were detected using either SuperSignal West Pico PLUS Chemiluminescent Substrate (Thermo Fisher Scientific) or Western Lightning Plus-ECL Enhanced Chemiluminescence Substrate (PerkinElmer) and imaged using the Bio-Rad Molecular Imager ChemiDoc XRS+ System and Image Lab Software. Protein densitometry analysis was conducted on the Image Lab Software.

### Immunofluorescence

Cells were grown on coverslips and fixed with 3.5% paraformaldehyde. The cells were then permeabilized with 0.5% NP-40, blocked with 5% bovine serum albumin, and stained with primary RPA194 antibody (C-1; Santa Cruz; catalog no.: sc-48385) and secondary Alexa594-conjugated antimouse (Invitrogen) antibody. DNA was stained using Hoechst 33258. Images were acquired using the Zeiss Axio Imager Z1 microscope.

### Co-IP and *in vitro* translation

Cells were lysed in RIPA lysis buffer (MilliporeSigma) supplemented with protease inhibitor (MilliporeSigma). Protein concentrations were measured using the Pierce BCA Protein Assay Kit, and 1.5 mg of protein per sample was diluted in 0.5% NP-40 buffer (0.5% NP-40, 50 mM Tris–HCl [pH 7.5], 150 mM NaCl, and 1× protease inhibitor). After preclearing with DiaMag protein G–coated magnetic beads (Diagenode), the samples were incubated with 2 μg of Myc 4A6 (Millipore; catalog no.: 05-724) primary antibody overnight at 4 °C, collected on DiaMag protein G–coated magnetic beads, and washed extensively with 0.5% NP-40 buffer. The bead–antibody–protein complexes were resuspended in 35 μl of Laemmli sample buffer (Bio-Rad) and DTT, and the samples were boiled and run on a NuPAGE 3 to 8% Tris-Acetate gel. The gel was transferred to a PVDF (0.45 μm) membrane and probed for the indicated antibodies. The secondary antibody used was horseradish peroxidase–conjugated light chain–specific antimouse (Cell Signaling Technology; catalog no.: 58802S). As a negative control, one sample was incubated overnight with 2 μg of normal mouse immunoglobulin G (IgG) (MilliporeSigma; catalog no.: 12-371) rather than the Myc 4A6 primary antibody.

For *in vitro* analyses, RPA194 and FBXL14-Myc proteins were *in vitro* translated using the TNT T7 Coupled Reticulocyte Lysate System (Promega; catalog no.: L4610). The following plasmids were used: pCMV6-AC-RPA194-HA-His and pCMV6-FBXL14-Myc-DDK. Equal amounts of the two proteins were mixed, incubated for 30 min at 30 °C, precleared with DiaMag protein G–coated magnetic beads, and incubated overnight with 2 μg of Myc 4A6 primary antibody. The rest of the co-IP reaction was carried out identically to that described previously for A375 cells.

### Ubiquitination assays

Cells were lysed in RIPA lysis buffer (MilliporeSigma) supplemented with protease inhibitor (MilliporeSigma) and 10 mM *N*-ethylmaleimide. Lysates (1.25 mg of protein per sample) were diluted in 0.5% NP-40 buffer, precleared with DiaMag protein A-coated magnetic beads (Diagenode), and incubated with 2 μg of RPA194 primary antibody overnight at 4 °C. Samples were incubated the next day with DiaMag protein A-coated magnetic beads, and the bead–antibody–protein complexes were washed extensively with 0.5% NP-40 buffer. The bead–antibody–protein complexes were resuspended in 35 μl of Laemmli sample buffer (Bio-Rad) and DTT, and the samples were boiled and run on a NuPAGE 3 to 8% Tris-Acetate gel. Along with the input, the precipitated protein was transferred to a PVDF (0.45 μm) membrane and probed first for FK2 ubiquitin (MilliporeSigma; catalog no.: 04-263) and then for RPA194. As a negative control, one sample was incubated overnight with 2 μg of normal mouse IgG (MilliporeSigma; catalog no.: 12-371) rather than the RPA194 primary antibody.

### Yeast Rpa190 analyses

Yeast cells were grown in yeast extract peptone dextrose. During log phase (absorbance of ∼0.3), cells were treated with 50 μM of BMH-21 or an equal volume of vehicle (0.1 M NaH_2_PO_4_) for the indicated times. Western blot analysis was performed as described previously (29) using anti-HA 12CA5 (made in house) and anti-PGK1 22C5D8 (Thermo Fisher Scientific; catalog no.: 459250) primary antibodies.

The following W303-1a derivative yeast strains were used:

*MAT*a *leu2-3112 trp1-1 can1-100 ura3-1 ade2-1 his3-11,15 rpa190Δ::HIS3* carrying either plasmid pDAS1093 or pDAS1096.

DAS1093: DH5α carrying RPA190_TEV_3HA_10his WT −500 to +500 cloned into pRS316 EcoRI to KpnI sequence confirmed (trigger loop area) amp-r.

DAS1096: DH5α carrying RPA190_TEV_3HA_10his K1150R, K1153R, K1156R ubiquitin mutant −500 to +500 cloned into pRS316 EcoRI to KpnI sequence confirmed amp-r.

### Cell viability assays

Cells were plated in 96-well plates at a density of 2000 cells per well. After 24 h, the cells were treated in triplicate with half-log concentrations of BMH-21 and incubated for 3 days. Viability was assessed using CellTiter-Blue Reagent (Promega) and analyzed on the Molecular Devices SpectraMax M5 plate reader. GI_50_ was calculated using GraphPad Prism software (GraphPad Software, Inc).

### Clonogenic assays

Cells were plated in 6-well plates at a density of 100 cells per well (A375 cells) or 300 cells per well (MCF7 and RPMI-7951 cells) and treated with DMSO (vehicle control) or BMH-21 at the indicated concentrations the following day. After 7 days (for A375 cells) or 12 days (for MCF7 and RPMI-7951 cells) of treatment, the plates were fixed with 10% formalin and stained with 0.05% crystal violet. Colonies were photographed and counted using the Interscience Scan 4000 colony counter or manually.

### Live-cell imaging

Cells were plated in 24-well plates at a density of 5000 cells per well. After 24 h, the cells were treated in triplicate with the indicated concentrations of BMH-21 or vehicle control. Live-cell imaging was conducted on IncuCyte ZOOM (Sartorius). Images were acquired every 3 h for the first 48 h and every 6 h afterward. Percent cell confluency was calculated using IncuCyte 2016B analysis software.

### ChIP

Cells (1.78 × 10^7^) were fixed with 1.1% formaldehyde and lysed using the iDeal ChIP-seq kit (Diagenode; catalog no.: C01010170). After chromatin isolation, the chromatin was sheared using the Covaris ME220 Focused-ultrasonicator. Each IP was conducted using 25 μg of chromatin, 2 μg of primary antibody, and secondary antibody–coupled DiaMag magnetic beads (Diagenode). The following primary antibodies were used: RPA194, Myc 4A6, and normal mouse IgG. After washing and reverse crosslinking, the coprecipitated chromatin was purified using iPure beads V2. qPCR was conducted using PowerUp SYBR Green Master Mix (Applied Biosystems by Thermo Fisher Scientific) and the appropriate primer pairs. Samples were run in triplicate on the Applied Biosystems QuantStudio 12K Flex Real-Time PCR System. All results were normalized to 1% input chromatin, and the amount of chromatin coprecipitated was quantified using the percent input method.

The following primer pairs were used: Spacer Promoter (forward: AGGTTTATGTGGG GGAGAGG, reverse: GGCCTCGGGAGCTACG), Promoter (forward: GAGGTATATCTTTCG CTCCGAGTC, reverse: CAGCAATAACCCGGCGG), 5′ETS (forward: GAACGGTGGTGTGT CGTT, reverse: GCGTCTCGTCTCGTCTCACT), 18S (forward: CCCGAAGCGTTTACTTTG AA, reverse: CGGTCCAAGAATTTCACCTC), 28S (forward: TGGGTTTTAAGCAGGAGGTG, reverse: AACCTGTCTCACGACGGTCT), and IGS (forward: ACTGGCGAGTTGATTTCTGG, reverse: CGAGACAGTCGAGGGAGAAG).

### Statistical analysis

All experimentation was conducted using a minimum of three independent biological replicates. In addition, three technical replicates were performed for qPCR, ChIP, cell viability assays, clonogenic assays, and live-cell growth experiments. ANOVA and/or *t* tests were carried out using Excel or GraphPad Prism software. The test used for each experiment is indicated in the figure legend. The *p* values were expressed as follows: ns = not significant, ∗ = *p* < 0.05, ∗∗ = *p* < 0.01, ∗∗∗ = *p* < 0.001, and ∗∗∗∗ = *p* < 0.0001.

## Data availability

All data have been included within the article and supporting figures.

## Supporting information

This article contains supporting information.

## Conflict of interest

M. L., H. L., A. B., and W. F. hold patents or patent applications on Pol I inhibitors, which are managed by the Johns Hopkins University. All other authors declare that they have no conflicts of interest with the contents of this article.

## Supporting information

Supporting information

Uncropped gels
