# Supplementary material for: Identification of an E3 ligase that targets the catalytic subunit of RNA Polymerase I upon transcription stress
Source: J Biol Chem. 2022 Nov 11;298(12):102690. doi: 10.1016/j.jbc.2022.102690 (PMC9727647; doi:10.1016/j.jbc.2022.102690)
Supplement: Supporting information [file mmc2.pdf]

SUPPORTING INFORMATION

Identification of an E3 ligase that targets the catalytic subunit of RNA polymerase I upon transcription stress

Stephanie Pitts, Hester Liu, Adel Ibrahim, Amit Garg, Catarina Mendes Felgueira, Asma Begum, Wenjun Fan, Selina Teh, Jin-Yih Low, Brittany Ford, David A. Schneider, Ronald Hay, and Marikki Laiho

Supporting information contents:

Supplementary Figures S1-S6.
Supplementary Table S1.

Supplementary Figures

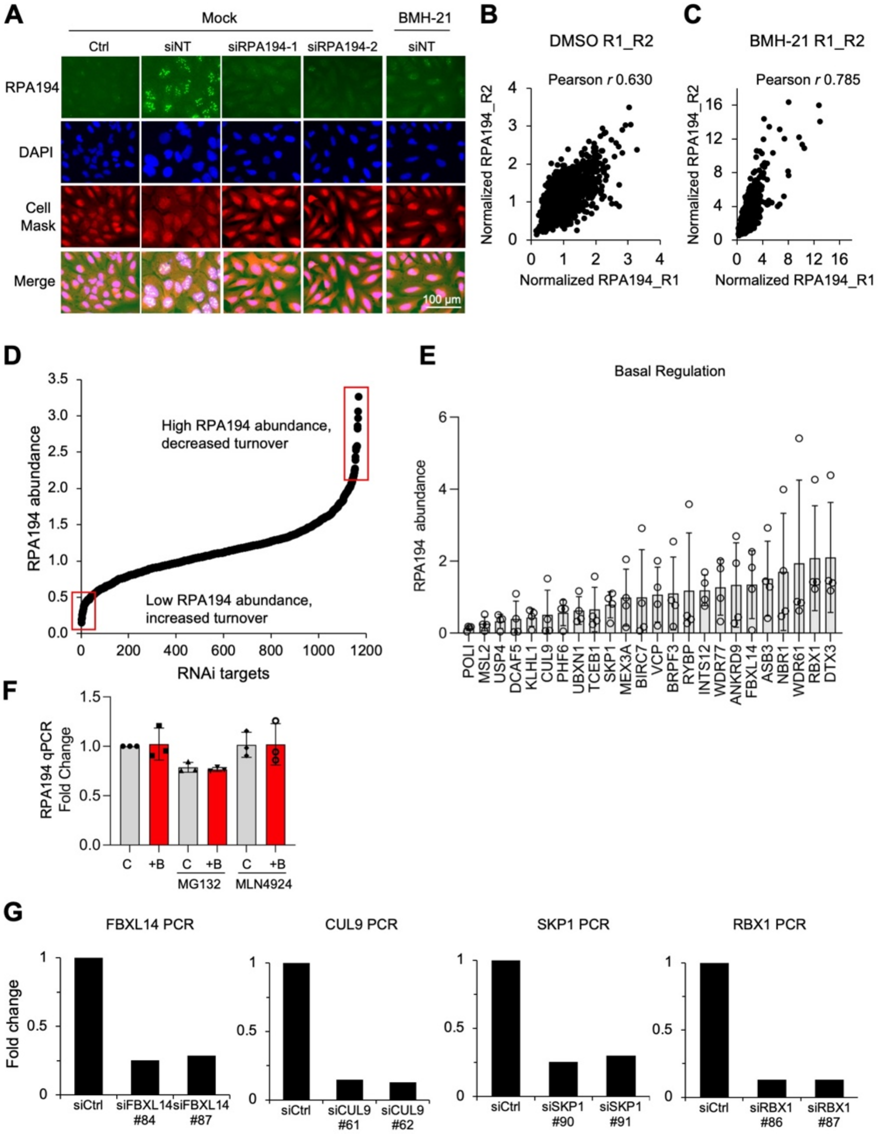

Figure S1. RNAi screen and knockdown efficiency of the SCF^{FBXL14} complex members. *A*, High-content imaging was conducted on an InCell 2000 system to detect RPA194 protein by immunofluorescence in DMSO (vehicle)-treated and BMH-21-treated U2OS osteosarcoma cells followed by quantitative image analysis. Images were acquired using 20x objective. Scale bar, 100 µm. Nuclei and cells were stained using DAPI and CellMask dyes, respectively. *B, C*, Pearson correlation analyses of the two biological replicates (R1, R2) in DMSO- treated (*B*) and BMH-21 treated plates (*C*) of the primary screen. *D*, Primary screen scoring for RPA194 protein abundance. *Red* boxes indicate candidates included in the secondary screen. *E*, Tertiary screen results for RPA194 protein abundance. Mean ±SD are shown (n=4). *F*, qPCR for expression of RPA194 transcript following treatment of A375 cells with the indicated drugs for 4 hours. C, ctrl; B, BMH-21. Data are represented as mean ± SD of n=3 biological replicates. *G*, qPCR analysis for verification of knockdown of SKP1, CUL9, FBXL14, and RBX1 in A375 cells using two independent siRNAs against each target (n=1).

H

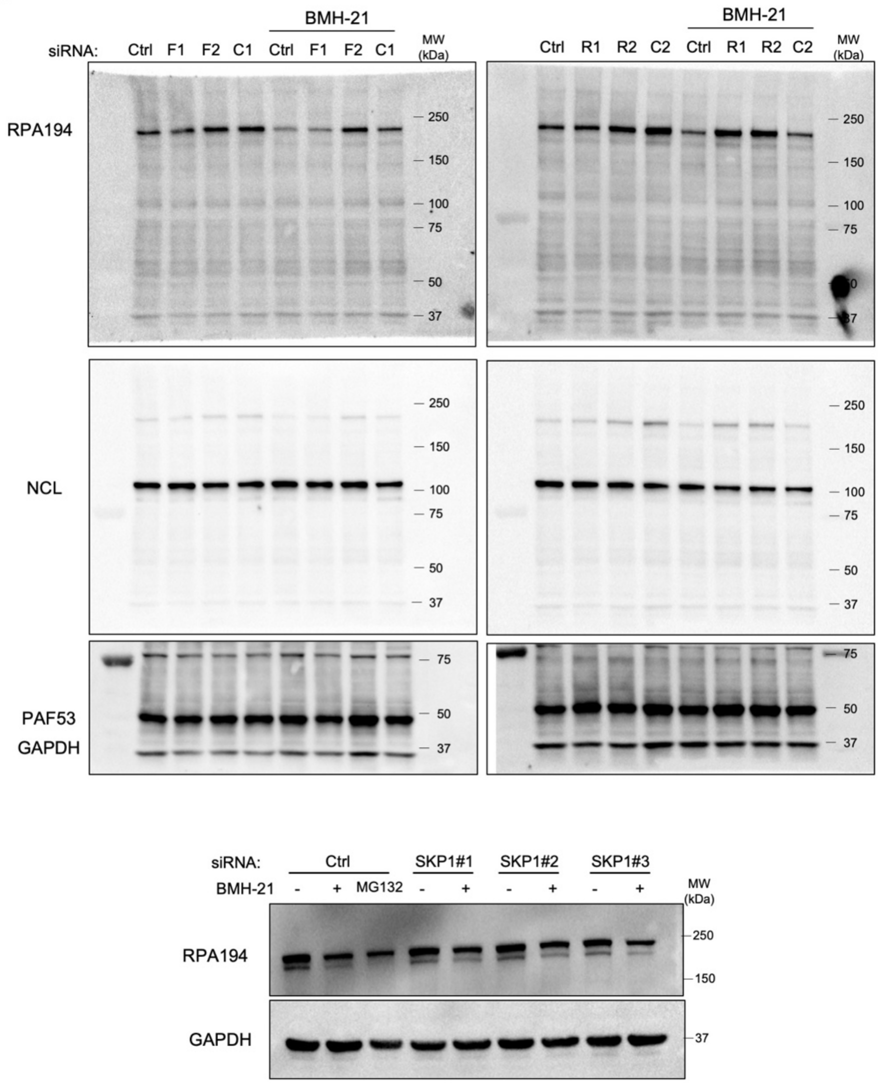

(S1 continued) H, western blotting analysis for RPA194 following transfection with siRNAs against the SCF complex genes (2-3 siRNAs against each). Representative experiments of n=3 biological experiments are shown. SiRNA control (Ctrl), FBXL14 (F), CUL9 (C), RBX1 (R).

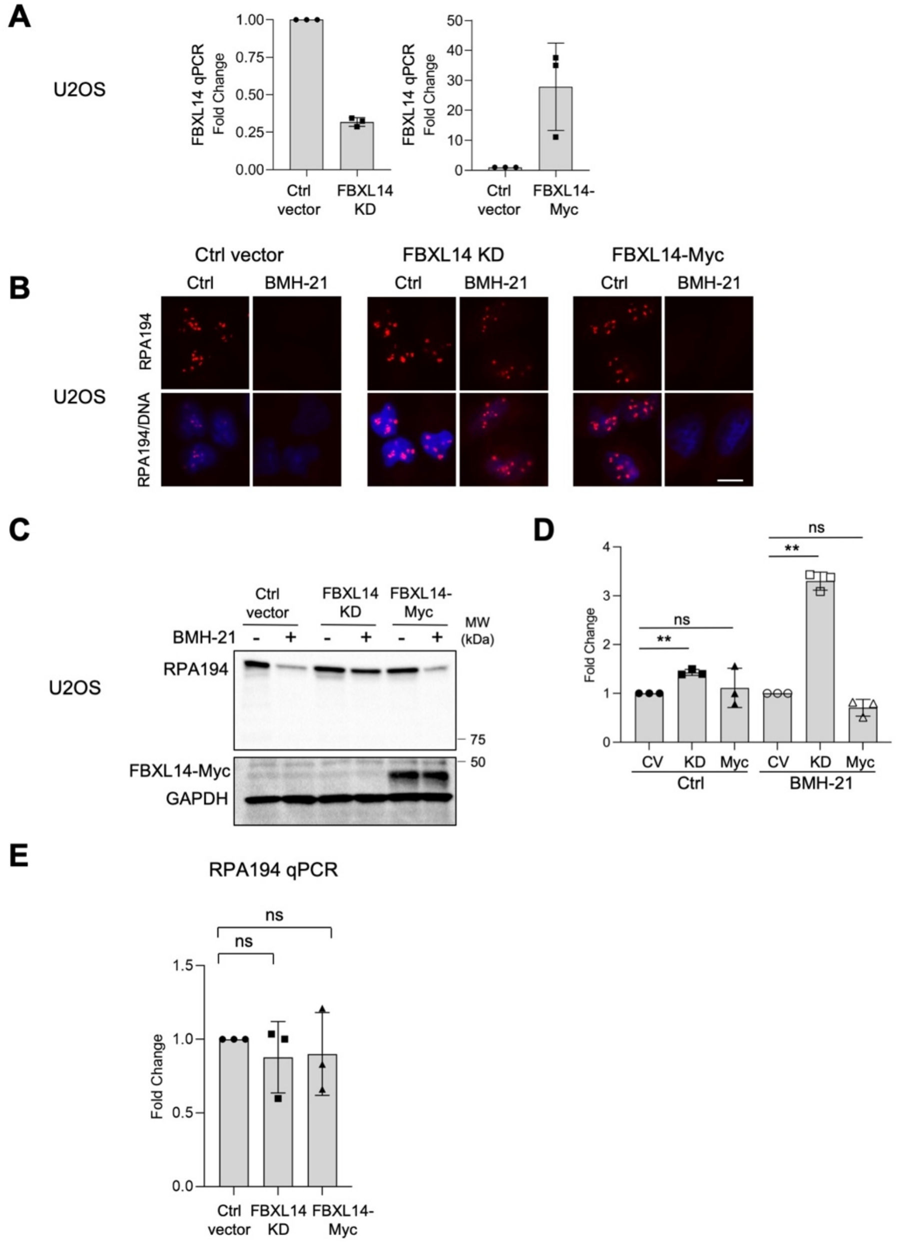

Figure S2. Inducible degradation of RPA194 depends on FBXL14. *A*, qPCR analysis of FBXL14 transcript in U2OS osteosarcoma cells with stable FBXL14 knockdown (KD) (*left*) or FBXL14-Myc overexpression (Myc) (*right*). Data are represented as mean ± SD of n=3 biological replicates. *B-D*, U2OS cells were treated with or without BMH-21 (1 µM) for 4 hours. *B*, Immunofluorescence analysis (n=3) of RPA194. Scale bar, 10 µm. *C and D*, Western blot analysis and quantification (n=3). The samples were normalized to the Ctrl vector (CV) expressing cells with or without BMH-21 treatment set as 1 and are represented as mean ± SD. *P*, one sample t test. *E*, qPCR analysis of the RPA194 transcript in A375 cells modified for FBXL14 expression. Data are represented as mean ± SD of n=3 biological replicates. Statistical significance was determined using one-way ANOVA and Tukey's post hoc test.

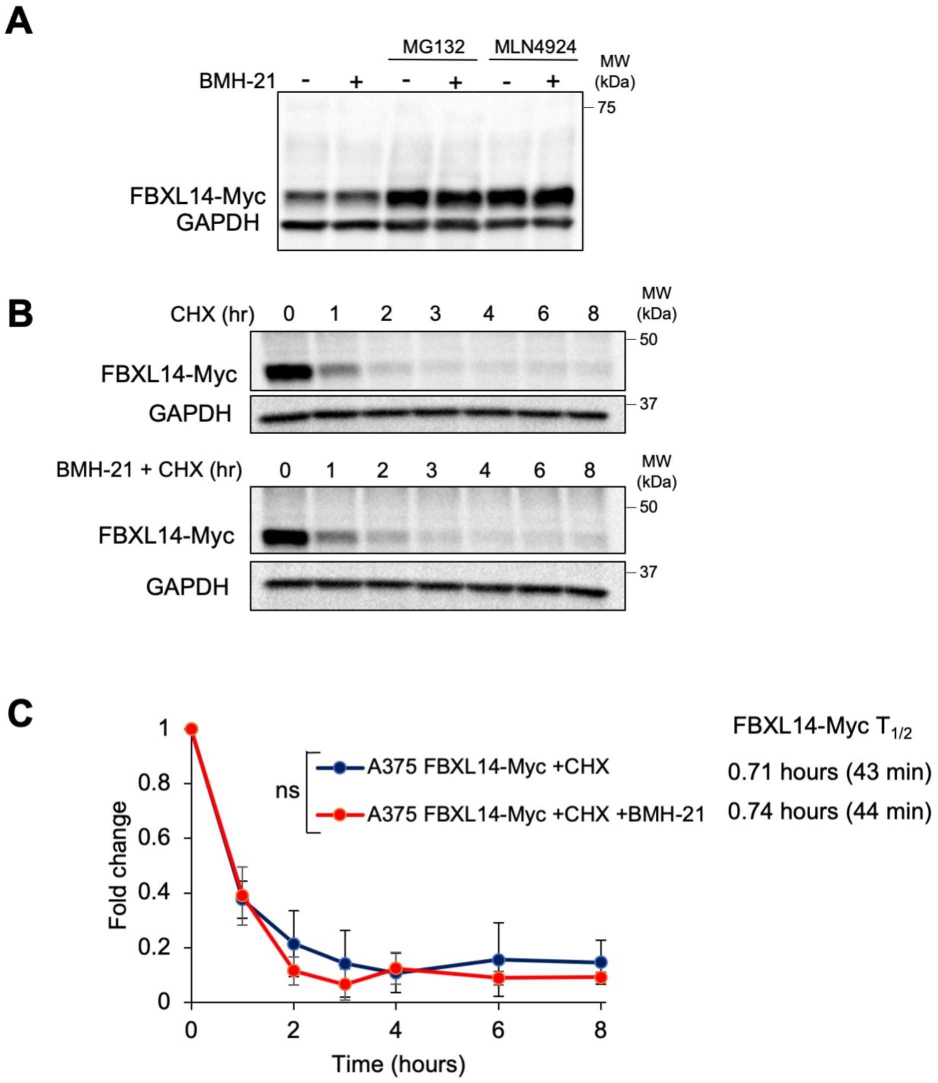

Figure S3. BMH-21 does not affect the half-life of FBXL14. *A*, A375 cells expressing FBXL14-Myc were treated with BMH-21 (1 μM), MG132 (10 μM) or MLN4924 (1 μM) as indicated for 4 hours, and cell lysates were analyzed by immunoblotting (n=3). *B*, A375 cells expressing FBXL14-Myc were treated with CHX (10 μg/mL) in the presence or absence of BMH-21 (1 μM), and cell lysates were collected at the indicated times. *C*, Quantification of the western blots and determination of the half-life. Data are represented as mean ± SD of n=3 biological replicates in (*B*). Statistical significance was determined using two-way ANOVA and Bonferroni's post hoc test.

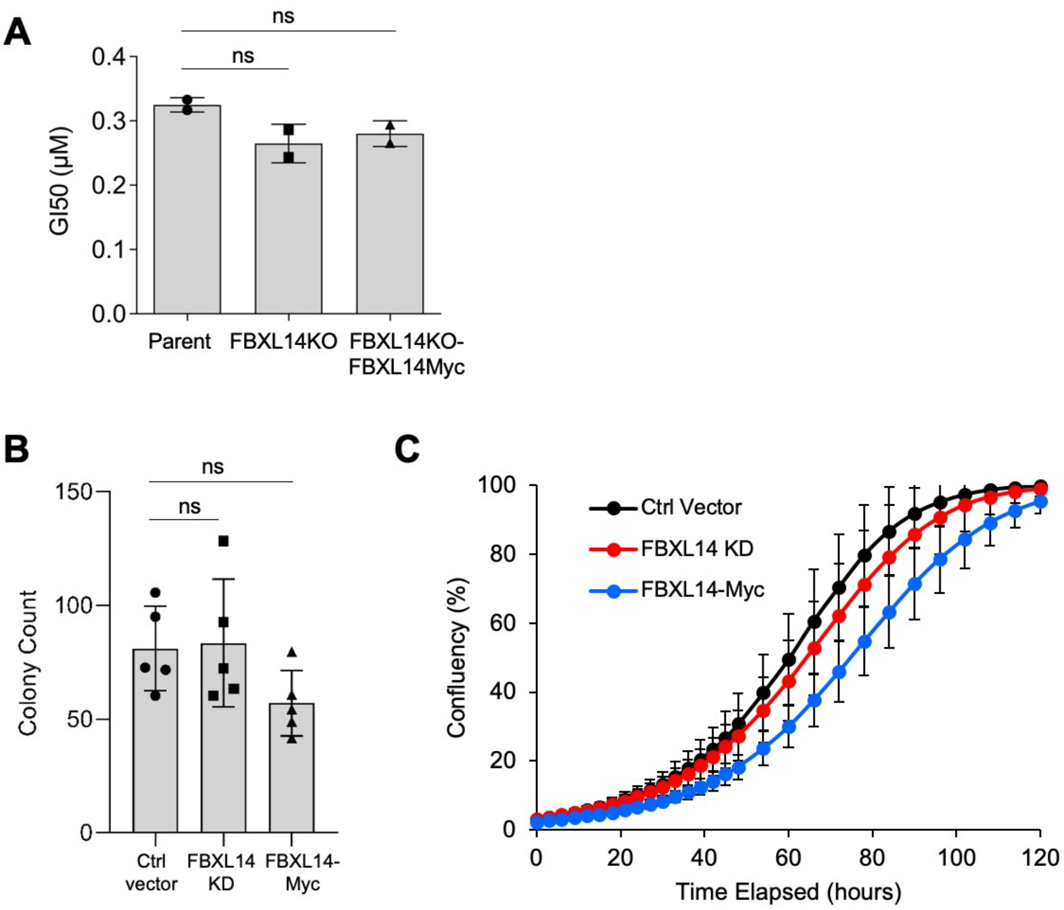

Figure S4. FBXL14 overexpression has negligible effect on cell growth. *A*, HAP1 parent, FBXL14-knockout (FBXL14 KO), and FBXL14 KO cells reconstituted with FBXL14-Myc were treated with half-log concentrations of BMH-21 for three days, and cell viability was determined. GI_{50} was determined of n=2 biological experiments. Data are represented as mean ± SD. Statistical significance was determined using one-way ANOVA and Tukey's post hoc test. *B*, A375 Ctrl vector, FBXL14 KD, and FBXL14-Myc cells were incubated for 7 days, fixed, and stained. The mean number of colonies of n=5 biological experiments were counted. Data are represented as mean ± SD. Statistical significance was determined using one-way ANOVA and Tukey's post hoc test. *C*, Live cell measurements of A375 Ctrl vector, FBXL14 KD, and FBXL14-Myc cells were obtained over 5 days. The mean ± SD of n=3 biological replicates is shown.

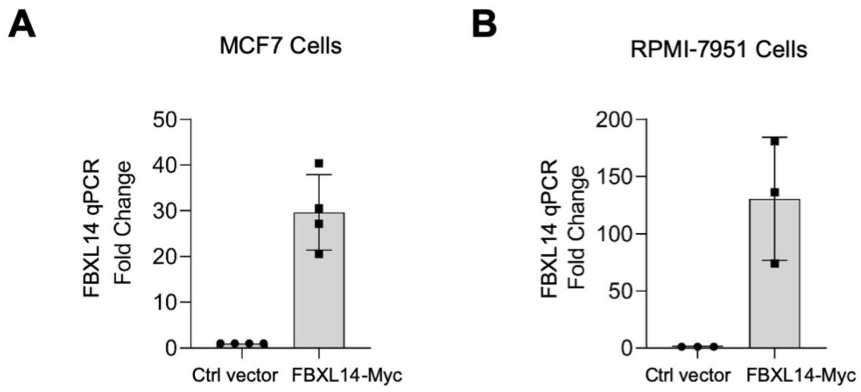

Figure S5. Generation of MCF7 and RPMI-7951 cells stably overexpressing FBXL14-Myc. *A, B*, qPCR analysis of FBXL14 expression. *A,* MCF7 breast adenocarcinoma cells and *B,* RPMI-7951 melanoma cells. Data are represented as mean ± SD of n=3 biological replicates. *P*, unpaired t-tests.

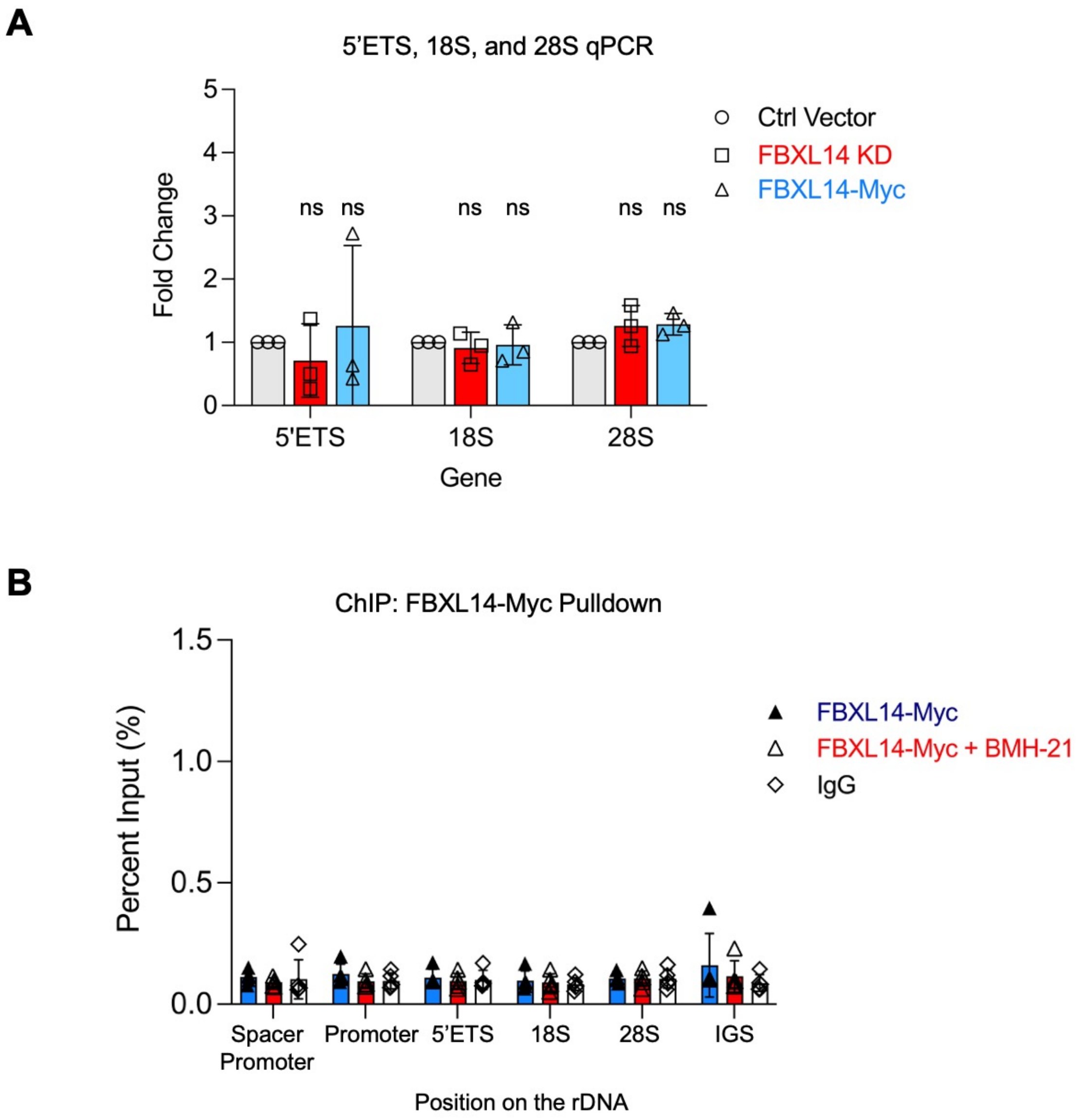

Figure S6. FBXL14 expression does not affect rRNA transcription. *A*, qPCR analysis of 5'ETS, 18S, and 28S rRNA. Data are represented as mean ± SD of n=3 biological replicates. Statistical significance was determined using one-way ANOVA and Tukey's post hoc test. *B*, ChIP was conducted using Myc-tag pulldowns for FBXL14-Myc or IgG on A375 FBXL14-Myc cells. Cells were treated with a vehicle control (DMSO) or BMH-21 (1 μM) for 30 minutes. Primers for the rDNA promoters and gene body are indicated below. Data are represented as mean ± SD of n=5 biological replicates.
