# Supplementary figures and images for: Identification of an E3 ligase that targets the catalytic subunit of RNA Polymerase I upon transcription stress
Source: J Biol Chem. 2022 Nov 11;298(12):102690. doi: 10.1016/j.jbc.2022.102690 (PMC9727647; doi:10.1016/j.jbc.2022.102690)

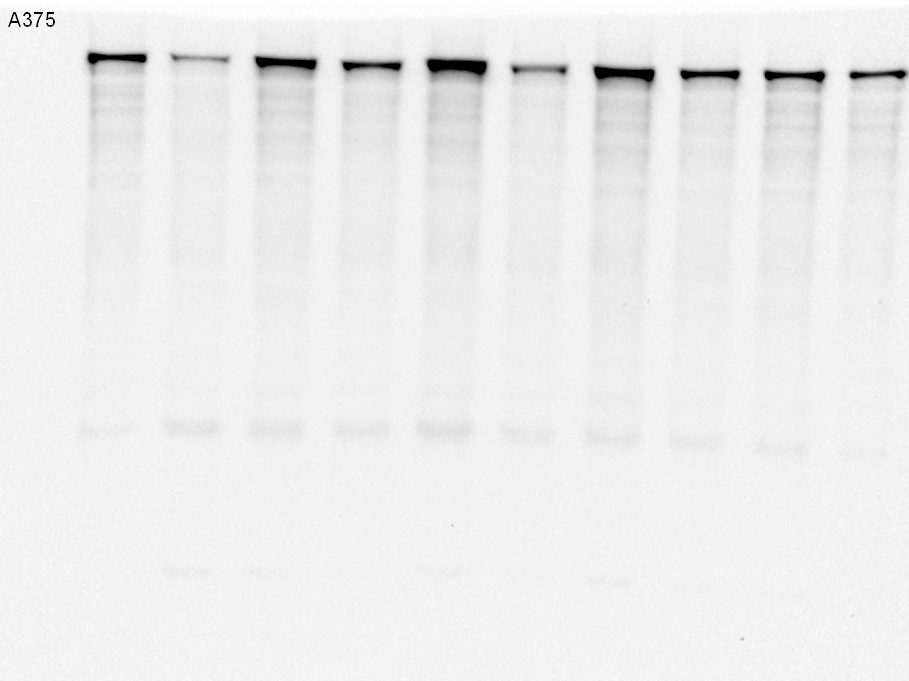

Supplement: Uncropped gels [file mmc3.zip › Uncropped gels/Fig1/1G/Hester Liu 2018-06-01 12hr 54min_Exposure_40.0secA375.jpg]

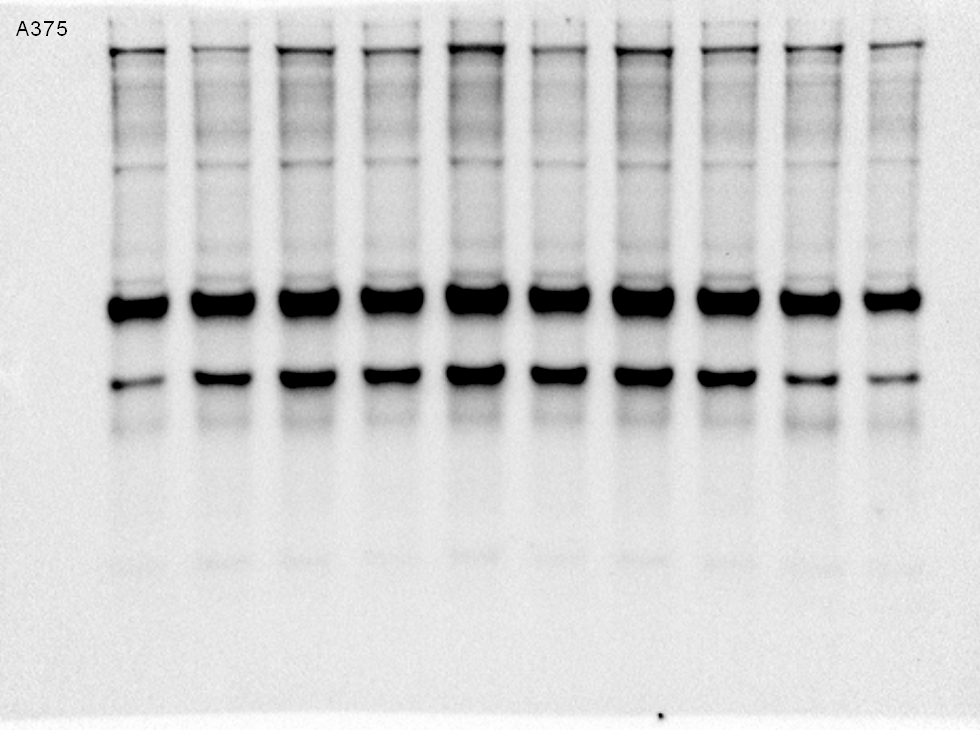

Supplement: Uncropped gels [file mmc3.zip › Uncropped gels/Fig1/1G/Hester Liu 2018-06-06 13hr 57min_Exposure_20.0secA375_GAPDH.jpg]

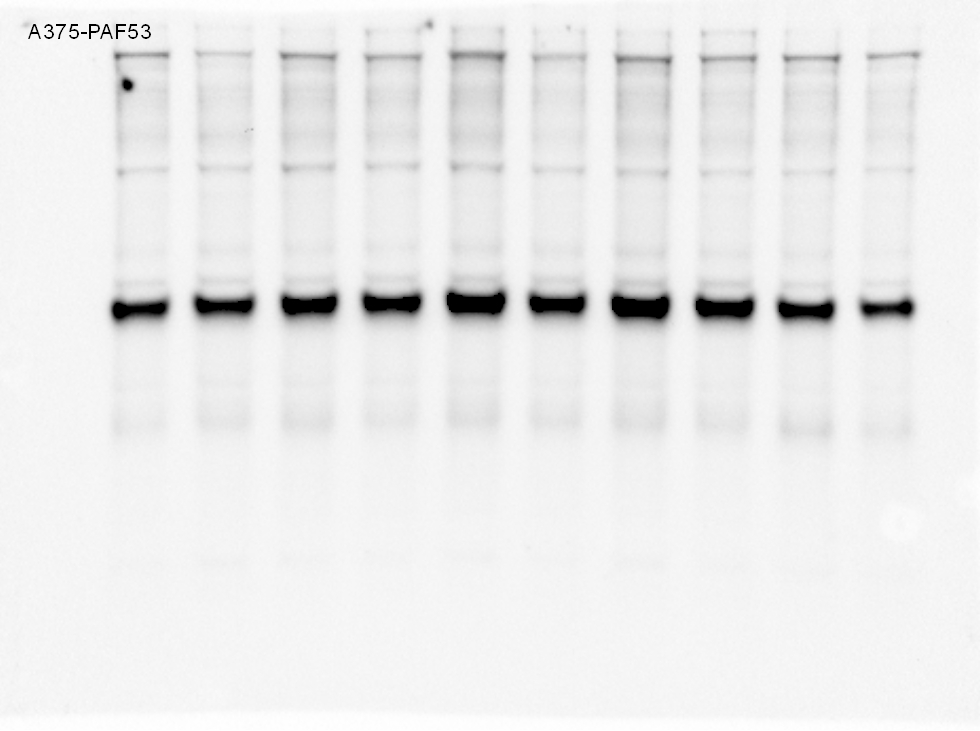

Supplement: Uncropped gels [file mmc3.zip › Uncropped gels/Fig1/1G/Hester Liu 2018-06-05 14hr 01min_Exposure_20.0secA375-PAF53.jpg]

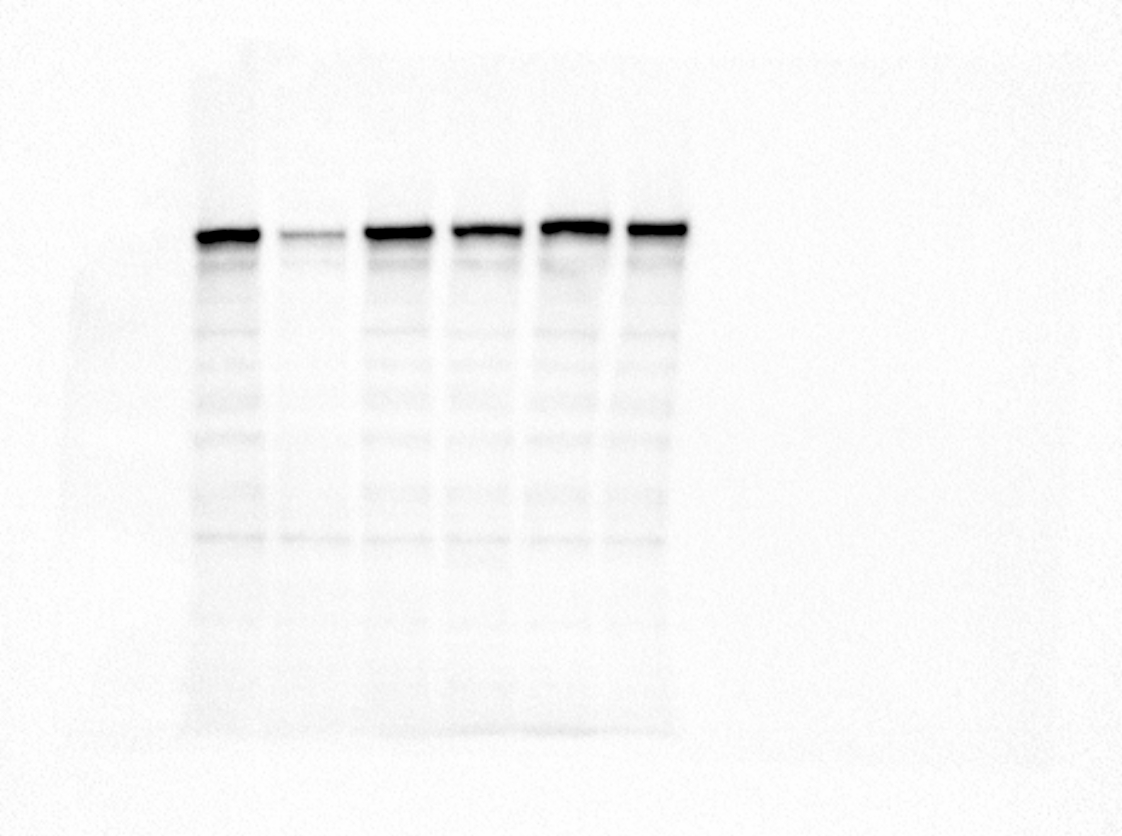

Supplement: Uncropped gels [file mmc3.zip › Uncropped gels/Fig1/1E/Steffie Pitts 2020-12-11 08hr 46min_Exposure_3.0sec A375 EV MG and MLN BR2 blotting for RPA194.jpg]

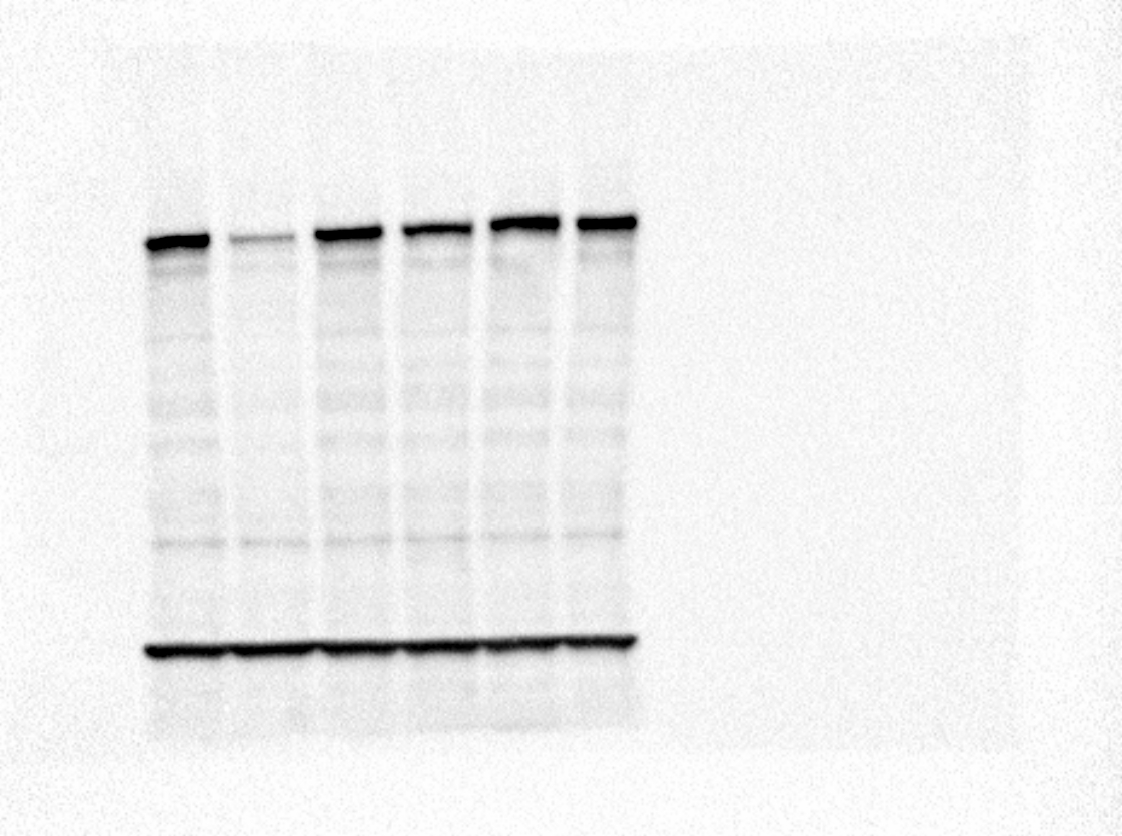

Supplement: Uncropped gels [file mmc3.zip › Uncropped gels/Fig1/1E/Steffie Pitts 2020-12-11 13hr 17min_Exposure_7.0sec A375 EV MG and MLN BR2 blotting for GAPDH.jpg]

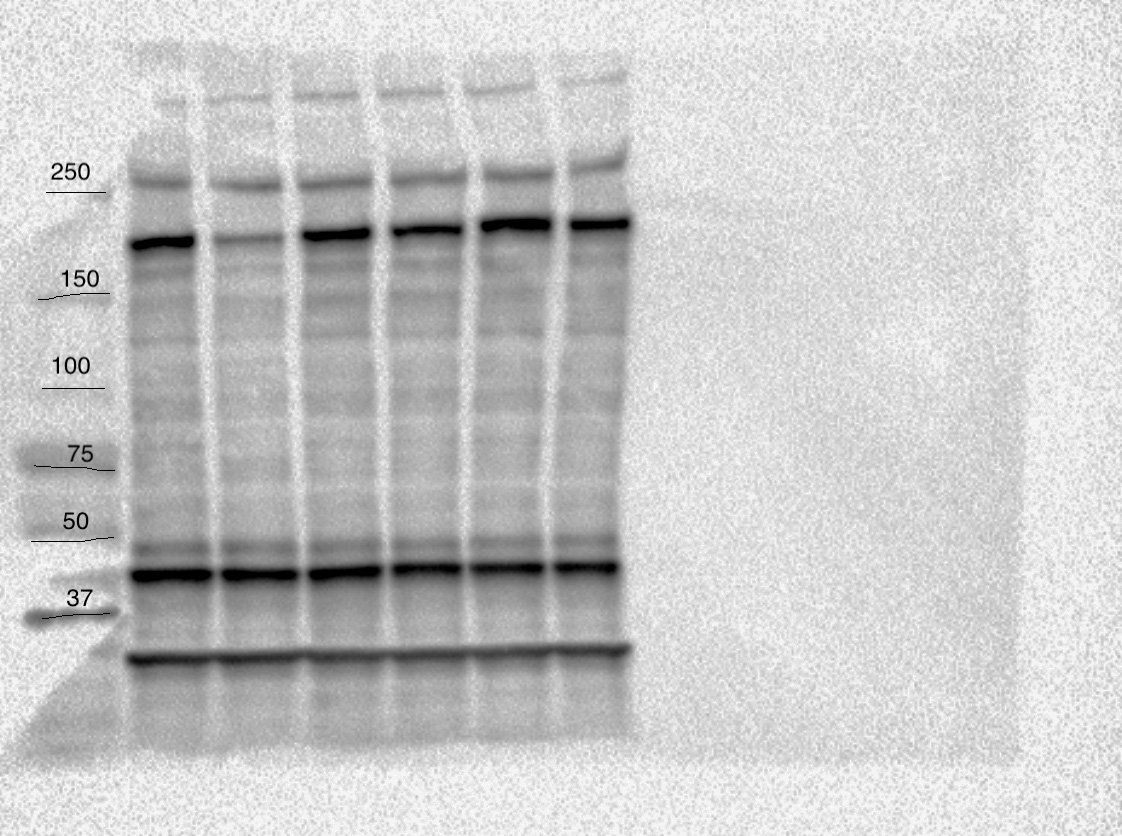

Supplement: Uncropped gels [file mmc3.zip › Uncropped gels/Fig1/1E/Steffie Pitts 2020-12-12 16hr 55min_Exposure_2.0sec A375 EV MG and MLN BR2 blotting for PAF53 edited.jpg]

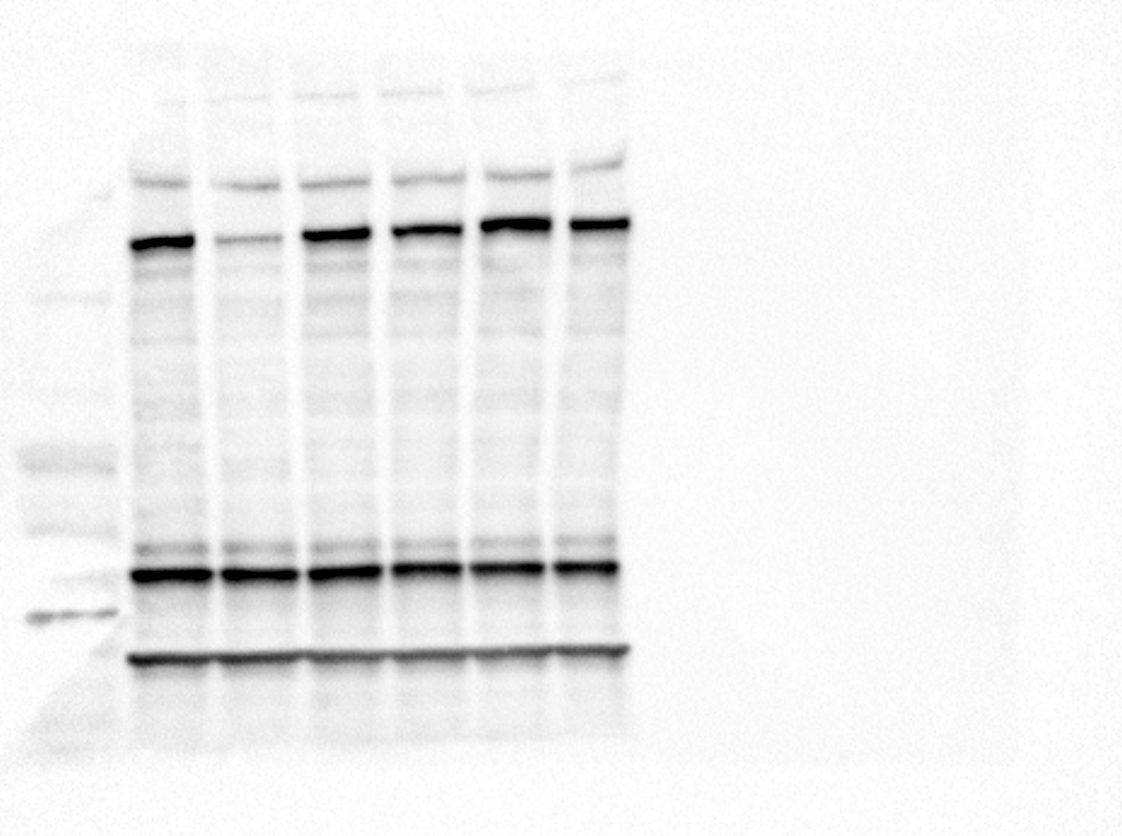

Supplement: Uncropped gels [file mmc3.zip › Uncropped gels/Fig1/1E/Steffie Pitts 2020-12-12 16hr 55min_Exposure_2.0sec A375 EV MG and MLN BR2 blotting for PAF53.jpg]

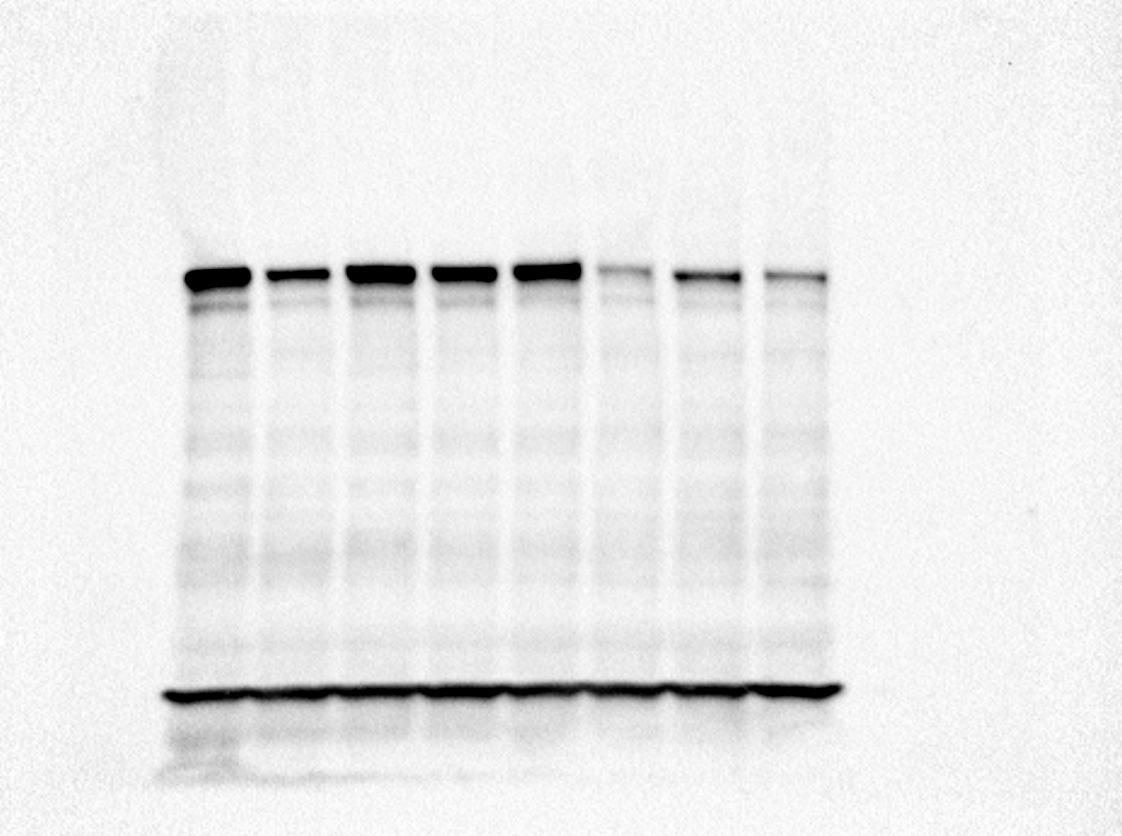

Supplement: Uncropped gels [file mmc3.zip › Uncropped gels/Fig7/7A/Steffie Pitts 2021-09-23_18h02m03s_Exposure_6.0sec A375 EV and FBXL14-Myc with various drugs BR1 TR2 blotting for GAPDH.jpg]

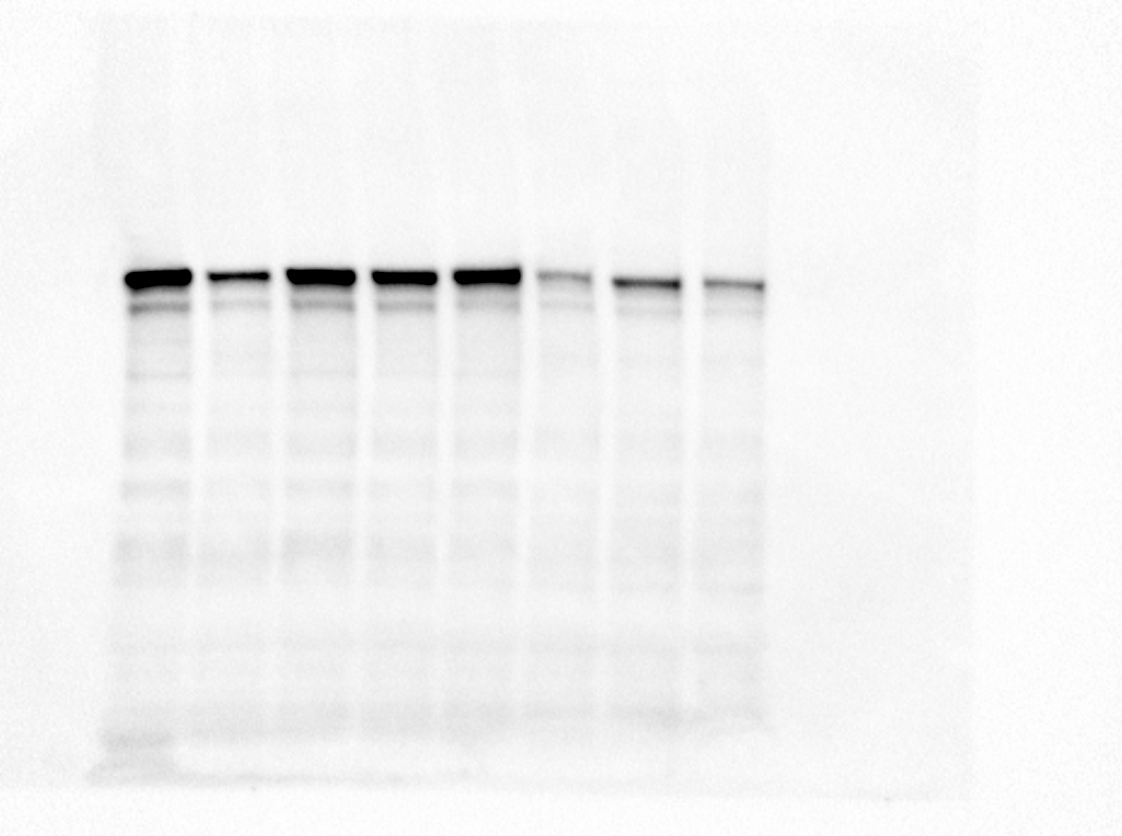

Supplement: Uncropped gels [file mmc3.zip › Uncropped gels/Fig7/7A/Steffie Pitts 2021-09-22_18h55m09s_Exposure_4.0sec A375 EV and FBXL14-Myc with various drug treatments BR1 TR2.jpg]

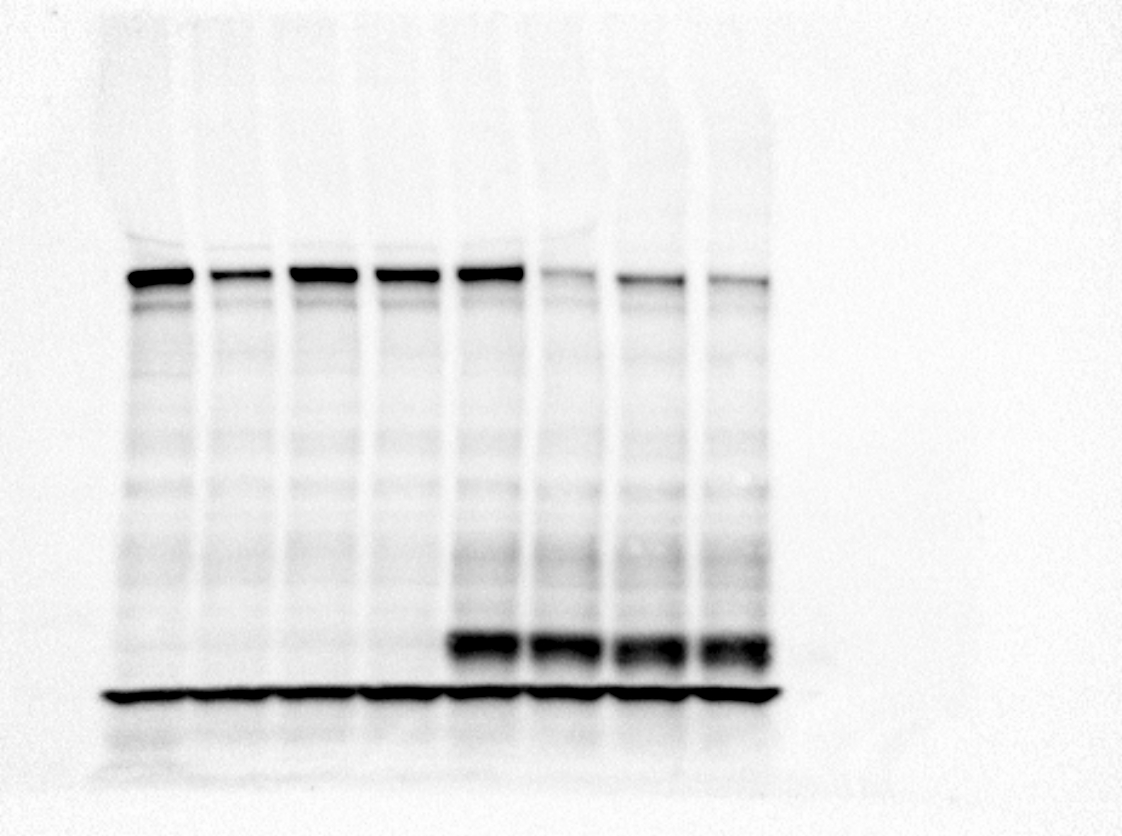

Supplement: Uncropped gels [file mmc3.zip › Uncropped gels/Fig7/7A/Steffie Pitts 2021-09-24_08h41m52s_Exposure_3.0sec A375 EV and FBXL14-Myc with various drugs BR1 TR2 blotting for Myc 4A6.jpg]

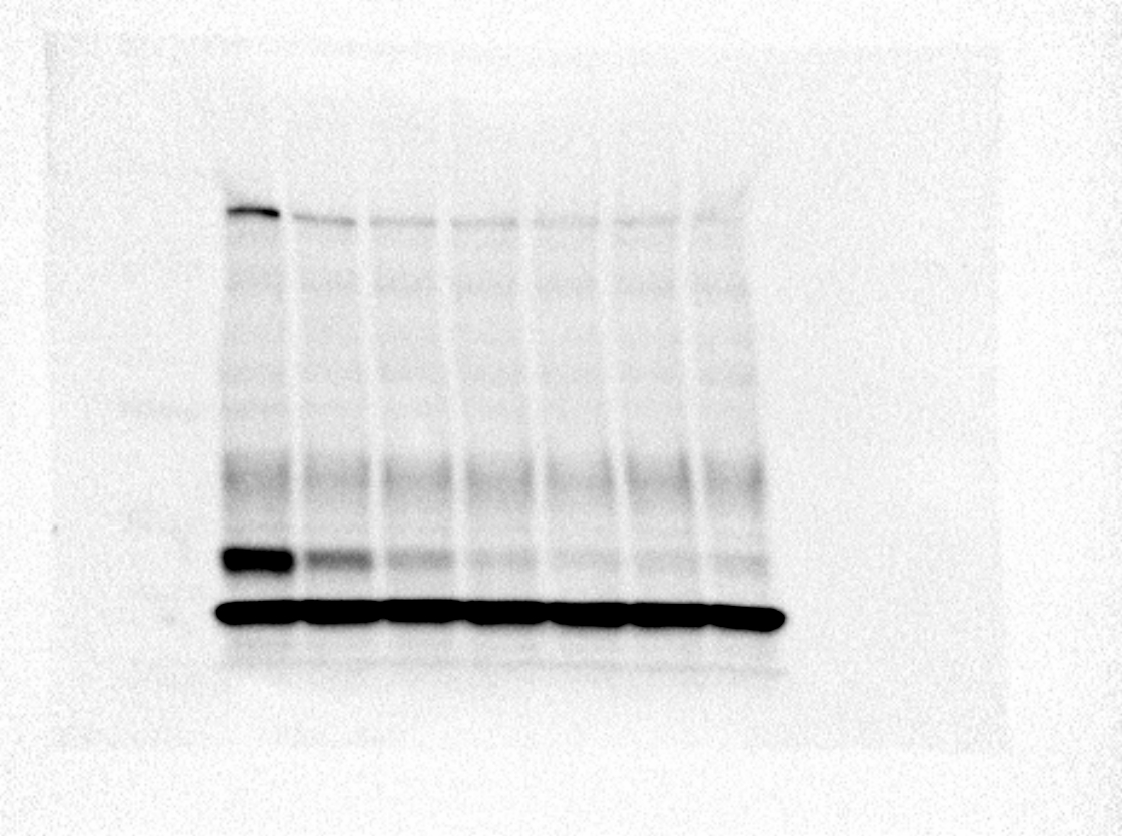

Supplement: Uncropped gels [file mmc3.zip › Uncropped gels/FigS3/S3B/Steffie Pitts 2020-08-27 12hr 49min_Exposure_7.0sec FBX CHX + BMH blotting for Myc.jpg]

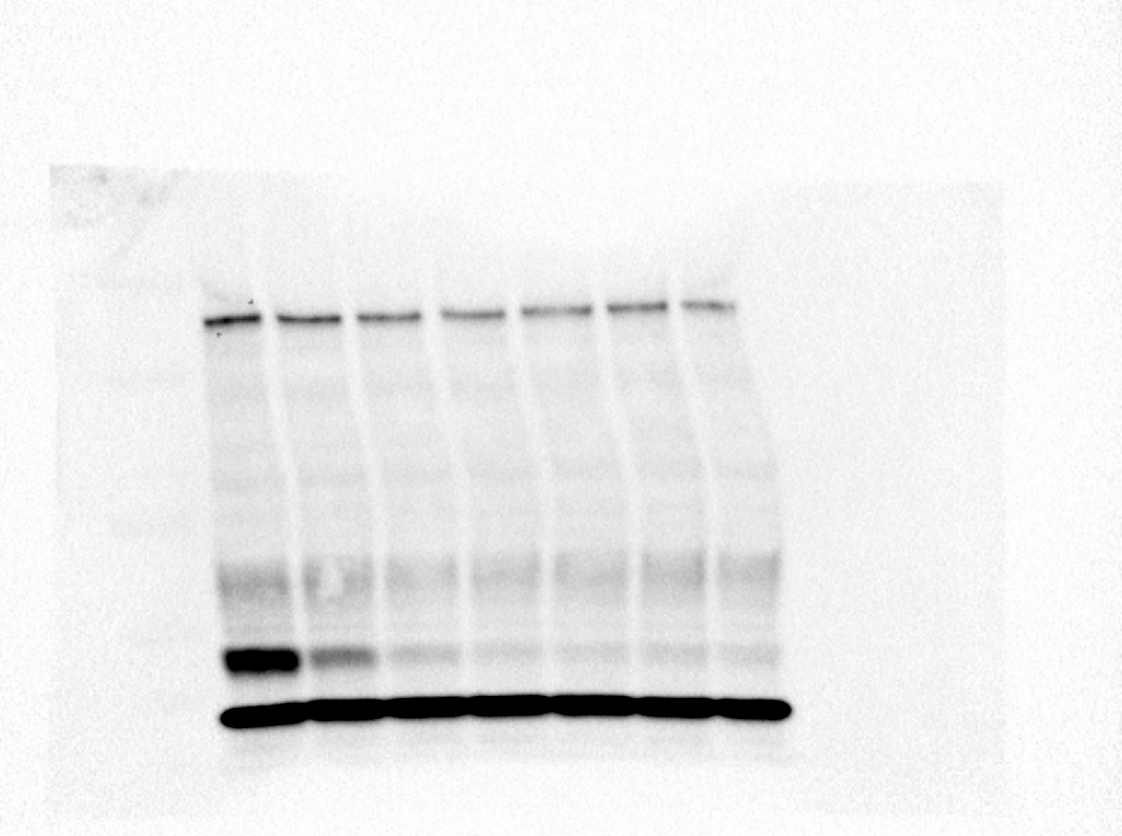

Supplement: Uncropped gels [file mmc3.zip › Uncropped gels/FigS3/S3B/Steffie Pitts 2020-08-27 12hr 45min_Exposure_7.0sec FBX CHX blotting for Myc.jpg]

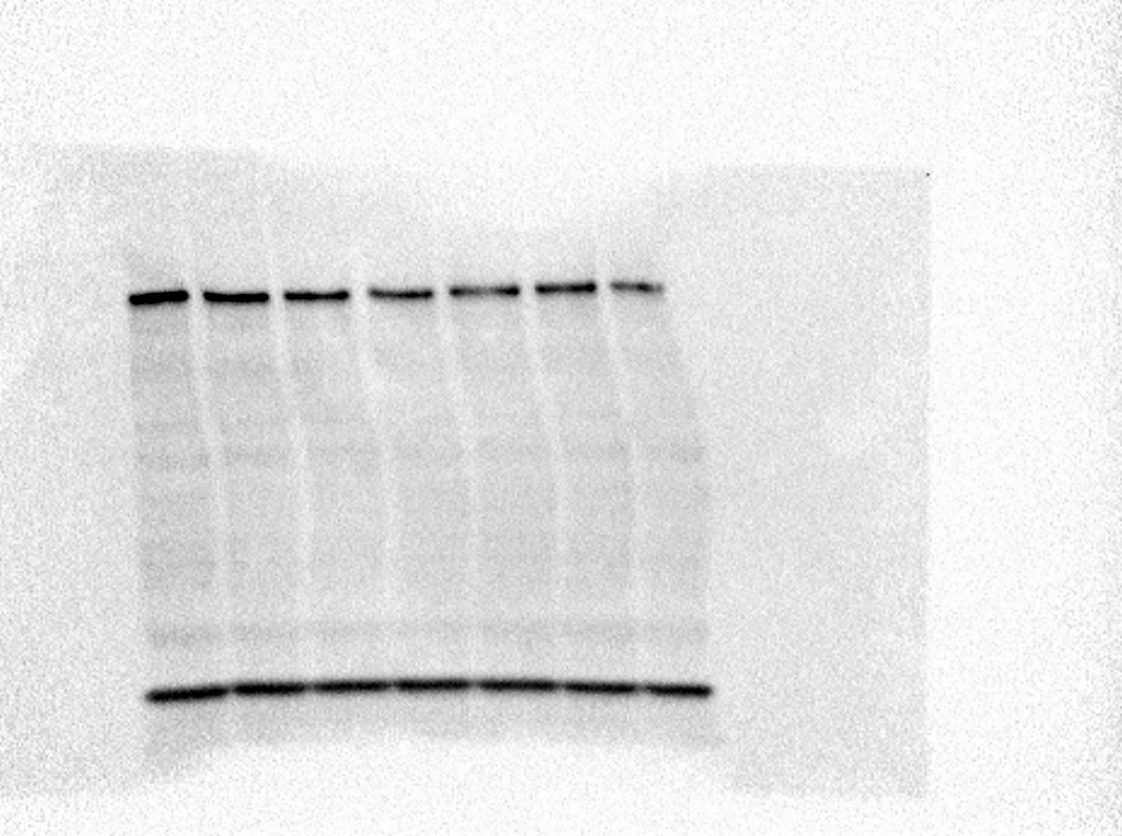

Supplement: Uncropped gels [file mmc3.zip › Uncropped gels/FigS3/S3B/Steffie Pitts 2020-08-26 17hr 13min_Exposure_15.0sec FBX CHX blotting for GAPDH.jpg]

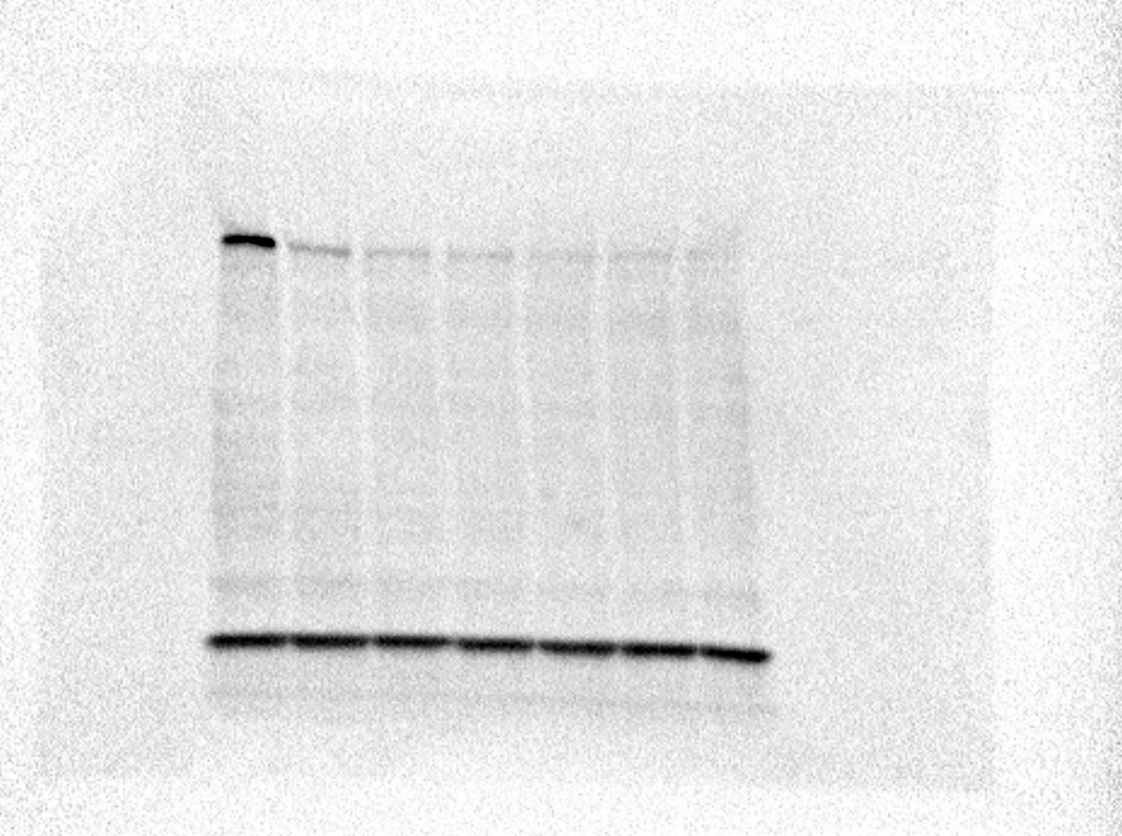

Supplement: Uncropped gels [file mmc3.zip › Uncropped gels/FigS3/S3B/Steffie Pitts 2020-08-26 17hr 16min_Exposure_15.0sec FBX CHX + BMH blotting for GAPDH.jpg]

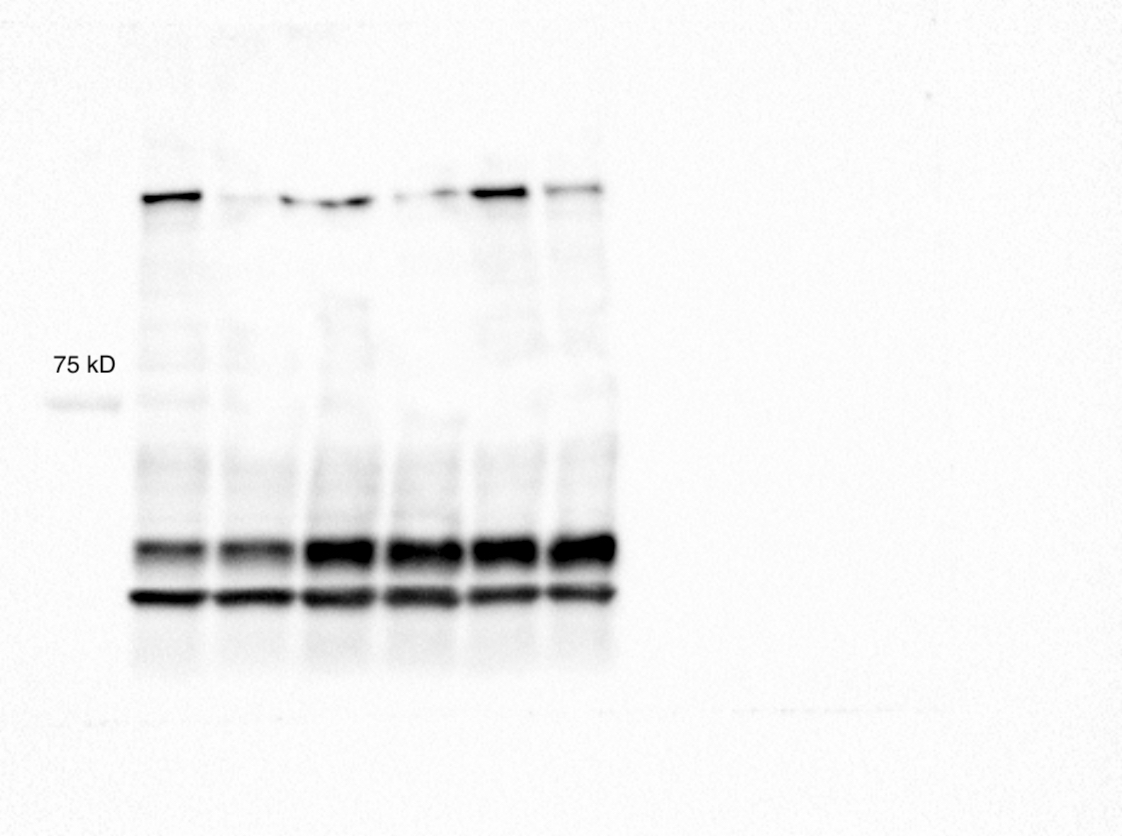

Supplement: Uncropped gels [file mmc3.zip › Uncropped gels/FigS3/S3A/Steffie Pitts 2021-07-22_15h05m17s_Exposure_2.0sec FBXL14-Myc Repeats BR3 blotting for Myc 4A6 image 2.jpg]

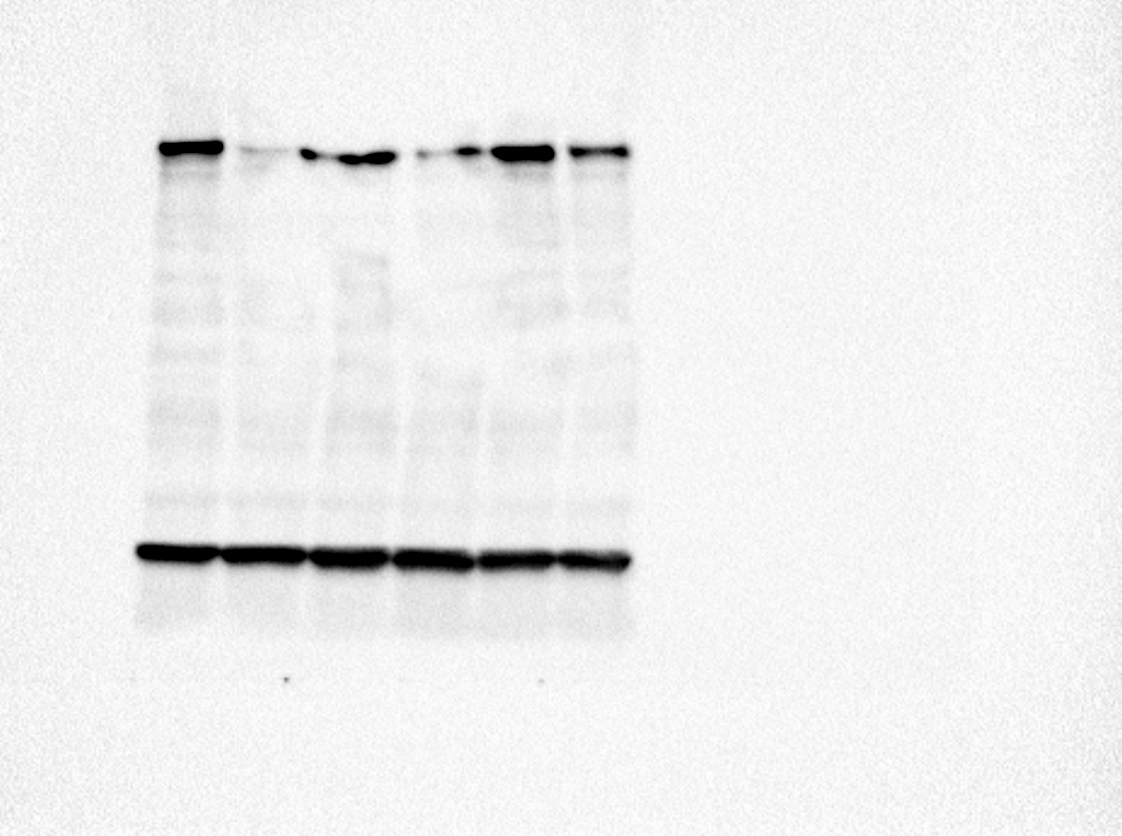

Supplement: Uncropped gels [file mmc3.zip › Uncropped gels/FigS3/S3A/Steffie Pitts 2021-07-21_21h59m41s_Exposure_7.0sec FBXL14-Myc BR3 blotting for GAPDH.jpg]

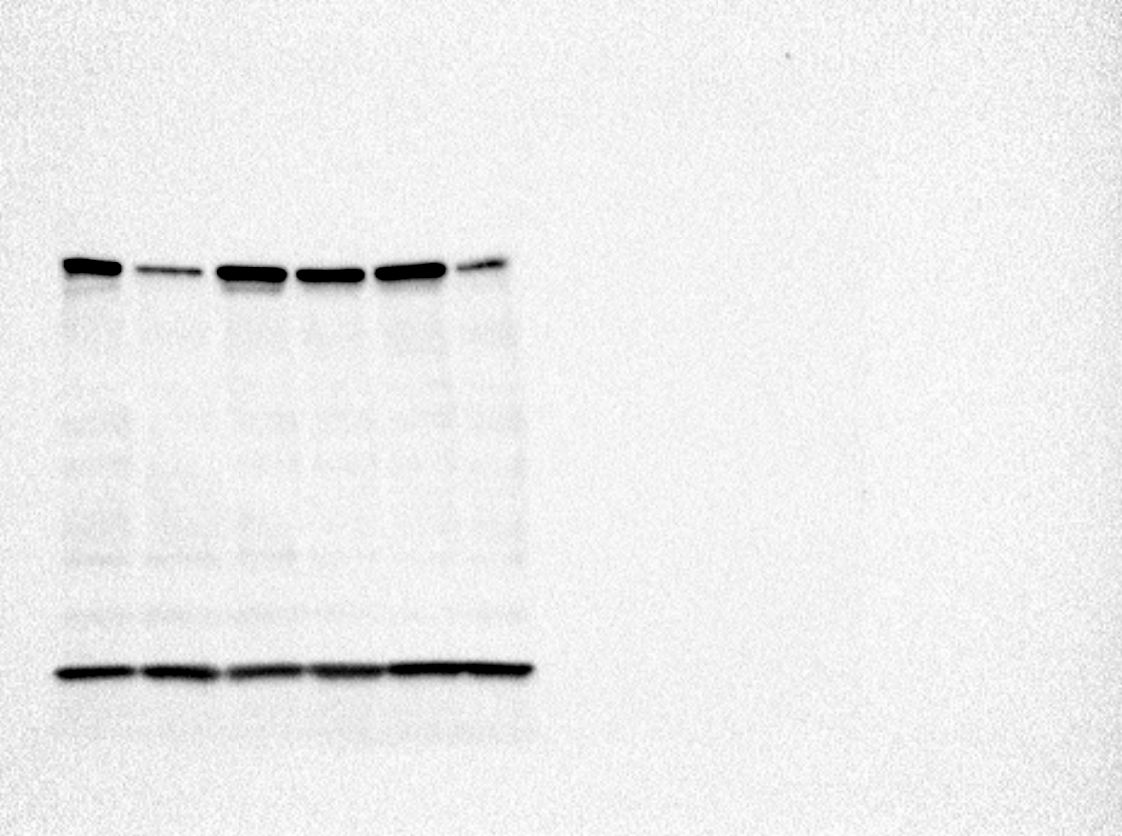

Supplement: Uncropped gels [file mmc3.zip › Uncropped gels/FigS2/S2C/Steffie Pitts 2021-08-04_19h25m59s_Exposure_9.0sec U2OS Repeats BR3 blotting for GAPDH.jpg]

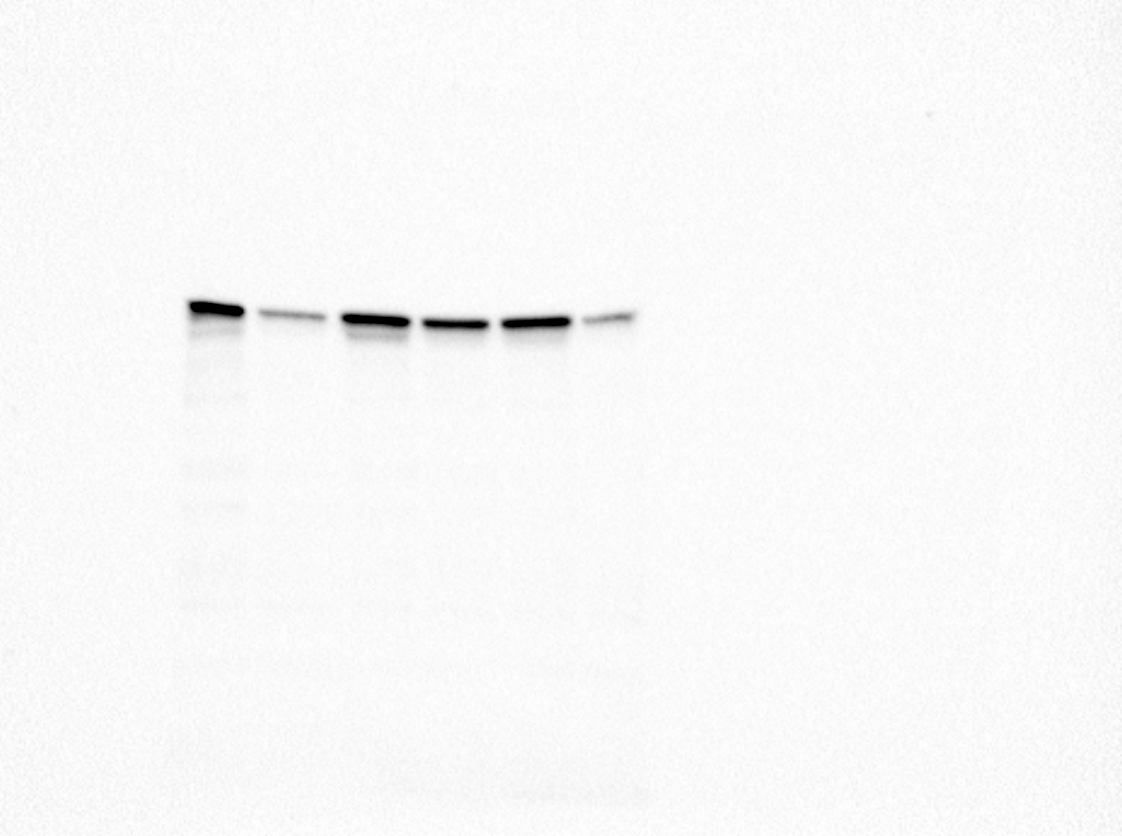

Supplement: Uncropped gels [file mmc3.zip › Uncropped gels/FigS2/S2C/Steffie Pitts 2021-08-04_16h23m28s_Exposure_5.0sec U2OS Repeats BR3 blotting for RPA194.jpg]

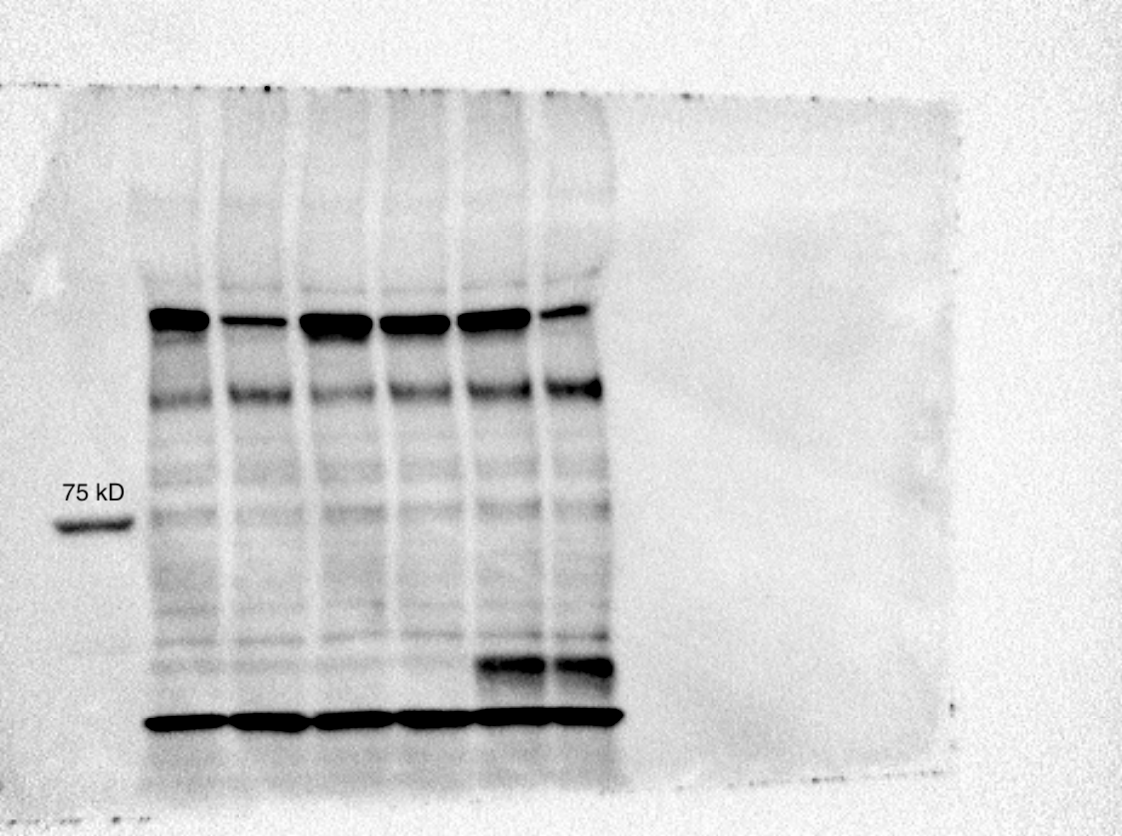

Supplement: Uncropped gels [file mmc3.zip › Uncropped gels/FigS2/S2C/Steffie Pitts 2021-08-05_12h47m56s_Exposure_8.0sec U2OS Repeats BR3 blotting for Myc 4A6.tif]

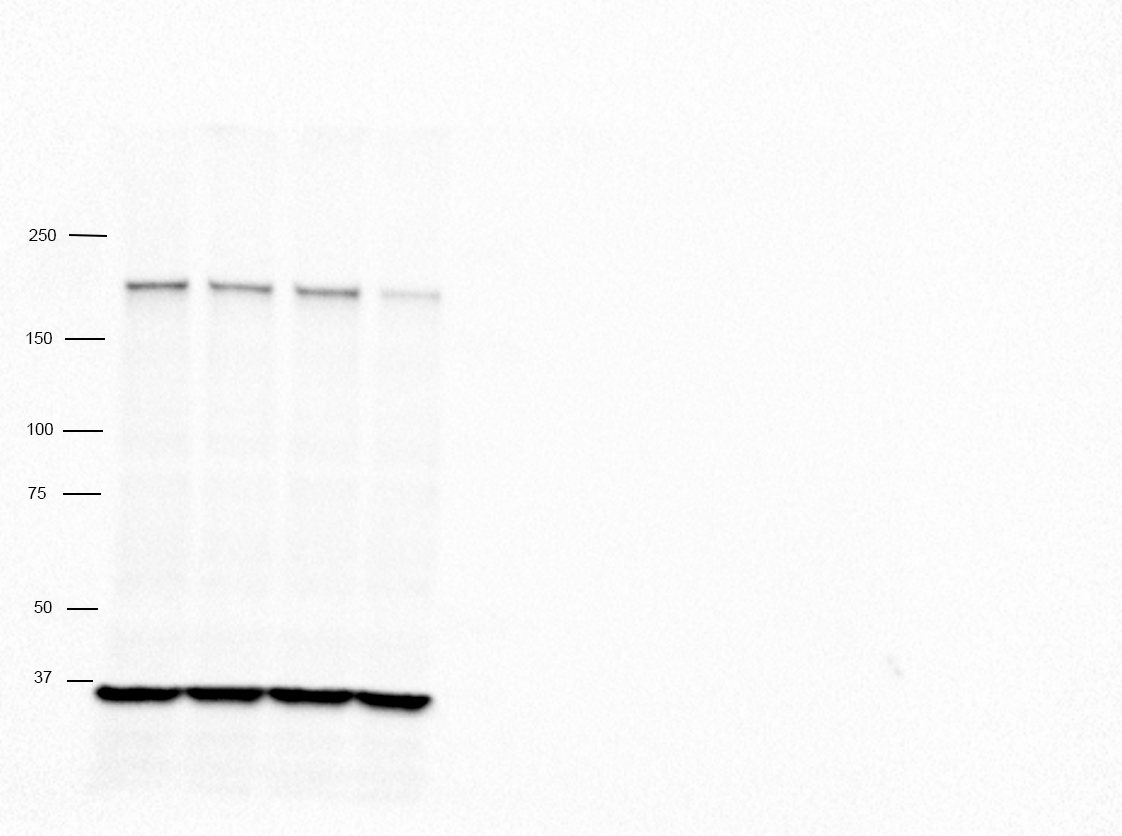

Supplement: Uncropped gels [file mmc3.zip › Uncropped gels/Fig5/5C/Steffie Pitts 2021-10-13_19h34m48s_Exposure_2.0sec MCF7 EV and FBXL14-Myc BR1 blotting for GAPDH edited.tif]

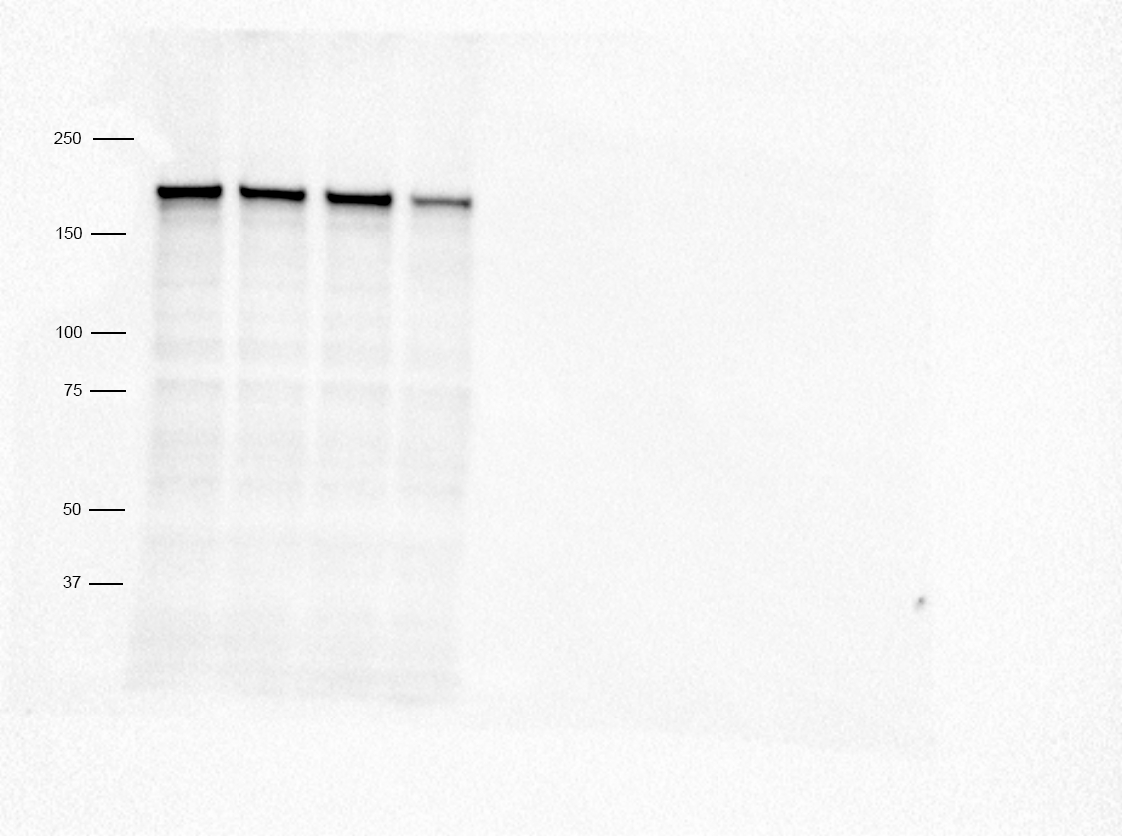

Supplement: Uncropped gels [file mmc3.zip › Uncropped gels/Fig5/5C/Steffie Pitts 2021-10-13_16h35m04s_Exposure_5.0sec MCF7 EV and FBXL14-Myc BR1 blotting for RPA194 edited.tif]

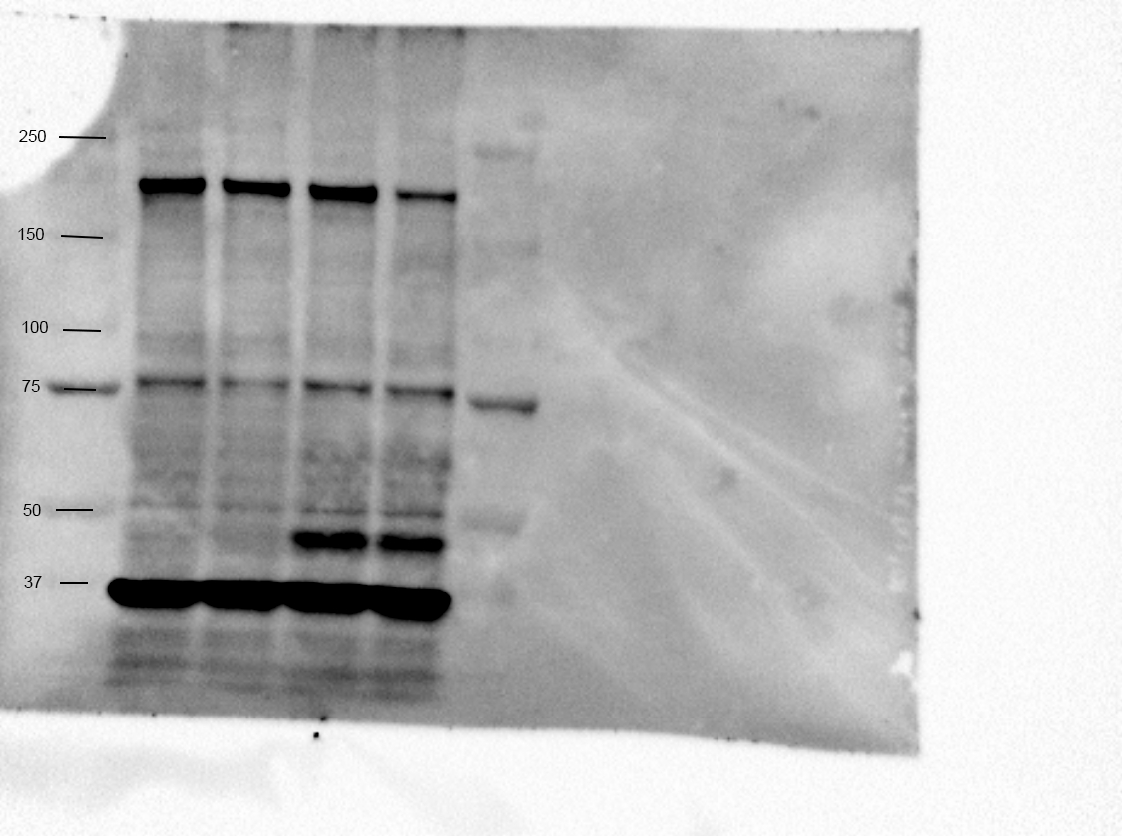

Supplement: Uncropped gels [file mmc3.zip › Uncropped gels/Fig5/5C/Steffie Pitts 2021-10-14_13h32m20s_Exposure_6.0sec MCF7 EV and FBXL14-Myc BR1 blotting for Myc 4A6 edited.tif]

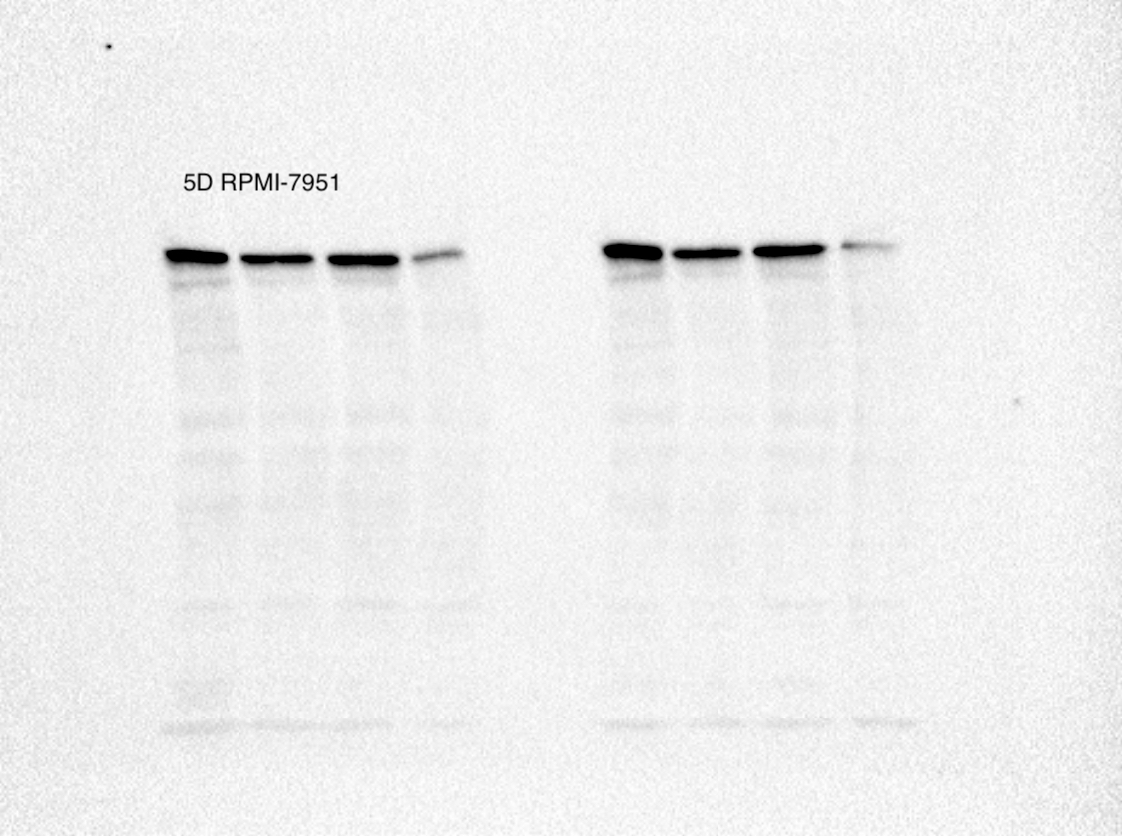

Supplement: Uncropped gels [file mmc3.zip › Uncropped gels/Fig5/5D/Steffie Pitts 2021-02-25_10h49m55s_Exposure_9.0sec RPMI-7951 BR2 and BR3 re-run blotting for RPA194.jpg]

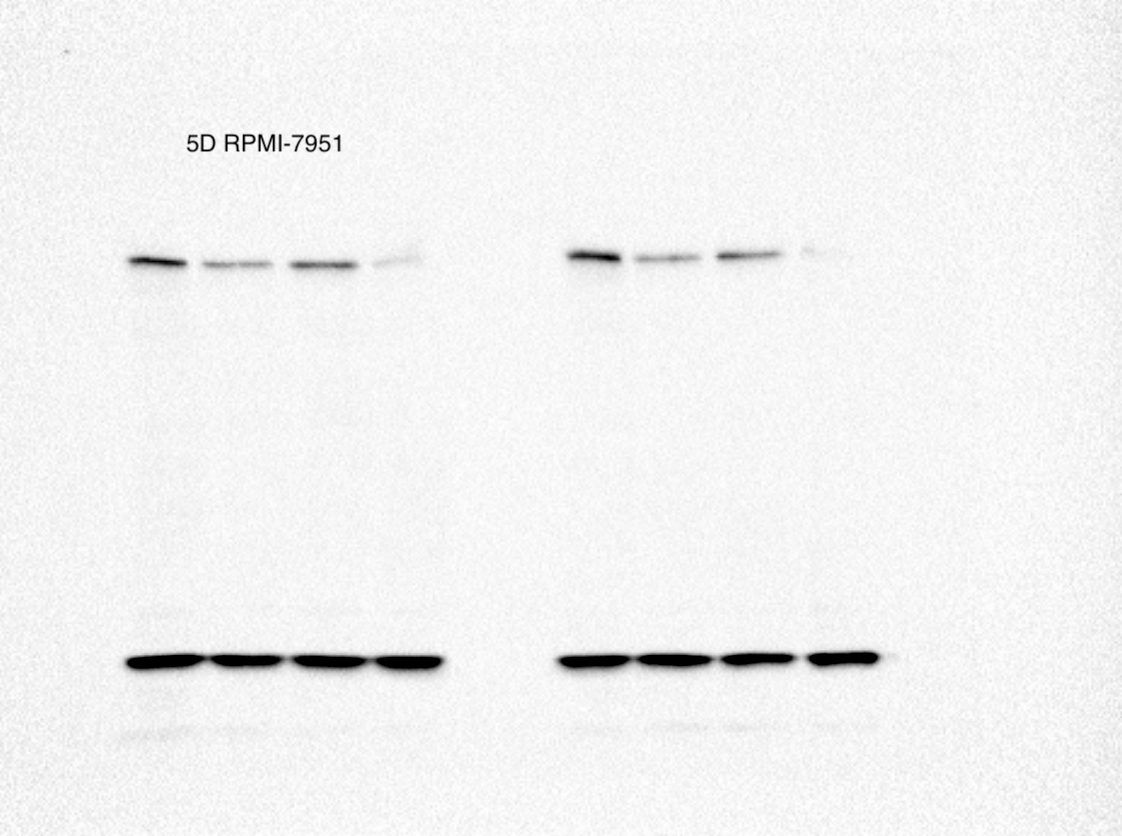

Supplement: Uncropped gels [file mmc3.zip › Uncropped gels/Fig5/5D/Steffie Pitts 2021-02-25_14h16m13s_Exposure_7.0sec RPMI-7951 BR2 and BR3 re-run blotting for GAPDH.jpg]

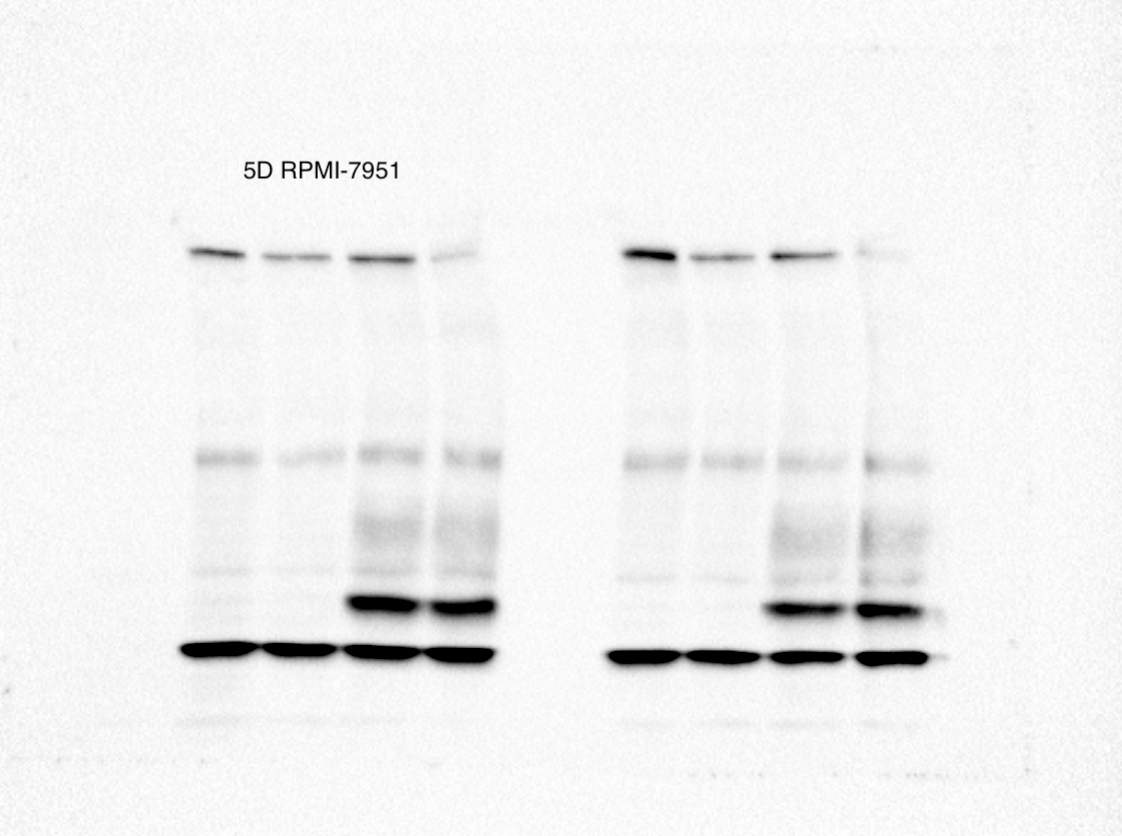

Supplement: Uncropped gels [file mmc3.zip › Uncropped gels/Fig5/5D/Steffie Pitts 2021-02-26_09h58m13s_Exposure_6.0sec RPMI-7951 BR2 and BR3 re-run blotting for Myc 4A6.jpg]

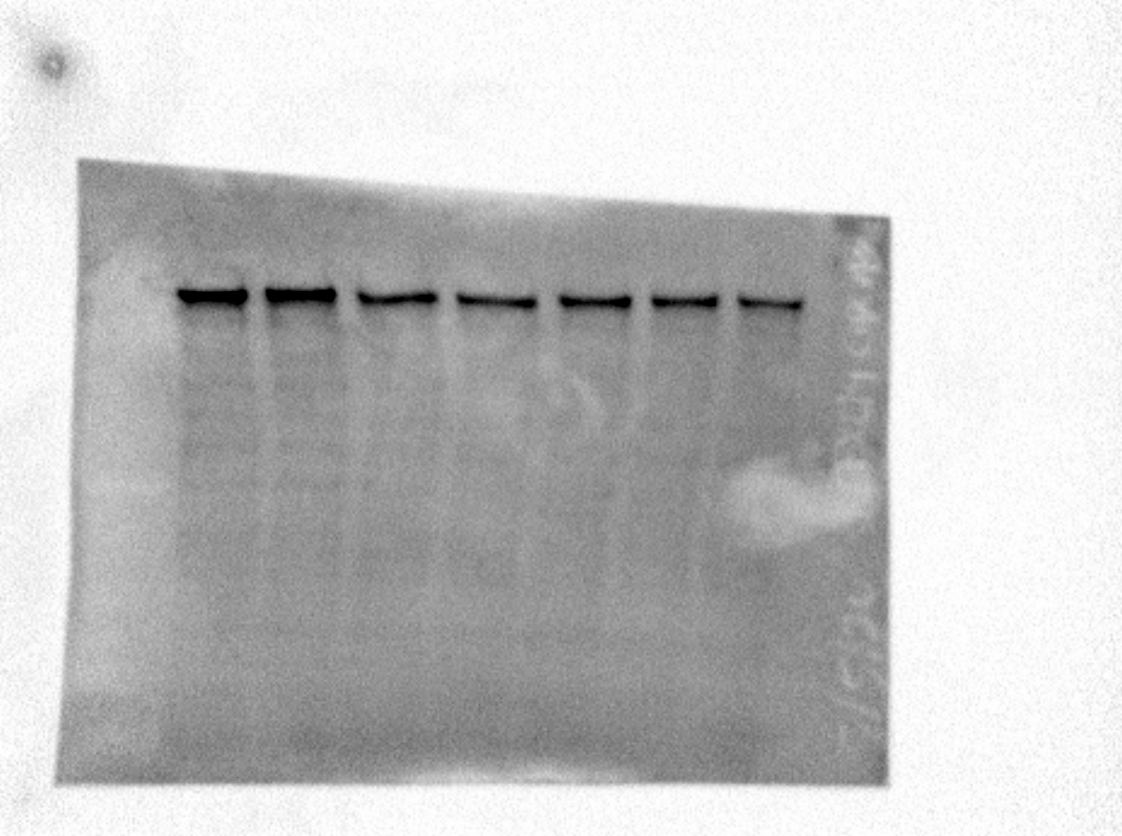

Supplement: Uncropped gels [file mmc3.zip › Uncropped gels/Fig2/2G/Steffie Pitts 2020-08-06 12hr 35min_Exposure_6.0sec shFBXL14 CHX + BMH RPA194.jpg]

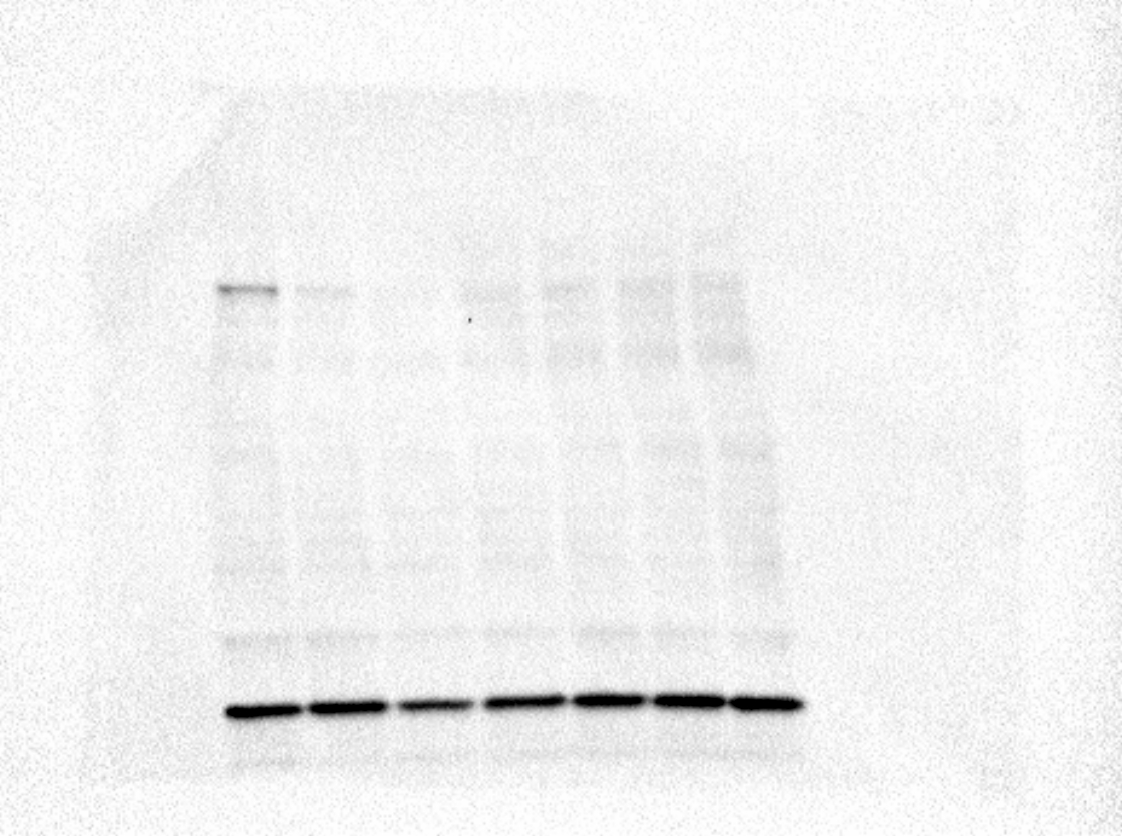

Supplement: Uncropped gels [file mmc3.zip › Uncropped gels/Fig2/2G/Steffie Pitts 2020-08-19 19hr 34min_Exposure_6.7sec FBX CHX + BMH blotting for GAPDH.jpg]

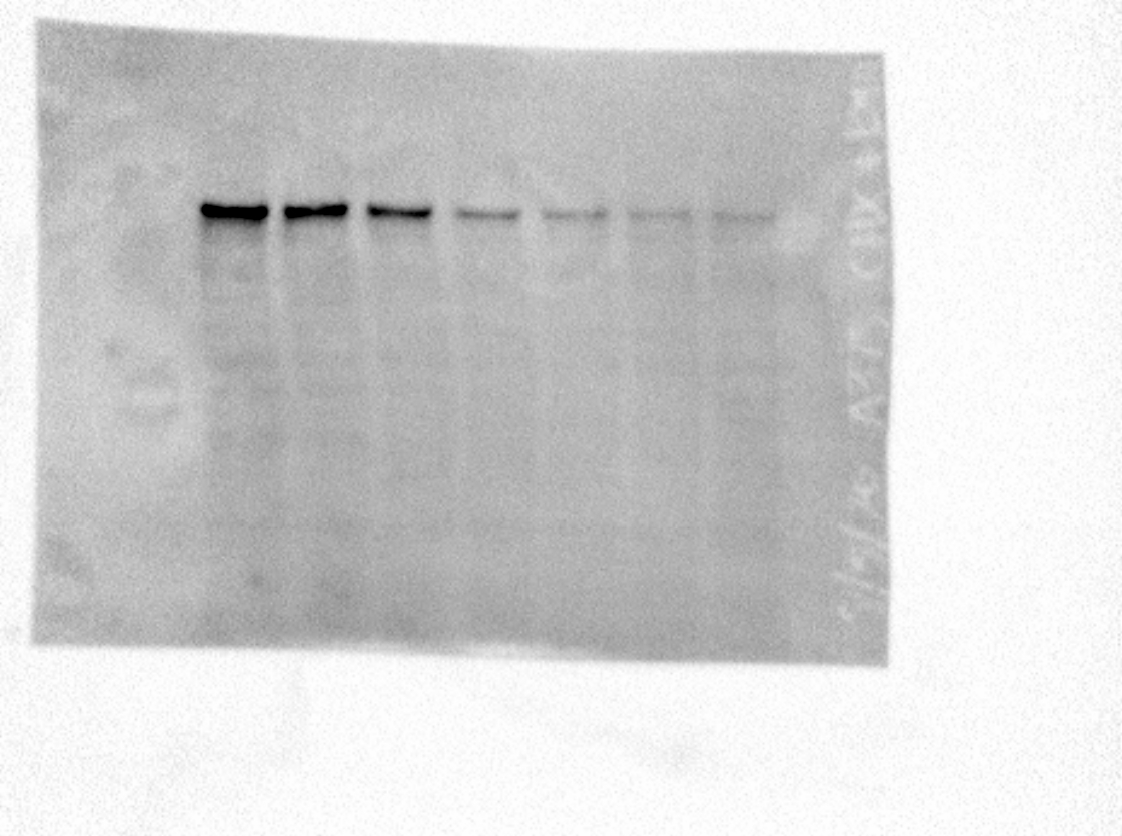

Supplement: Uncropped gels [file mmc3.zip › Uncropped gels/Fig2/2G/Steffie Pitts 2020-08-06 12hr 27min_Exposure_6.0sec A375 CHX +BMH RPA194.jpg]

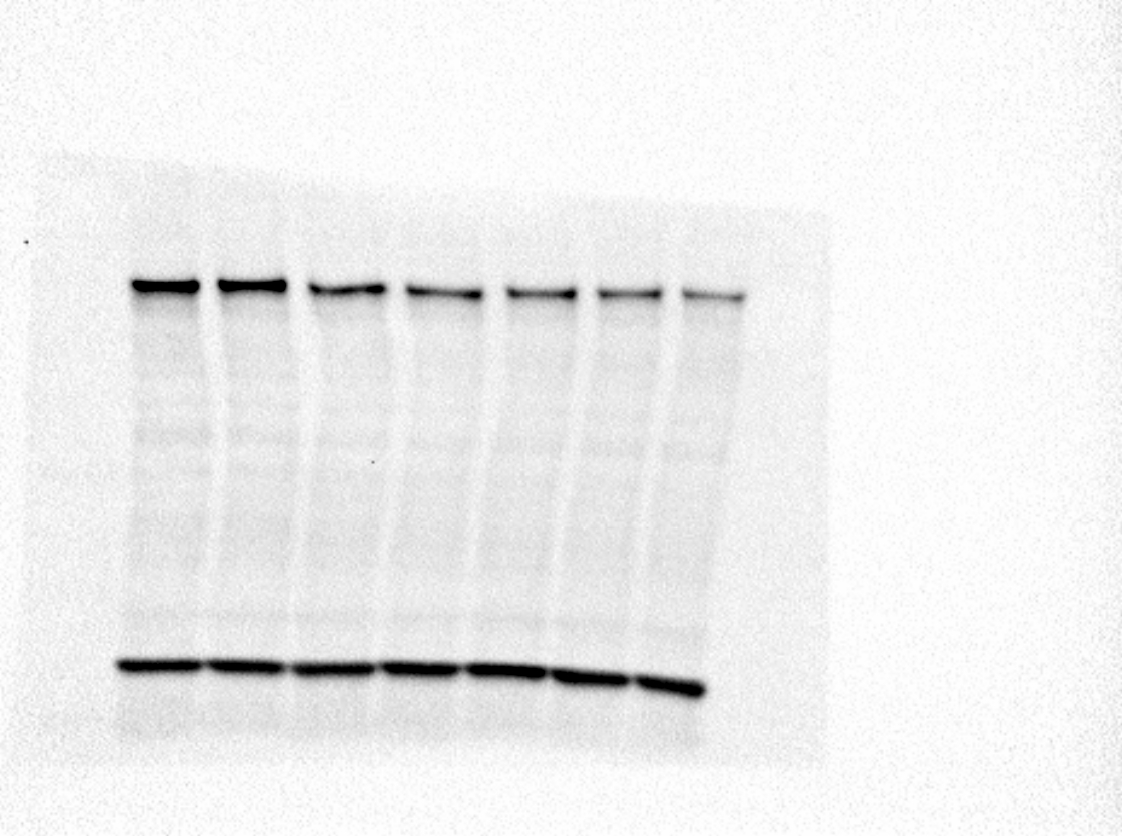

Supplement: Uncropped gels [file mmc3.zip › Uncropped gels/Fig2/2G/Steffie Pitts 2020-08-06 15hr 34min_Exposure_15.0sec shFBXL14 CHX + BMH GAPDH.jpg]

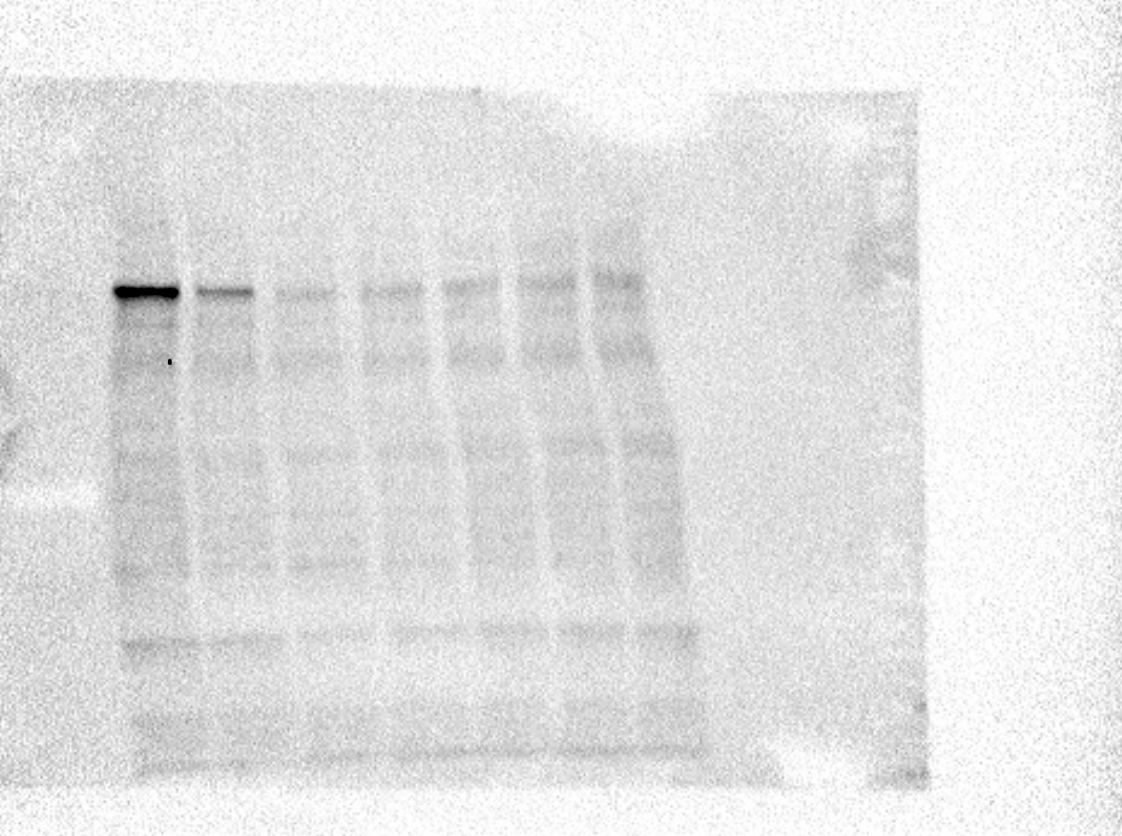

Supplement: Uncropped gels [file mmc3.zip › Uncropped gels/Fig2/2G/Steffie Pitts 2020-08-19 15hr 40min_Exposure_8.1sec FBX CHX +BMH blotting for RPA194.jpg]

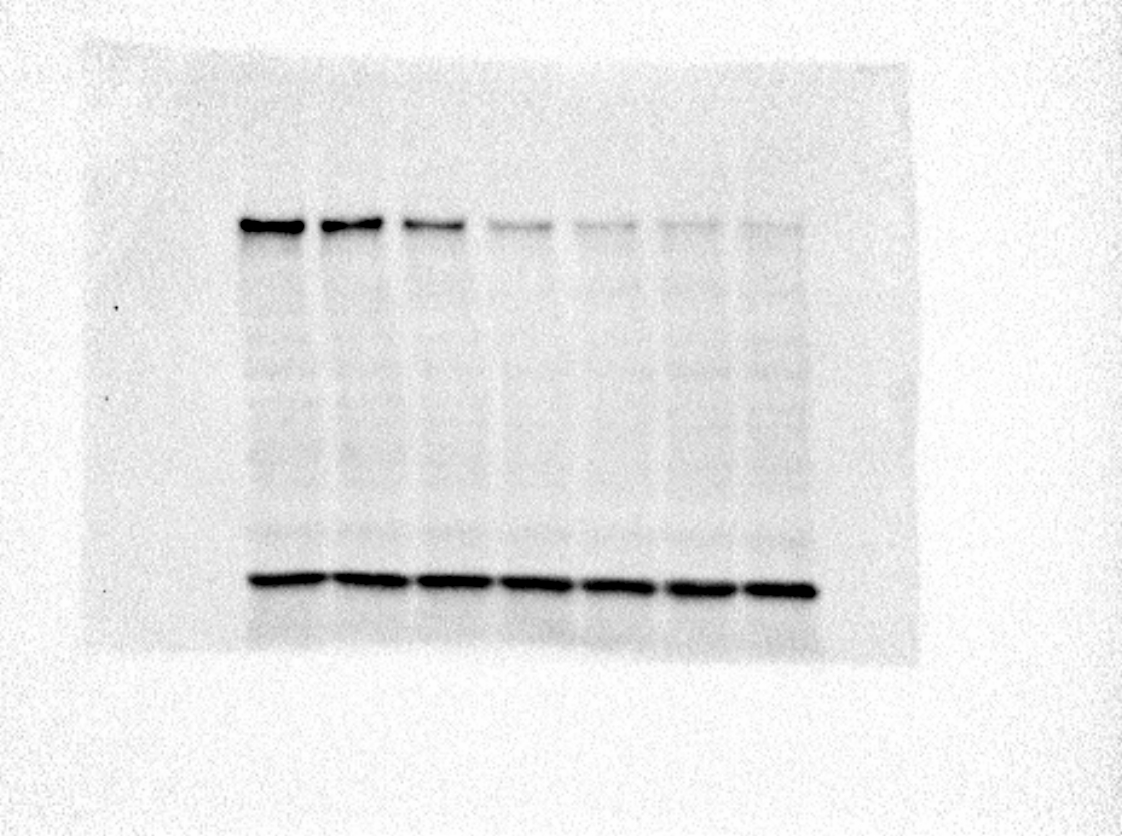

Supplement: Uncropped gels [file mmc3.zip › Uncropped gels/Fig2/2G/Steffie Pitts 2020-08-06 15hr 26min_Exposure_15.0sec A375 CHX +BMH GAPDH.jpg]

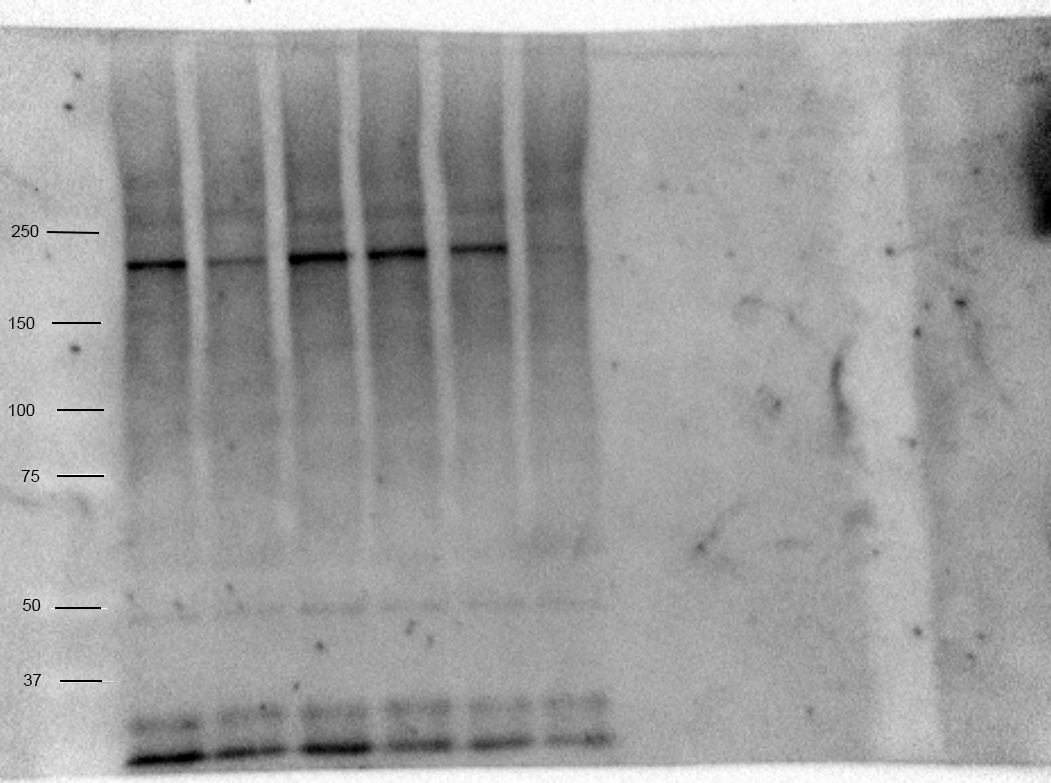

Supplement: Uncropped gels [file mmc3.zip › Uncropped gels/Fig2/2C/Steffie Pitts 2019-03-03 14hr 05min_Exposure_8.0sec Lysates blotting for RPA194 edited.tif]

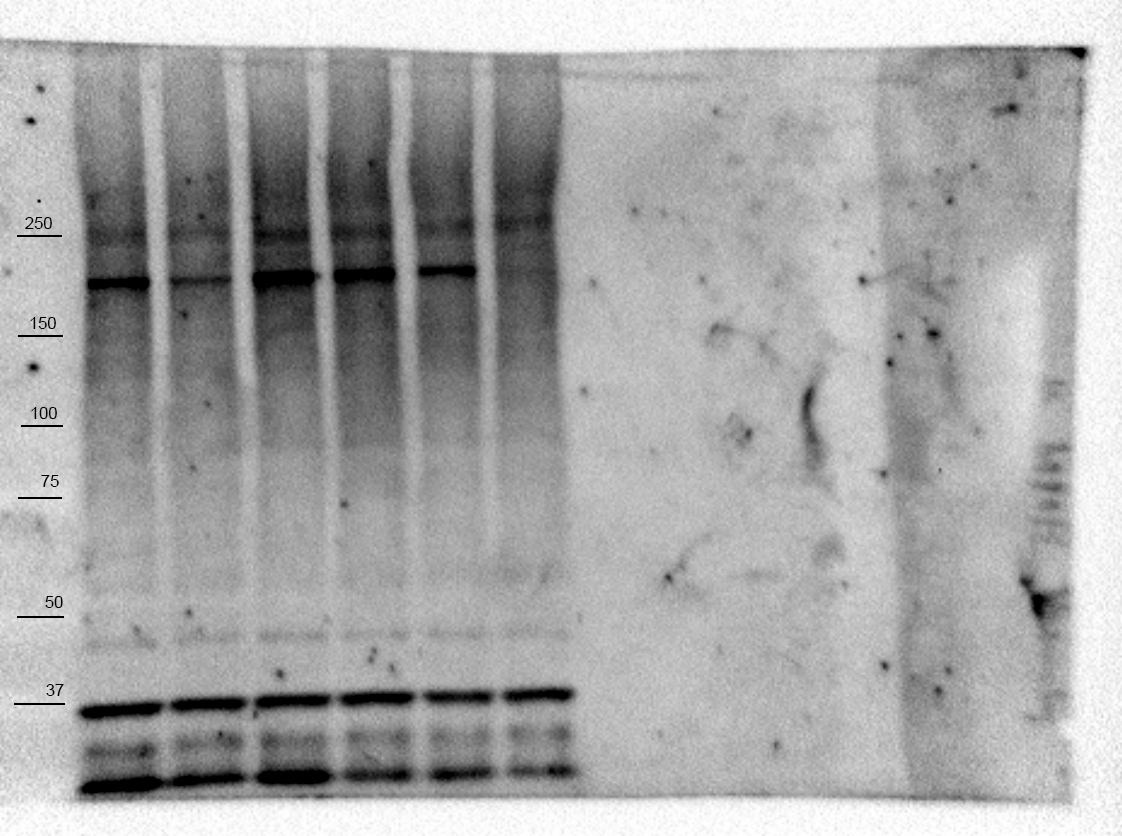

Supplement: Uncropped gels [file mmc3.zip › Uncropped gels/Fig2/2C/Steffie Pitts 2019-03-03 17hr 41min_Exposure_24.0sec Lysates blotting for GAPDH edited.tif]

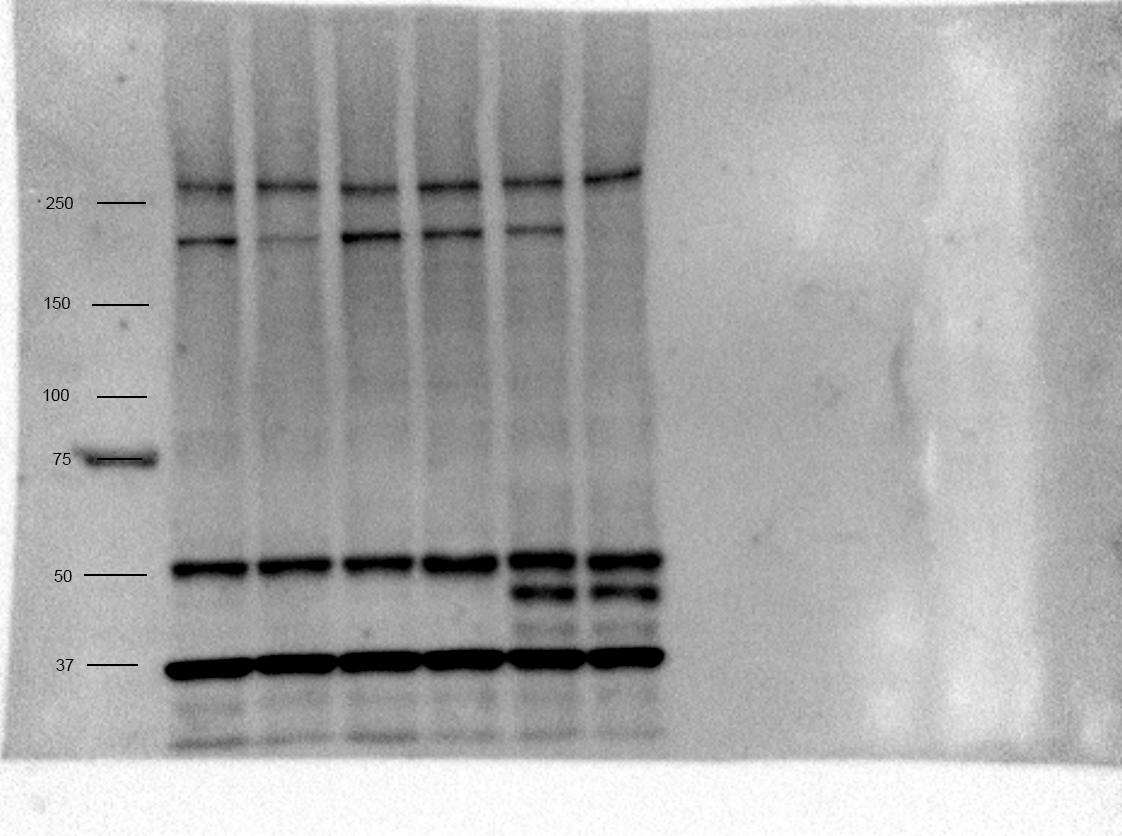

Supplement: Uncropped gels [file mmc3.zip › Uncropped gels/Fig2/2C/Steffie Pitts 2019-03-19 14hr 22min_Exposure_9.0sec blotting for Myc edited.tif]

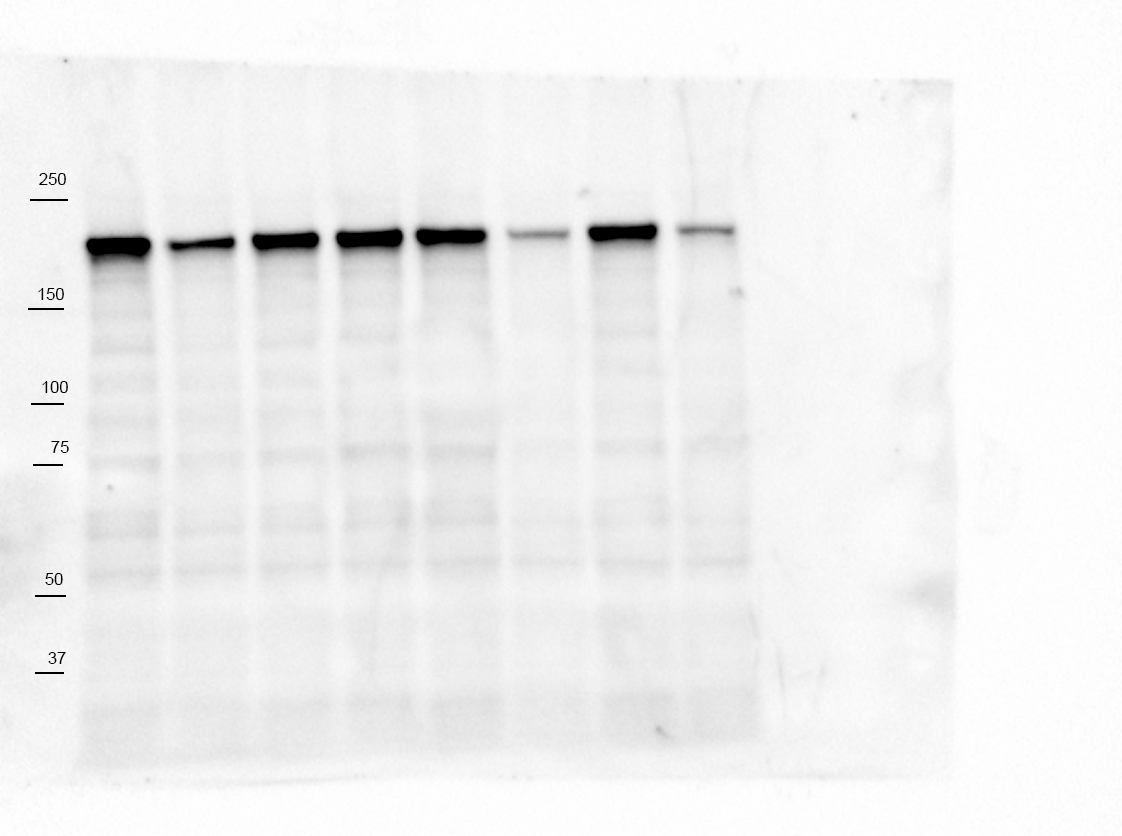

Supplement: Uncropped gels [file mmc3.zip › Uncropped gels/Fig2/2E/Steffie Pitts 2019-09-19 11hr 16min_Exposure_4.0sec blotting for RPA194 edited.tif]

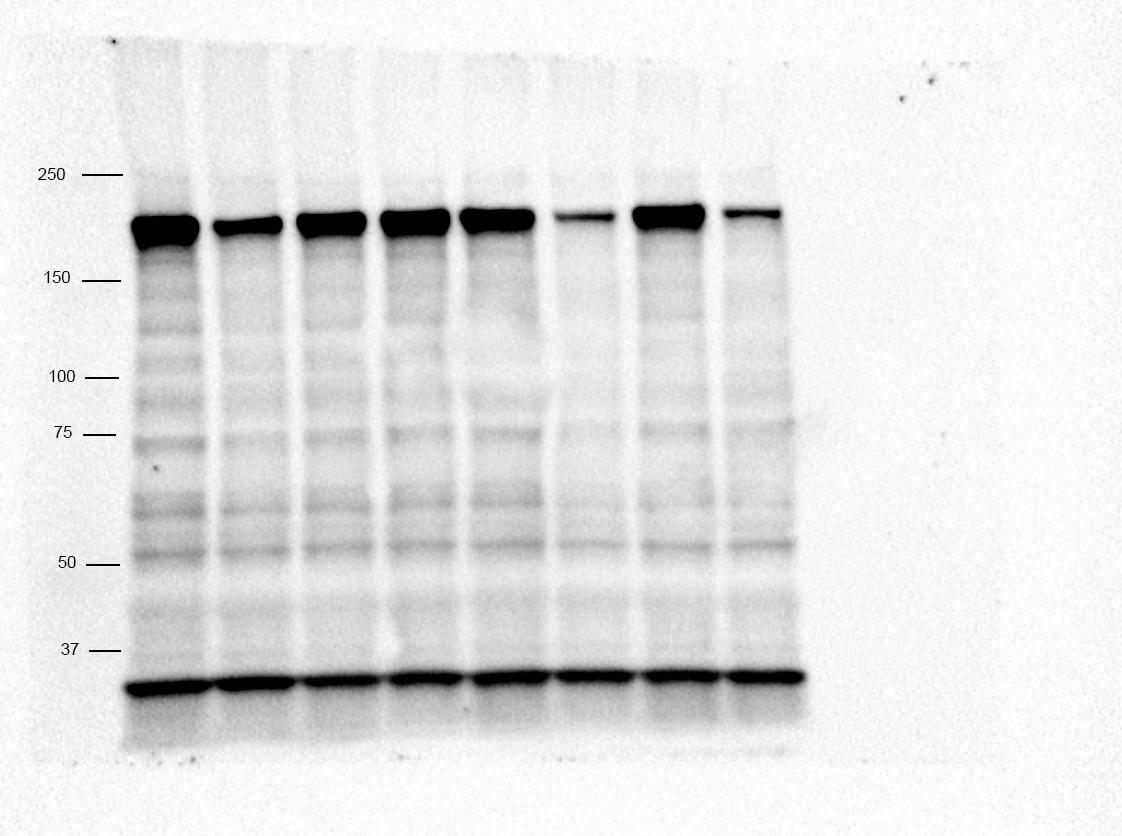

Supplement: Uncropped gels [file mmc3.zip › Uncropped gels/Fig2/2E/Steffie Pitts 2019-09-19 16hr 07min_Exposure_8.0sec RPA194 blotting for GAPDH edited.tif]

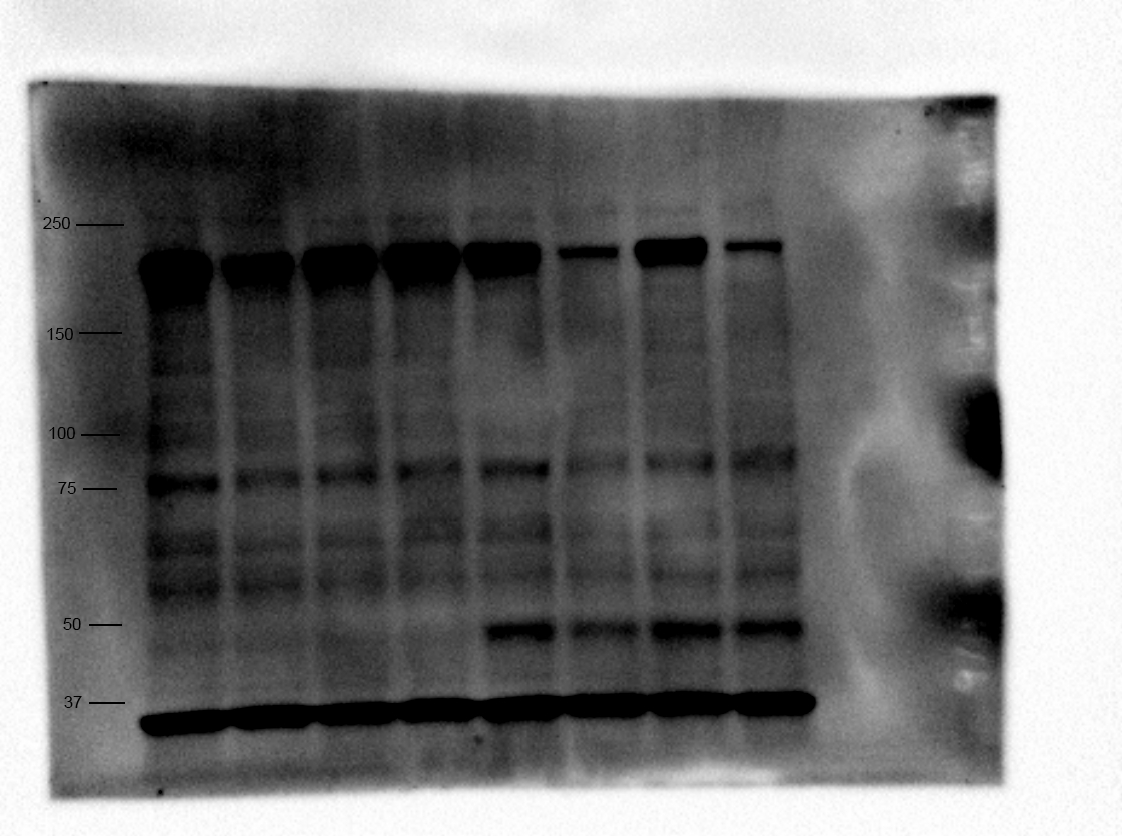

Supplement: Uncropped gels [file mmc3.zip › Uncropped gels/Fig2/2E/Steffie Pitts 2019-09-24 12hr 01min_Exposure_6.0sec blotting for Myc West Pico edited.tif]

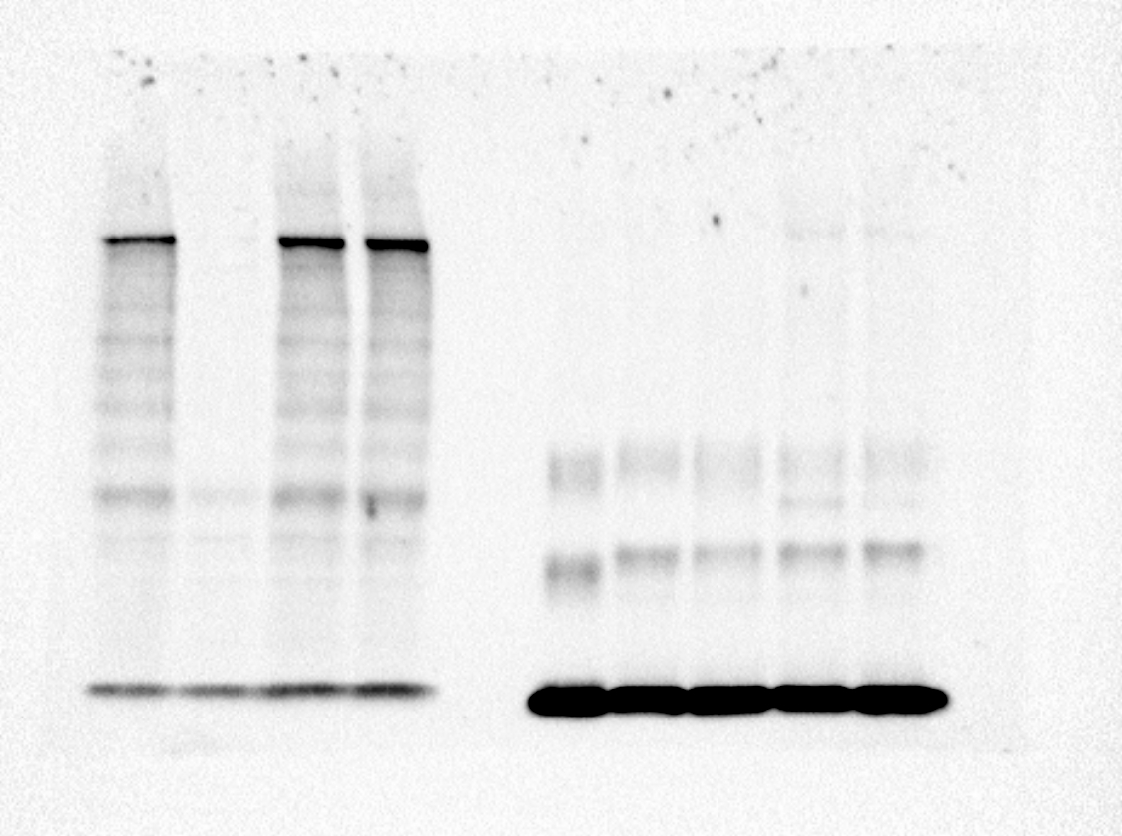

Supplement: Uncropped gels [file mmc3.zip › Uncropped gels/Fig3/3B/Steffie Pitts 2021-03-12_11h39m11s_Exposure_6.0sec In vitro Co-IP blotting for RPA194 lower exposure.jpg]

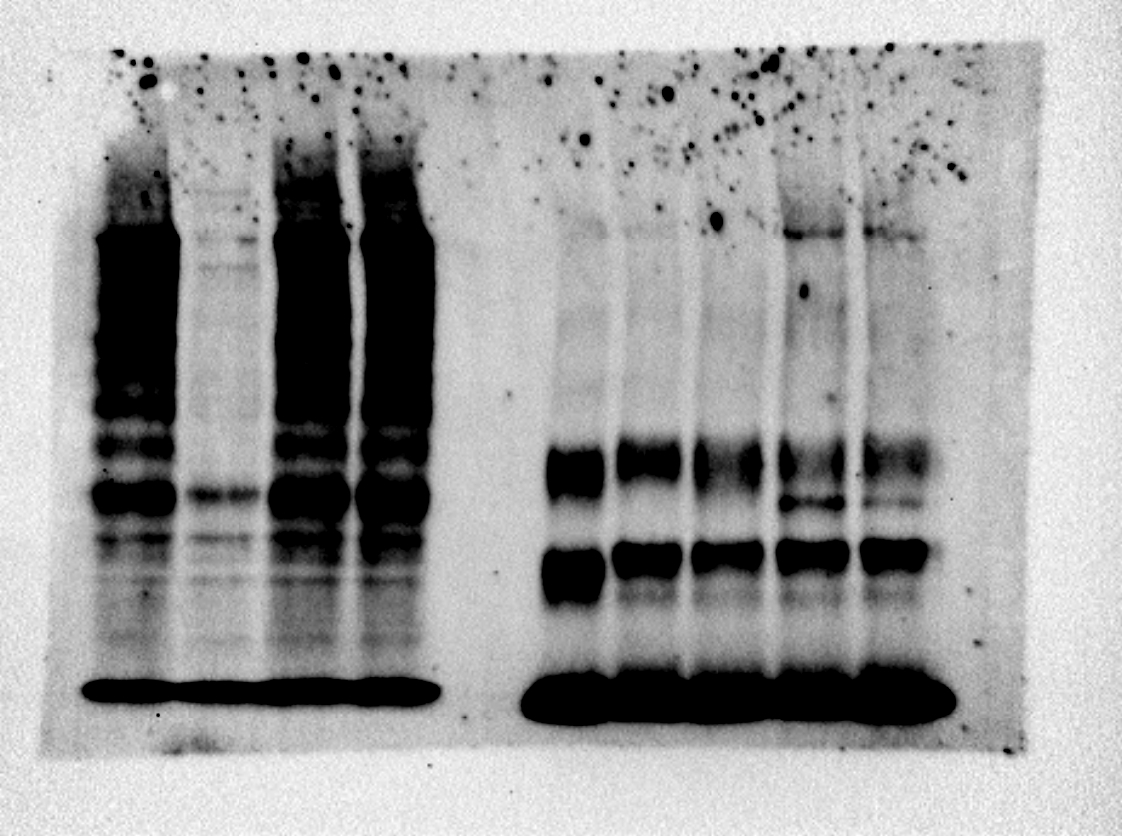

Supplement: Uncropped gels [file mmc3.zip › Uncropped gels/Fig3/3B/Steffie Pitts 2021-03-12_11h36m16s_Exposure_60.0sec In vitro Co-IP blotting for RPA194 high exposure.jpg]

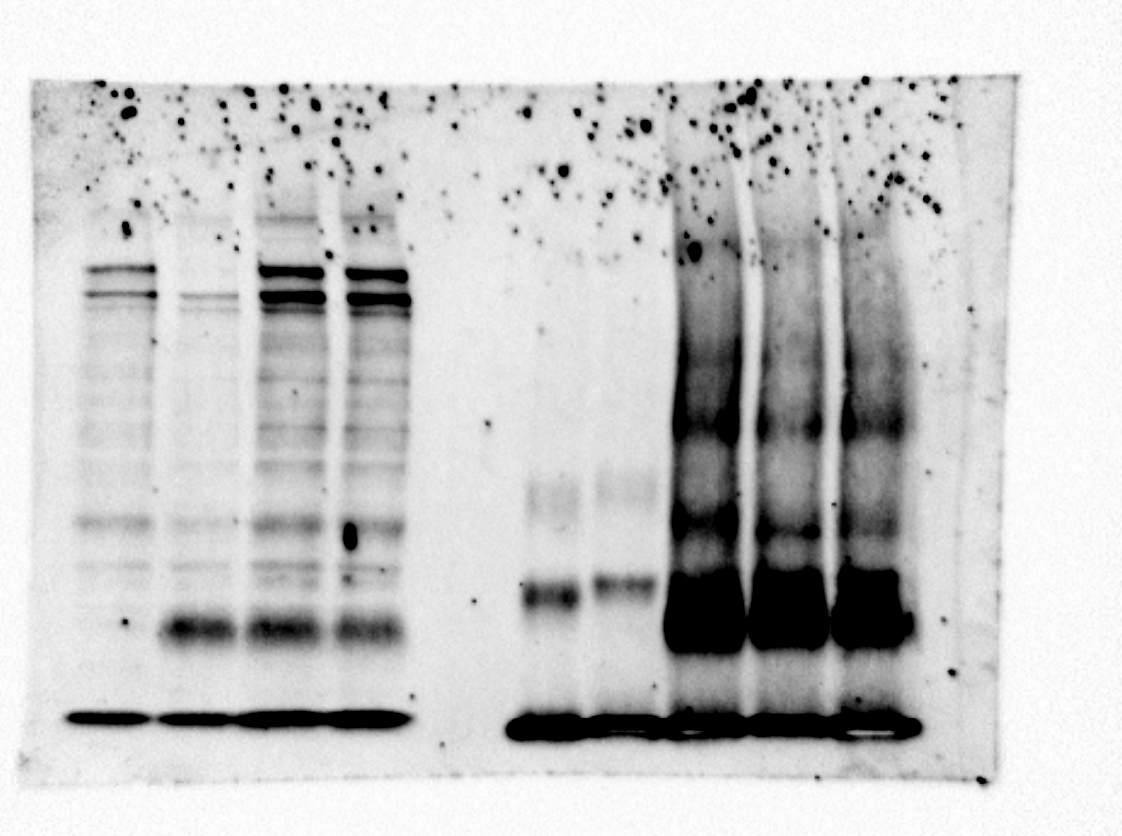

Supplement: Uncropped gels [file mmc3.zip › Uncropped gels/Fig3/3B/Steffie Pitts 2021-03-13_16h14m52s_Exposure_2.0sec In vitro Co-IP blotting for Myc 4A6 higher exposure.jpg]

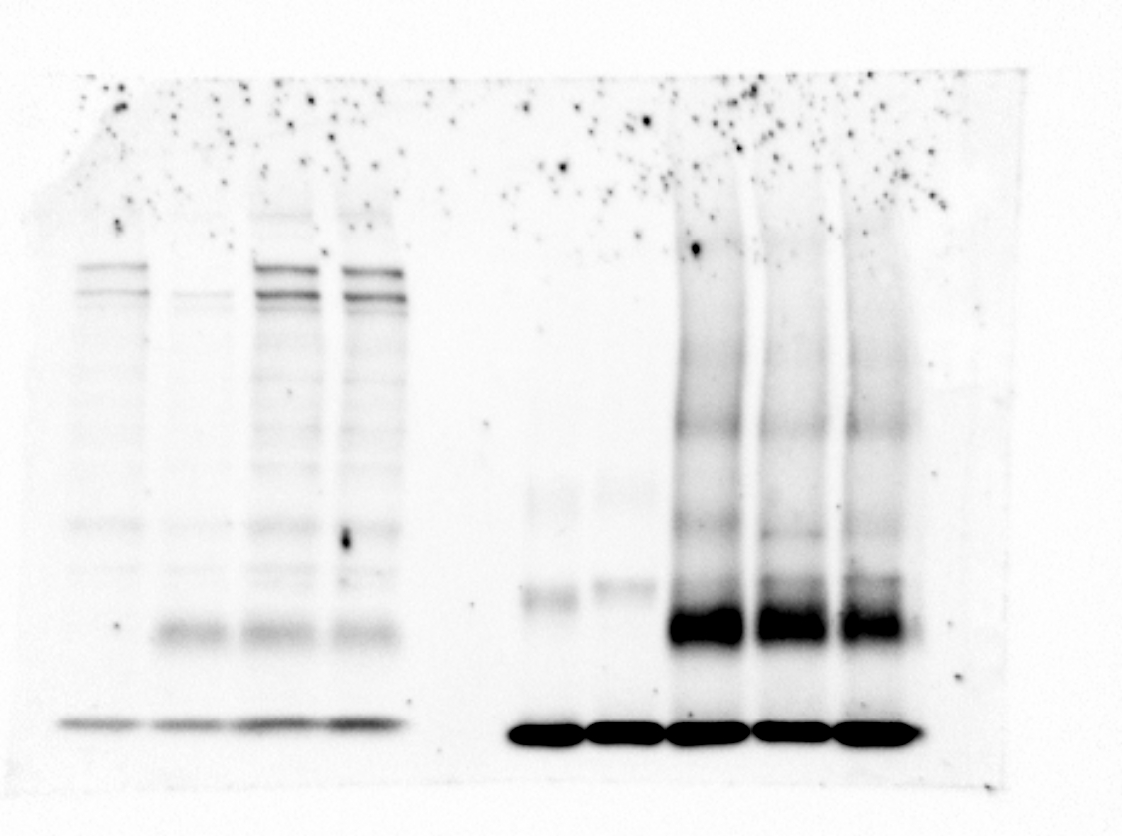

Supplement: Uncropped gels [file mmc3.zip › Uncropped gels/Fig3/3B/Steffie Pitts 2021-03-13_16h07m44s_Exposure_1.0sec In vitro Co-IP blotting for Myc 4A6.jpg]

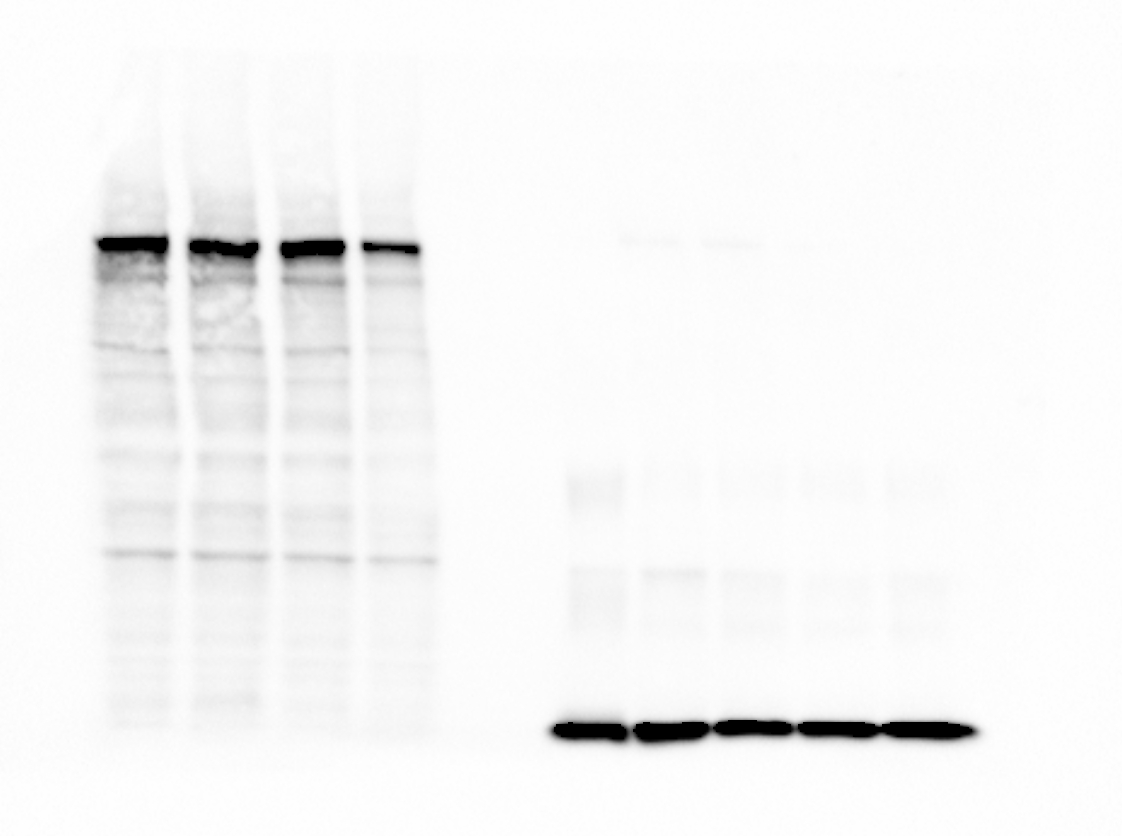

Supplement: Uncropped gels [file mmc3.zip › Uncropped gels/Fig3/3A/Steffie Pitts 2021-01-22 08hr 49min_Exposure_2.0sec In vivo Co-IP NP-40 BR3 blotting for RPA194 lower exposure.jpg]

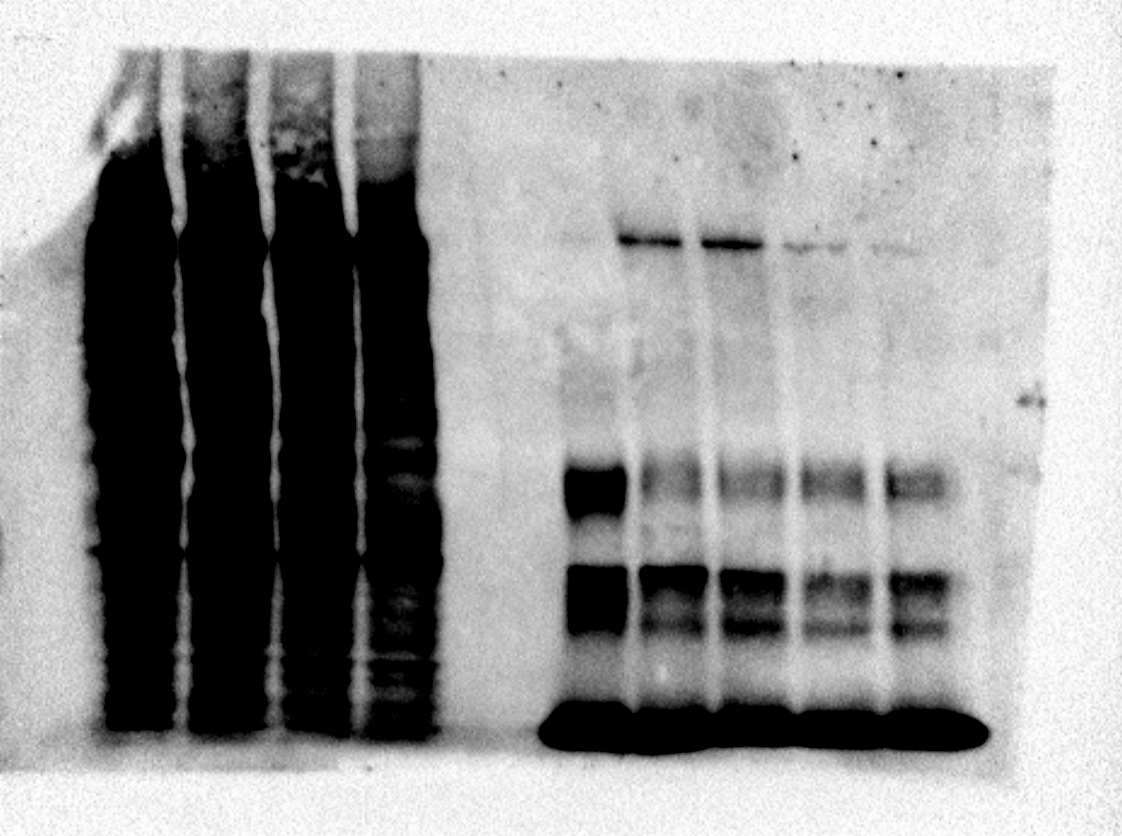

Supplement: Uncropped gels [file mmc3.zip › Uncropped gels/Fig3/3A/Steffie Pitts 2021-01-22 08hr 49min_Exposure_20.0sec In vivo Co-IP NP-40 BR3 blotting for RPA194 higher exposure.jpg]

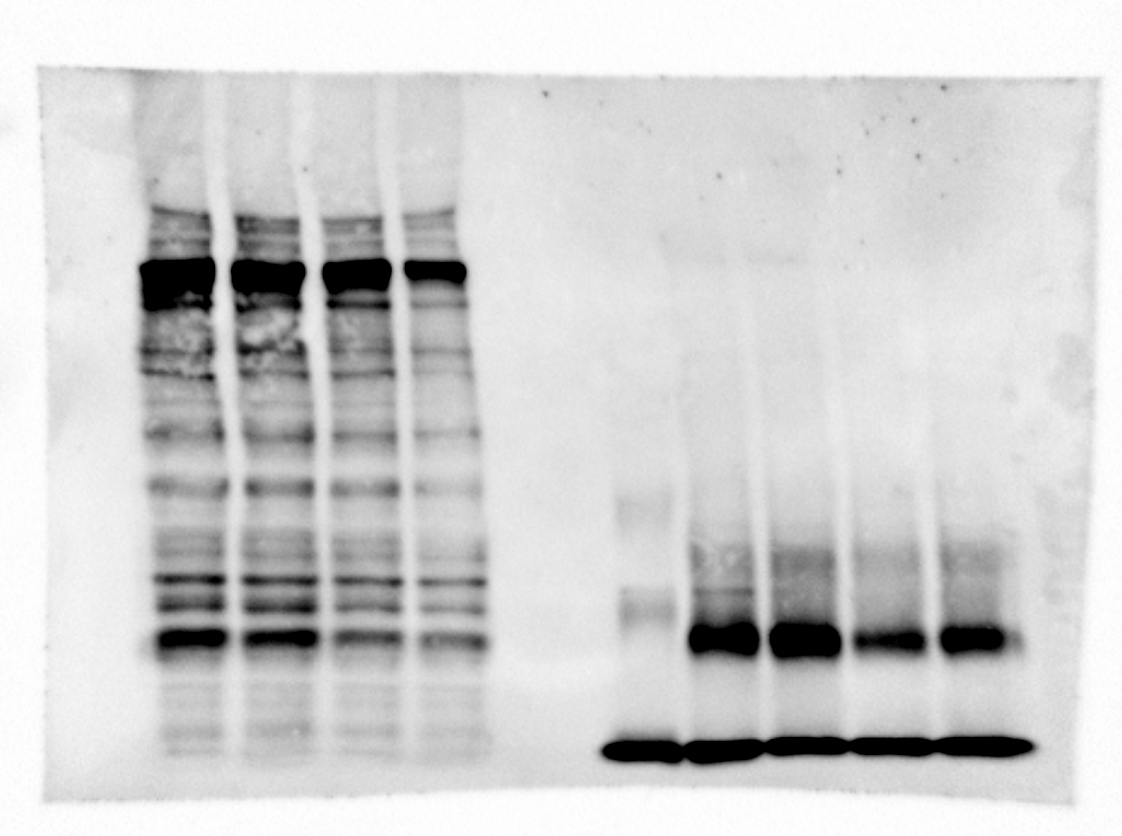

Supplement: Uncropped gels [file mmc3.zip › Uncropped gels/Fig3/3A/Steffie Pitts 2021-01-23 17hr 56min_Exposure_2.0sec In vivo Co-IP NP-40 BR3 blotting for Myc 4A6.jpg]

**Original gels**


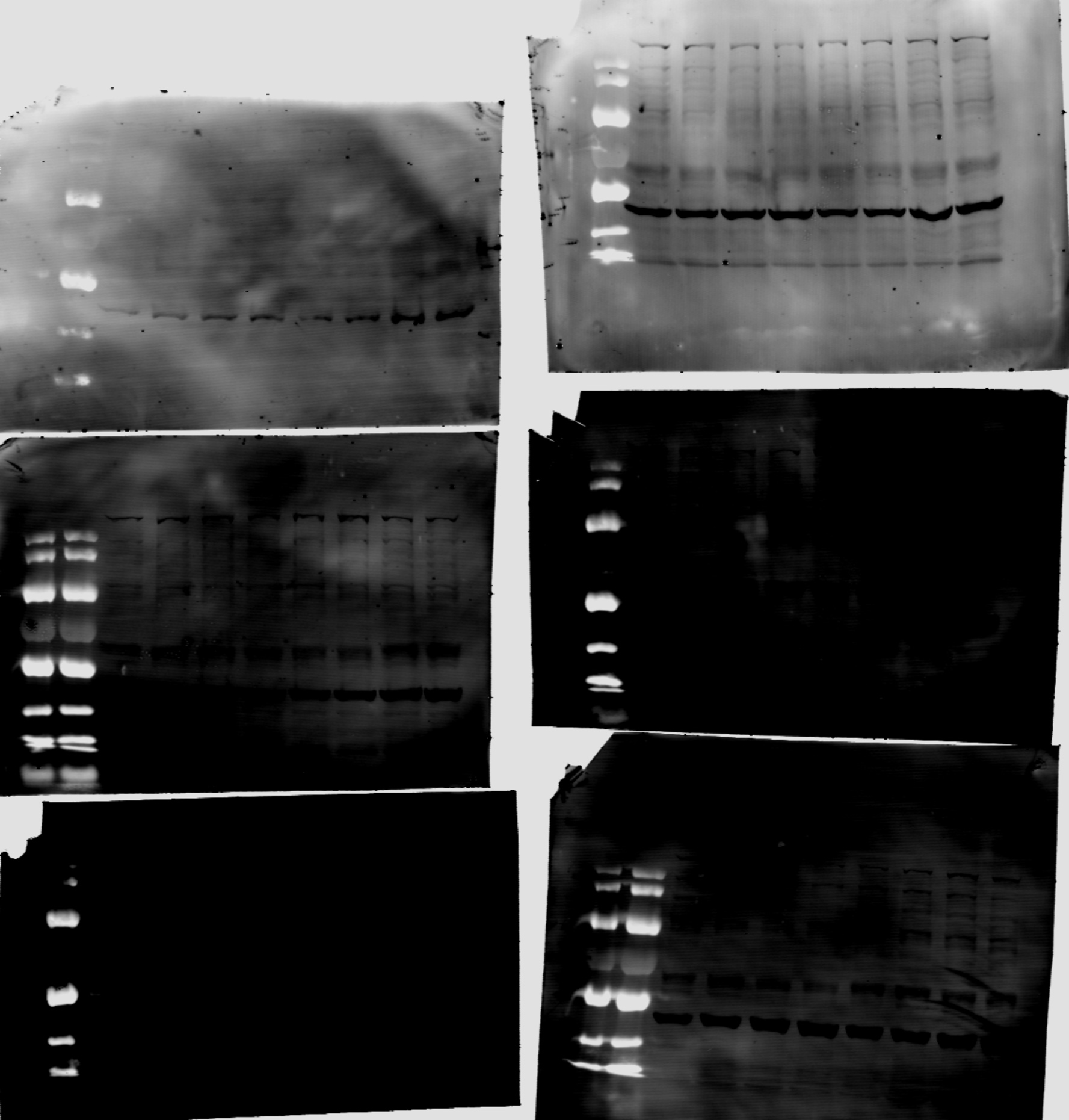

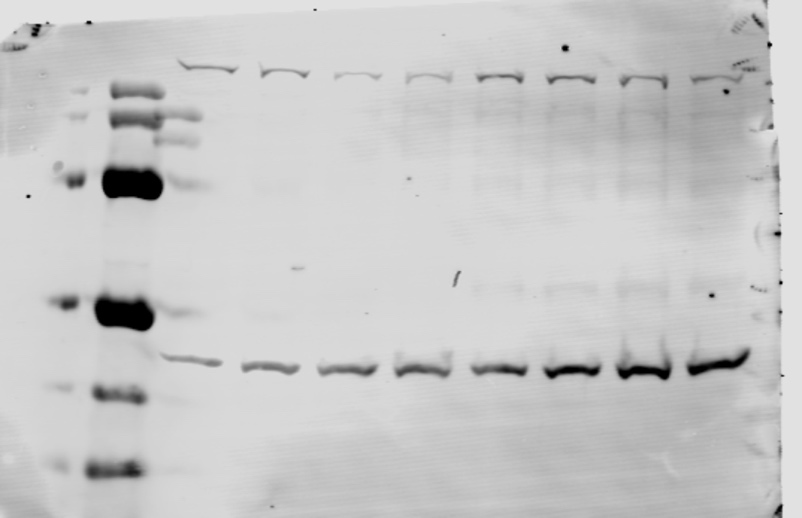
Wild type: Mutant:

0 30 60 75

0 30 60 75

50 μM BMH-21

Vehicle

A190

PGK1

Time (min)


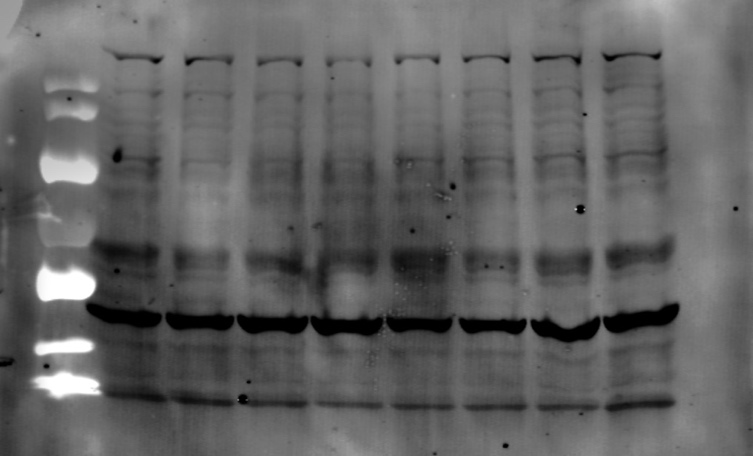

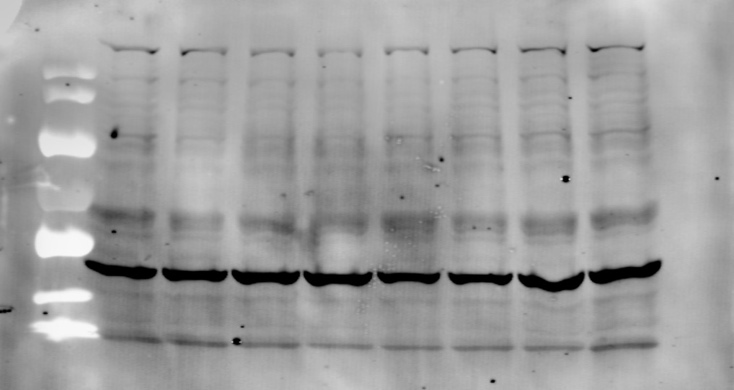


0 30 60 75

0 30 60 75

50 μM BMH-21

Vehicle

A190

PGK1


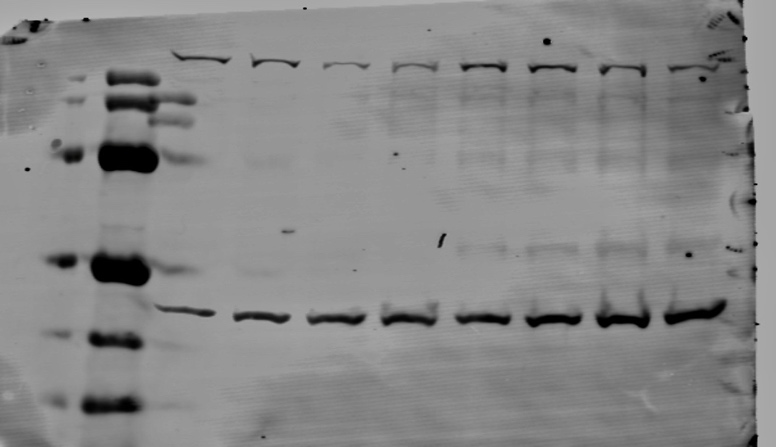

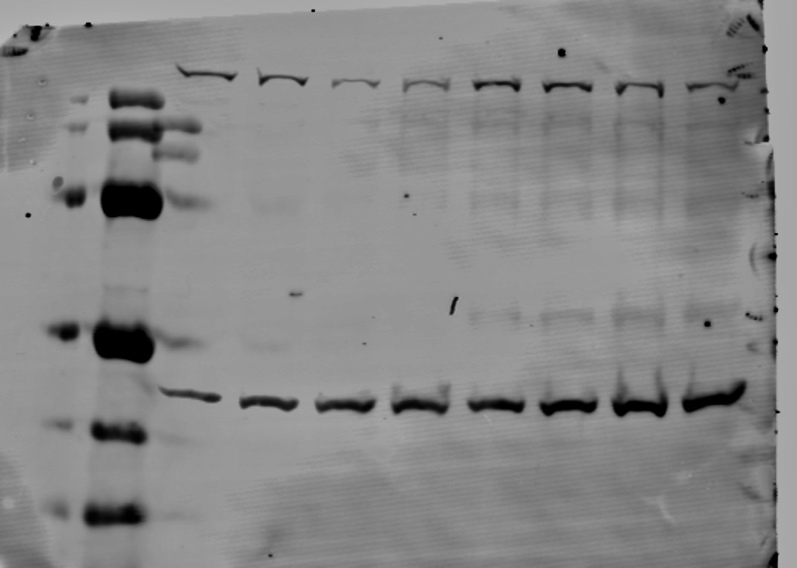


Time (min)

Supplement: Uncropped gels [file mmc3.zip › Uncropped gels/Fig3/3F/3F Representative Gel.docx]

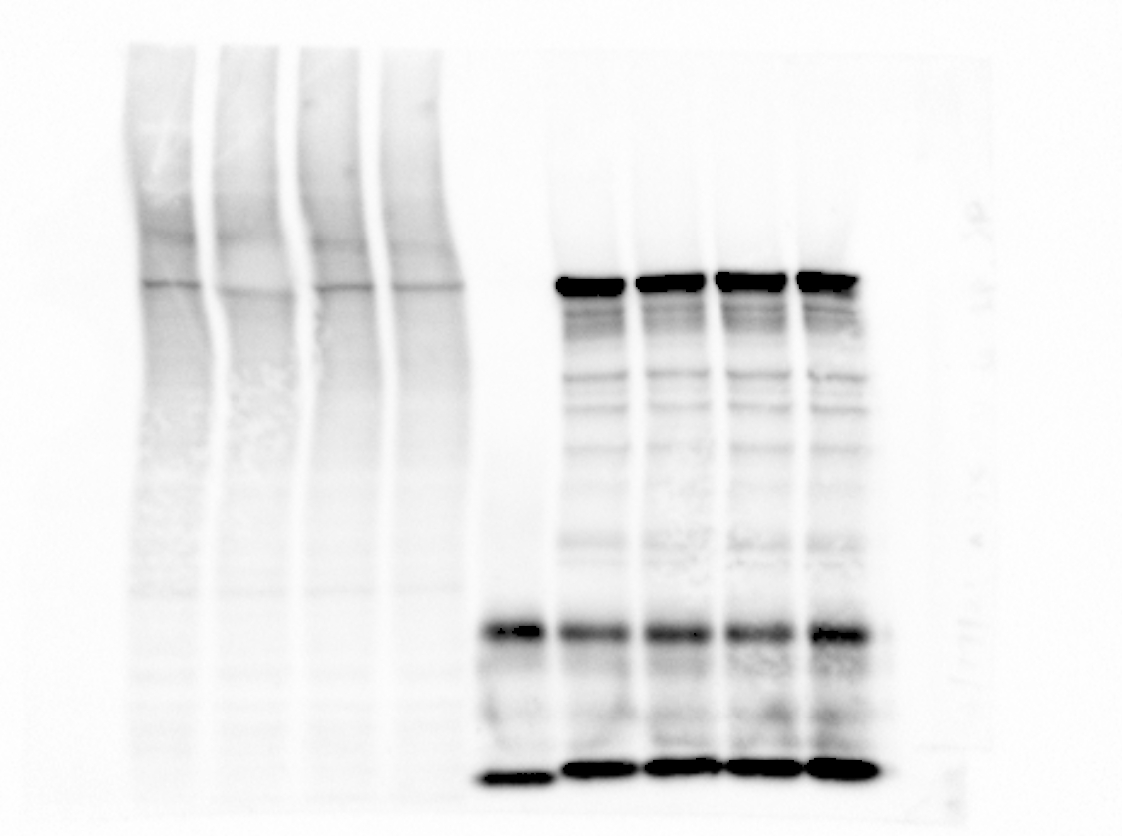

Supplement: Uncropped gels [file mmc3.zip › Uncropped gels/Fig3/C/Steffie Pitts 2021-06-30_18h54m04s_Exposure_1.0sec A375 EV and shFBXL14 Co-IP blotting for RPA194 lower exposure.jpg]

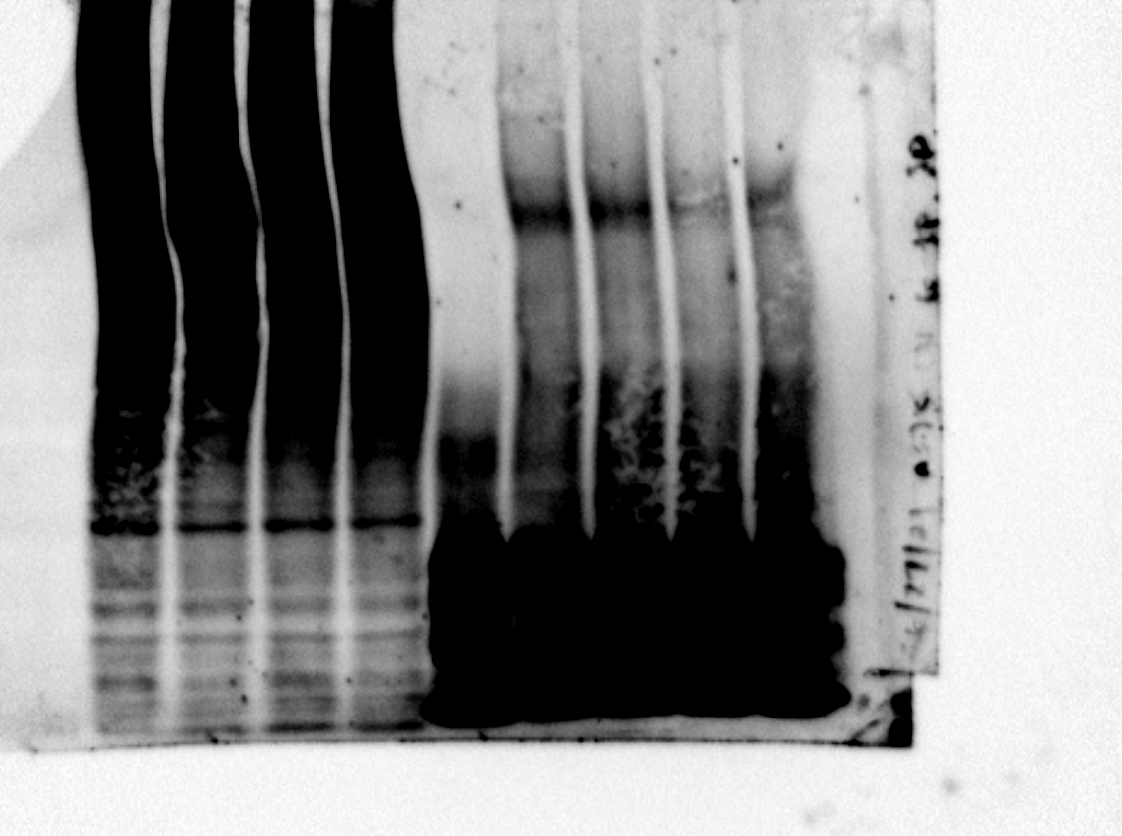

Supplement: Uncropped gels [file mmc3.zip › Uncropped gels/Fig3/C/Steffie Pitts 2021-06-29_15h49m20s_Exposure_8.0sec A375 EV and shFBXL14 Co-IP blotting for FK2 higher exposure.jpg]
